# Supplementary material for: Effect of chronic unpredicted mild stress-induced depression on clopidogrel pharmacokinetics in rats
Source: PeerJ. 2022 Oct 3;10:e14111. doi: 10.7717/peerj.14111 (PMC9536304; doi:10.7717/peerj.14111)

Sample Name: "MEOH" Sample ID: "" File: "20220801-1.wiff"
Peak Name: "OHMPT" Mass(es): "235.200/150.200 Da"
Comment: "" Annotation: ""

```
Sample Index:       3
Sample Type:        Unknown
Concentration:      N/A
Calculated Conc:    No Intercept
Acq. Date:          8/1/2022
Acq. Time:          11:27:52 AM

Modified:           No
```

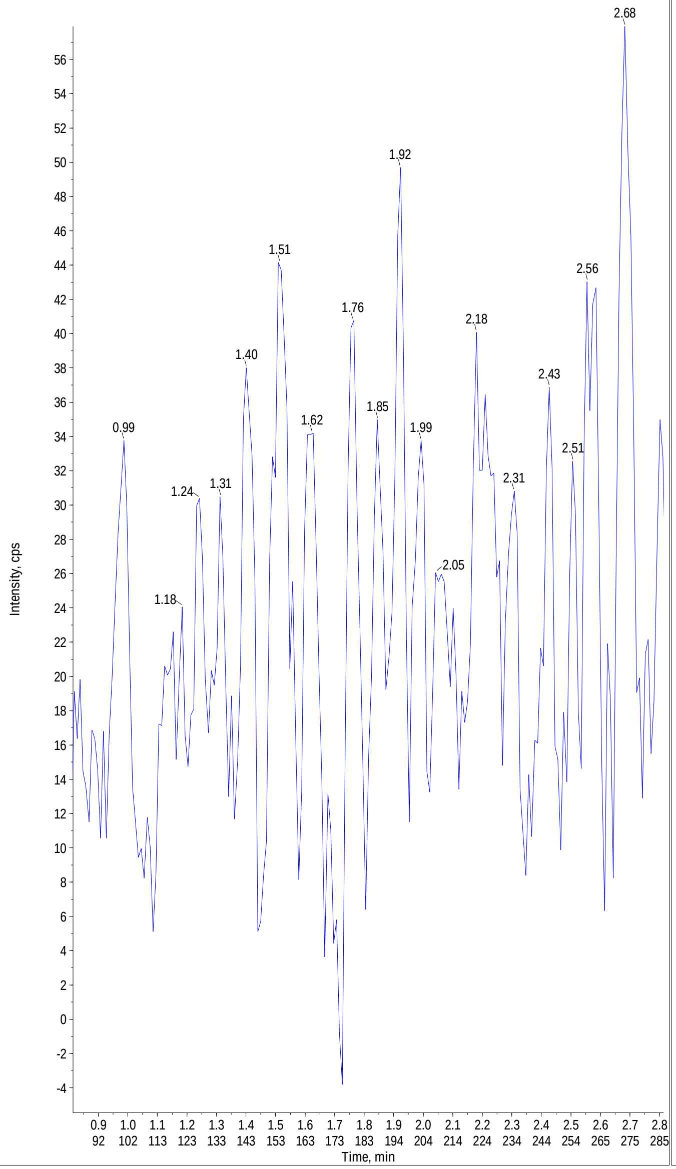

Sample Name: "MEOH" Sample ID: "" File: "20220801-1.wiff"
Peak Name: "DXP(IS)" Mass(es): "285.500/154.100 Da"
Comment: "" Annotation: ""

```
Sample Index:       3
Sample Type:        Unknown
Concentration:      1.00    ng/mL
Calculated Conc:    N/A
Acq. Date:          8/1/2022
Acq. Time:          11:27:52 AM

Modified:           No
```

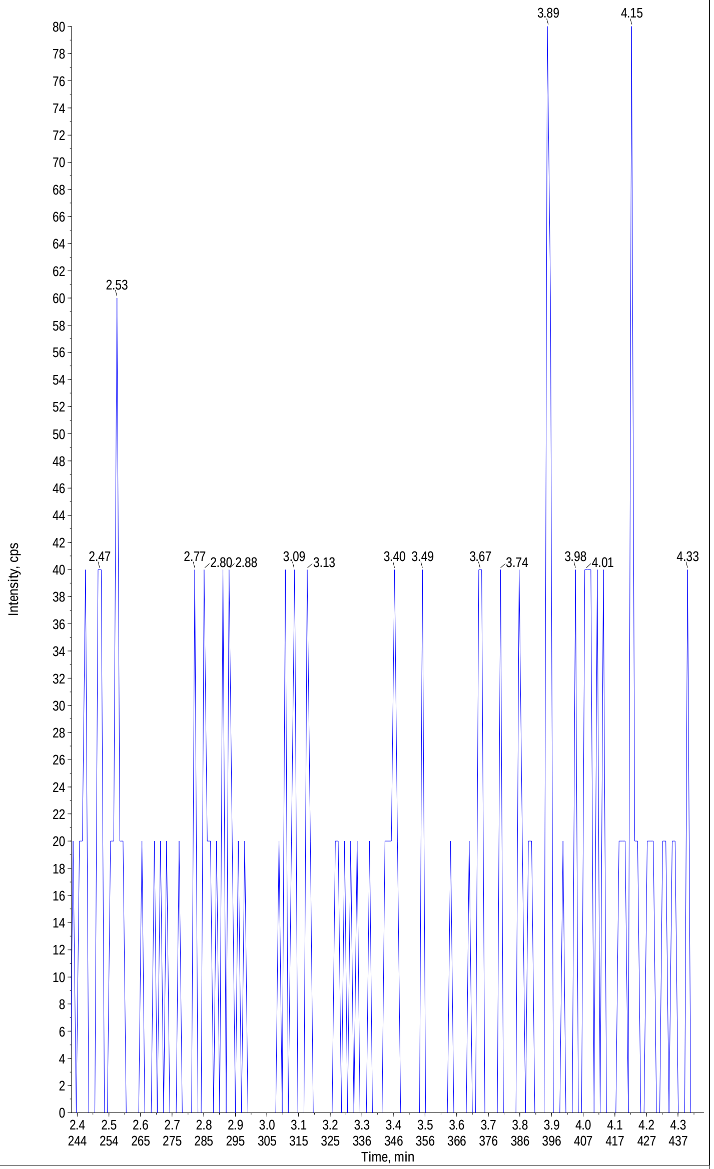

Sample Name: "KB1" Sample ID: "" File: "20220801-1.wiff"
Peak Name: "OHMPT" Mass(es): "235.200/150.200 Da"
Comment: "" Annotation: ""

**Sample Index:**	4
**Sample Type:**	Unknown
**Concentration:**	N/A
**Calculated Conc:**	No Intercept
**Acq. Date:**	8/1/2022
**Acq. Time:**	11:32:55 AM
**Modified:**	No

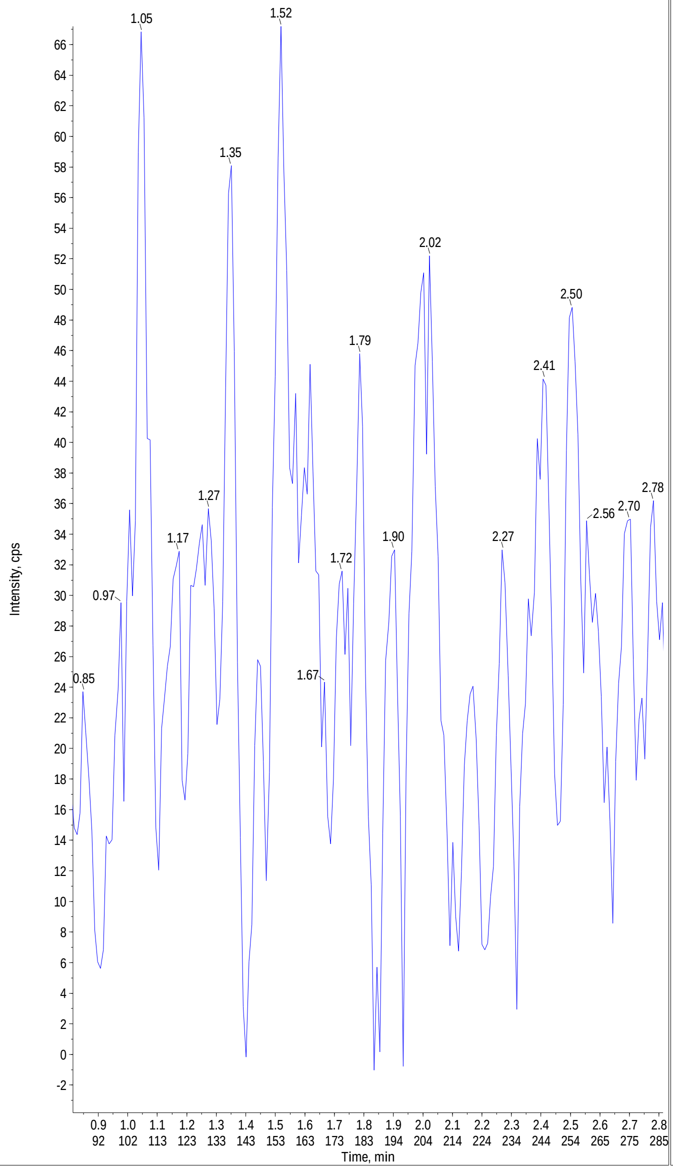

Sample Name: "KB1" Sample ID: "" File: "20220801-1.wiff"
Peak Name: "DXP(IS)" Mass(es): "285.500/154.100 Da"
Comment: "" Annotation: ""

**Sample Index:**	4
**Sample Type:**	Unknown
**Concentration:**	1.00 ng/mL
**Calculated Conc:**	N/A
**Acq. Date:**	8/1/2022
**Acq. Time:**	11:32:55 AM
**Modified:**	No

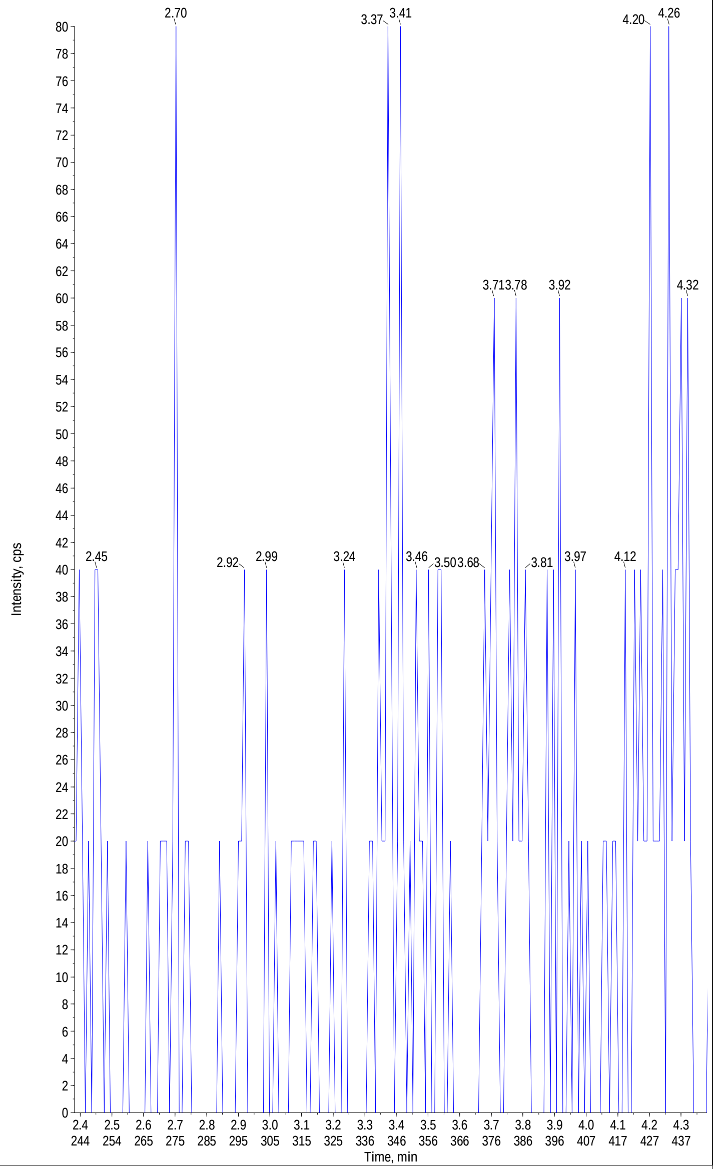

Sample Name: "KB2" Sample ID: "" File: "20220801-1.wiff"
Peak Name: "OHMPT" Mass(es): "235.200/150.200 Da"
Comment: "" Annotation: ""

Sample Index:	5
Sample Type:	Unknown
Concentration:	N/A
Calculated Conc:	No Intercept
Acq. Date:	8/1/2022
Acq. Time:	11:37:57 AM
Modified:	No

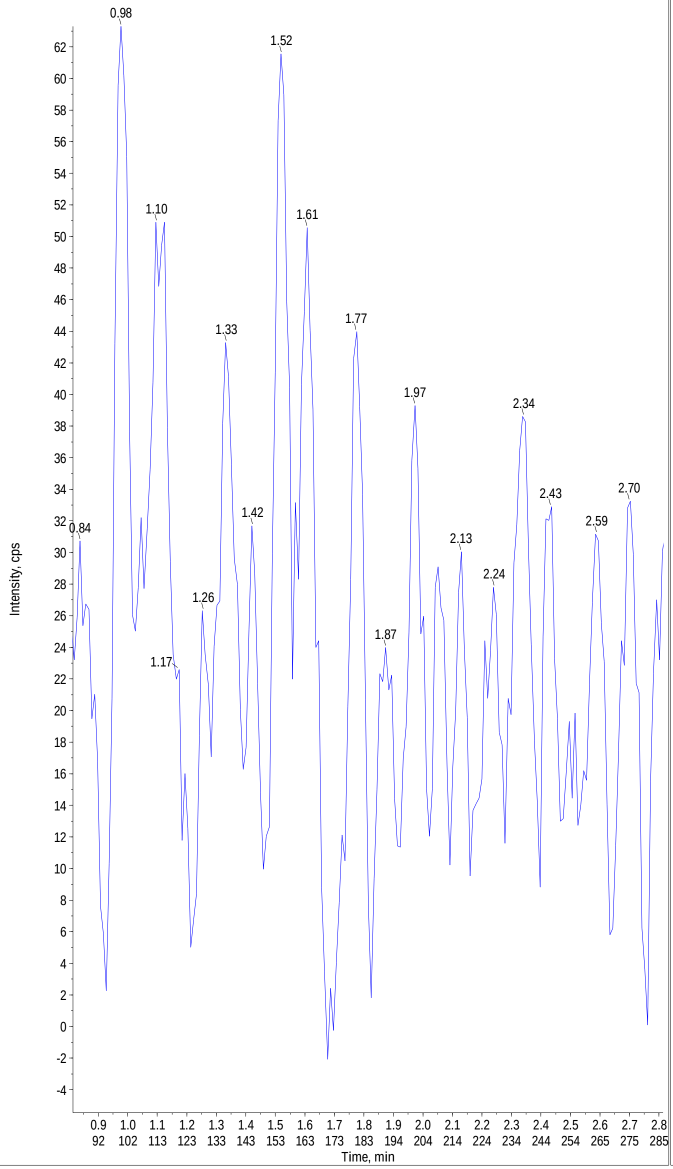

Sample Name: "KB2" Sample ID: "" File: "20220801-1.wiff"
Peak Name: "DXP(IS)" Mass(es): "285.500/154.100 Da"
Comment: "" Annotation: ""

Sample Index:	5
Sample Type:	Unknown
Concentration:	1.00 ng/mL
Calculated Conc:	N/A
Acq. Date:	8/1/2022
Acq. Time:	11:37:57 AM
Modified:	No

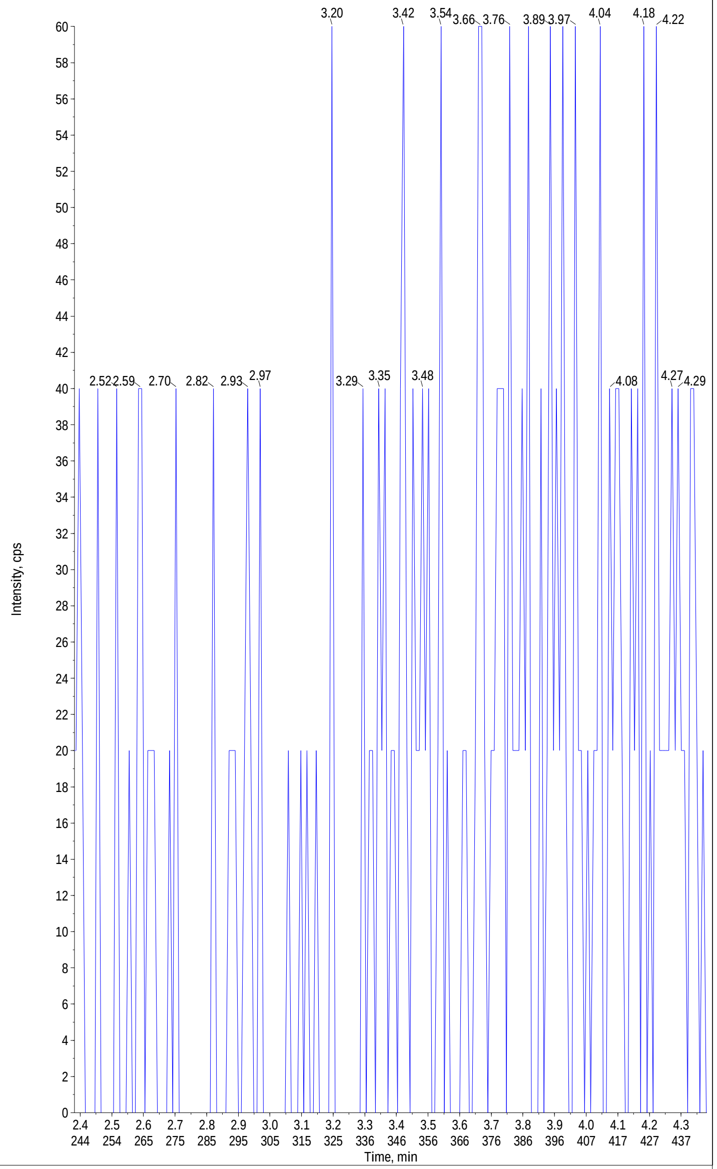

Sample Name: "KB3"   Sample ID: ""   File: "20220801-1.wiff"
Peak Name: "OHMPT"   Mass(es): "235.200/150.200 Da"
Comment: ""   Annotation: ""

Sample Index: 6
Sample Type: Unknown
Concentration: N/A
Calculated Conc: No Intercept
Acq. Date: 8/1/2022
Acq. Time: 11:42:59 AM

Modified: No

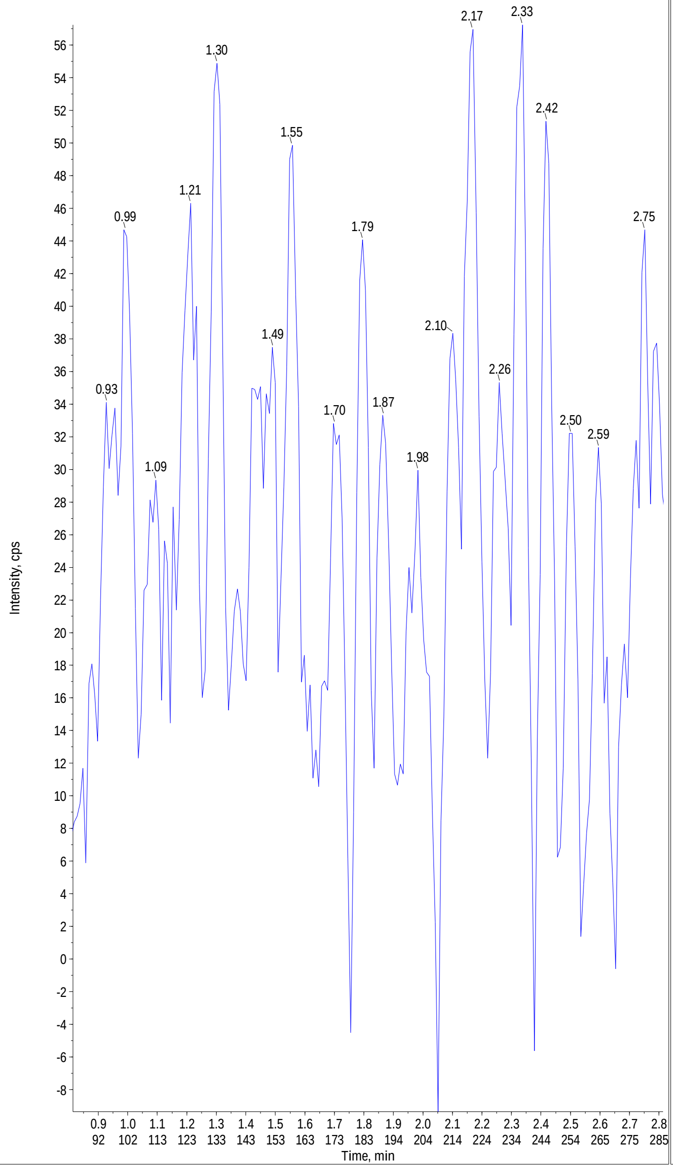

Sample Name: "KB3"   Sample ID: ""   File: "20220801-1.wiff"
Peak Name: "DXP(IS)"   Mass(es): "285.500/154.100 Da"
Comment: ""   Annotation: ""

Sample Index: 6
Sample Type: Unknown
Concentration: 1.00 ng/mL
Calculated Conc: N/A
Acq. Date: 8/1/2022
Acq. Time: 11:42:59 AM

Modified: No

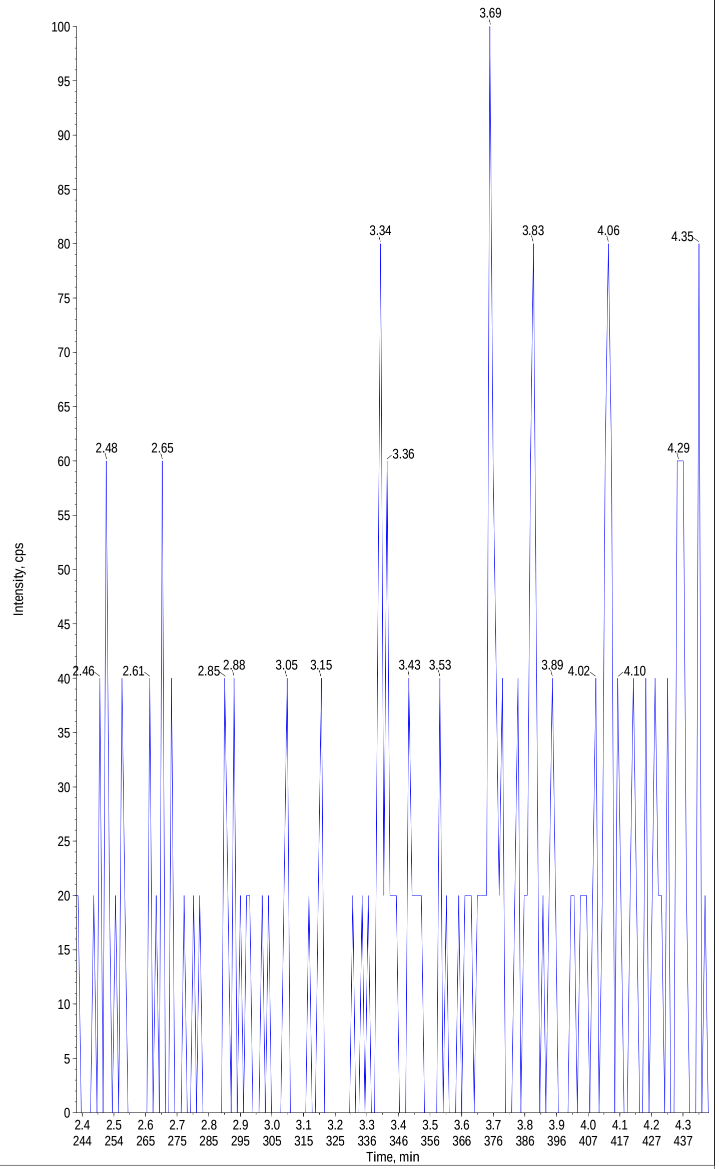

Sample Name: "KB4"   Sample ID: ""   File: "20220801-1.wiff"
Peak Name: "OHMPT"   Mass(es): "235.200/150.200 Da"
Comment: ""   Annotation: ""

```
Sample Index:        7
Sample Type:      Unknown
Concentration:       N/A
Calculated Conc: No Intercept
Acq. Date:       8/1/2022
Acq. Time:       11:48:02 AM

Modified:            No
```

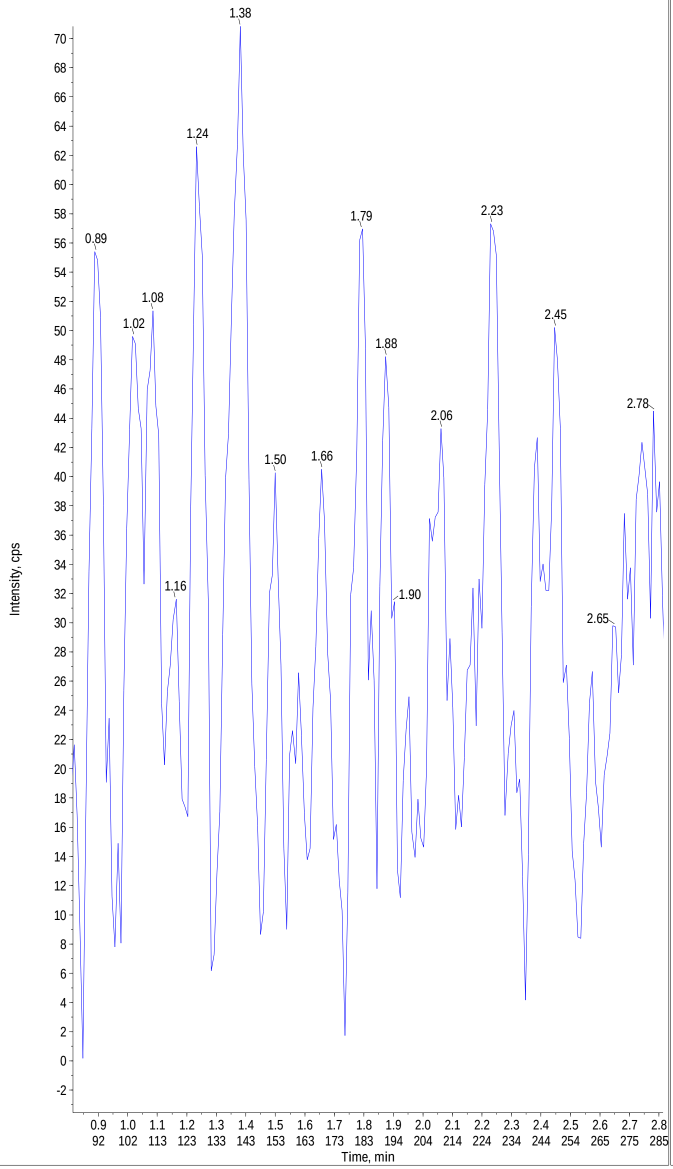

Sample Name: "KB4"   Sample ID: ""   File: "20220801-1.wiff"
Peak Name: "DXP(IS)"   Mass(es): "285.500/154.100 Da"
Comment: ""   Annotation: ""

```
Sample Index:        7
Sample Type:      Unknown
Concentration:       1.00    ng/mL
Calculated Conc:     N/A
Acq. Date:       8/1/2022
Acq. Time:       11:48:02 AM

Modified:            No
```

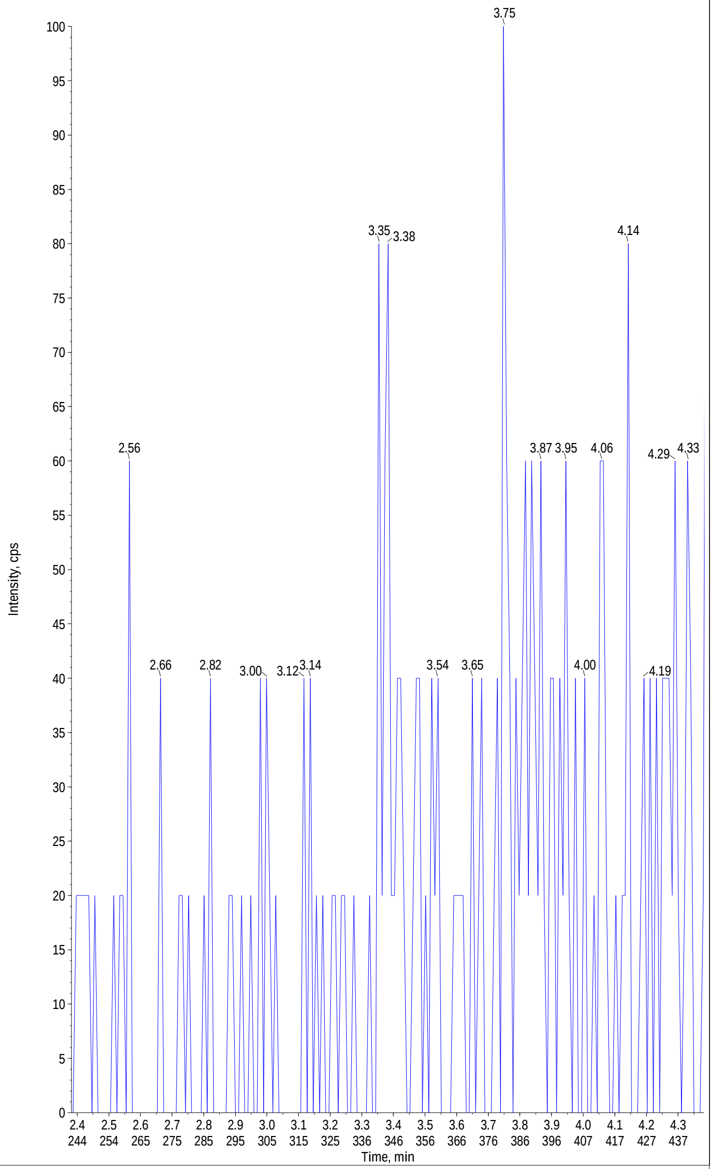

Sample Name: "KB5"   Sample ID: ""   File: "20220801-1.wiff"
Peak Name: "OHMPT"   Mass(es): "235.200/150.200 Da"
Comment: ""   Annotation: ""

**Sample Index:**	**8**
**Sample Type:**	**Unknown**
**Concentration:**	**N/A**
**Calculated Conc:**	**No Intercept**
**Acq. Date:**	**8/1/2022**
**Acq. Time:**	**11:53:05 AM**
**Modified:**	**No**

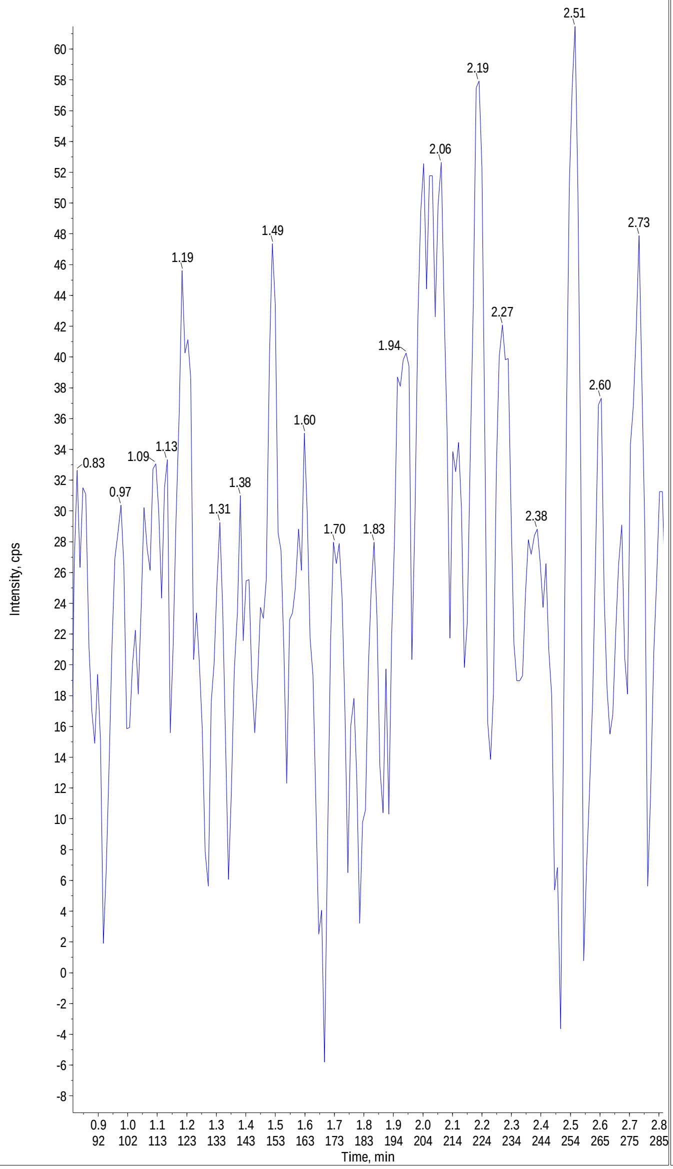

Sample Name: "KB5"   Sample ID: ""   File: "20220801-1.wiff"
Peak Name: "DXP(IS)"   Mass(es): "285.500/154.100 Da"
Comment: ""   Annotation: ""

**Sample Index:**	**8**
**Sample Type:**	**Unknown**
**Concentration:**	**1.00 ng/mL**
**Calculated Conc:**	**N/A**
**Acq. Date:**	**8/1/2022**
**Acq. Time:**	**11:53:05 AM**
**Modified:**	**No**

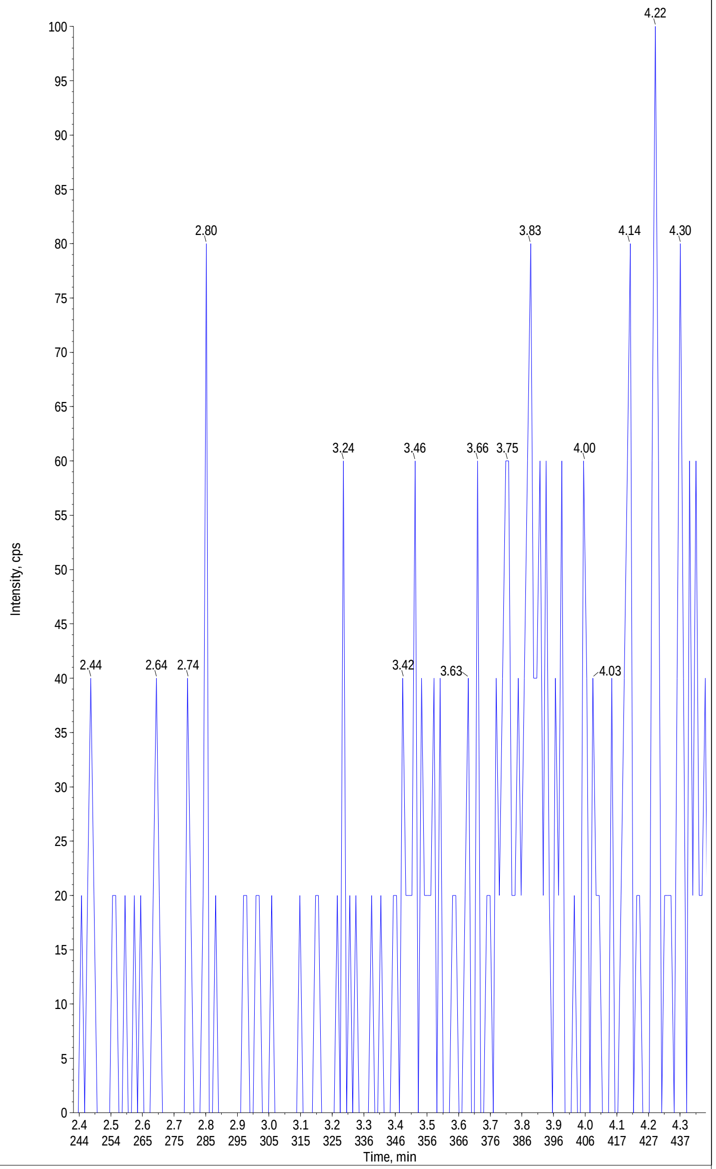

Sample Name: "KB6" Sample ID: "" File: "20220801-1.wiff"
Peak Name: "OHMPT" Mass(es): "235.200/150.200 Da"
Comment: "" Annotation: ""

Sample Index: 9
Sample Type: Unknown
Concentration: N/A
Calculated Conc: No Intercept
Acq. Date: 8/1/2022
Acq. Time: 11:58:04 AM

Modified: No

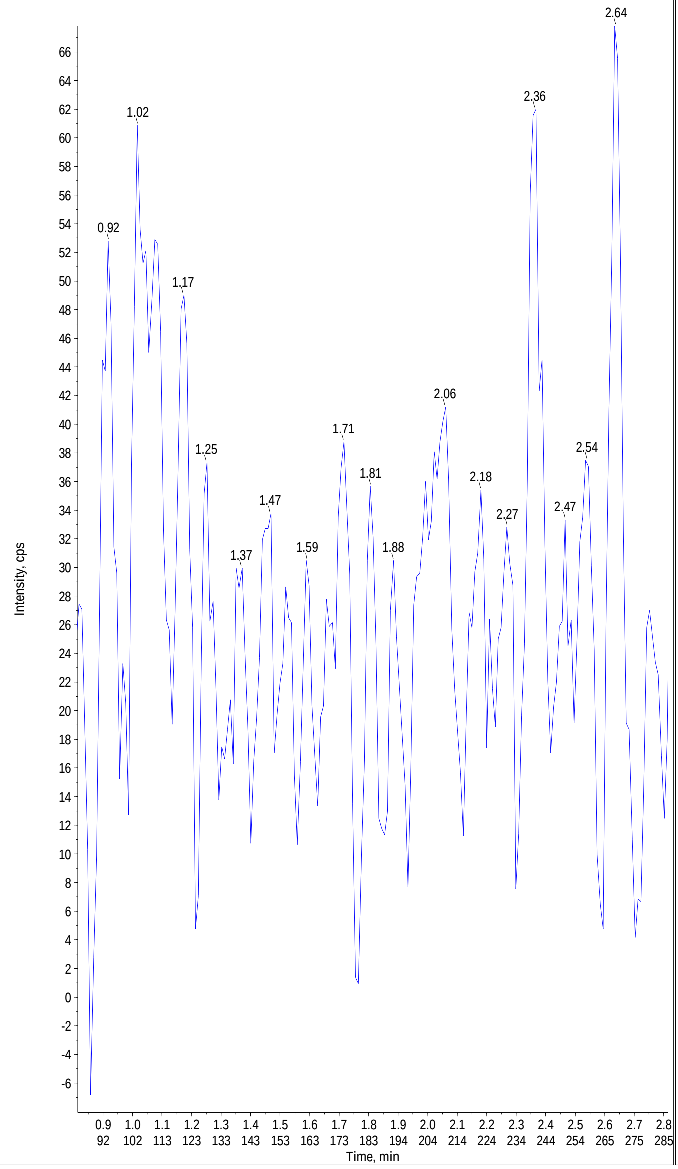

Sample Name: "KB6" Sample ID: "" File: "20220801-1.wiff"
Peak Name: "DXP(IS)" Mass(es): "285.500/154.100 Da"
Comment: "" Annotation: ""

Sample Index: 9
Sample Type: Unknown
Concentration: 1.00 ng/mL
Calculated Conc: N/A
Acq. Date: 8/1/2022
Acq. Time: 11:58:04 AM

Modified: No

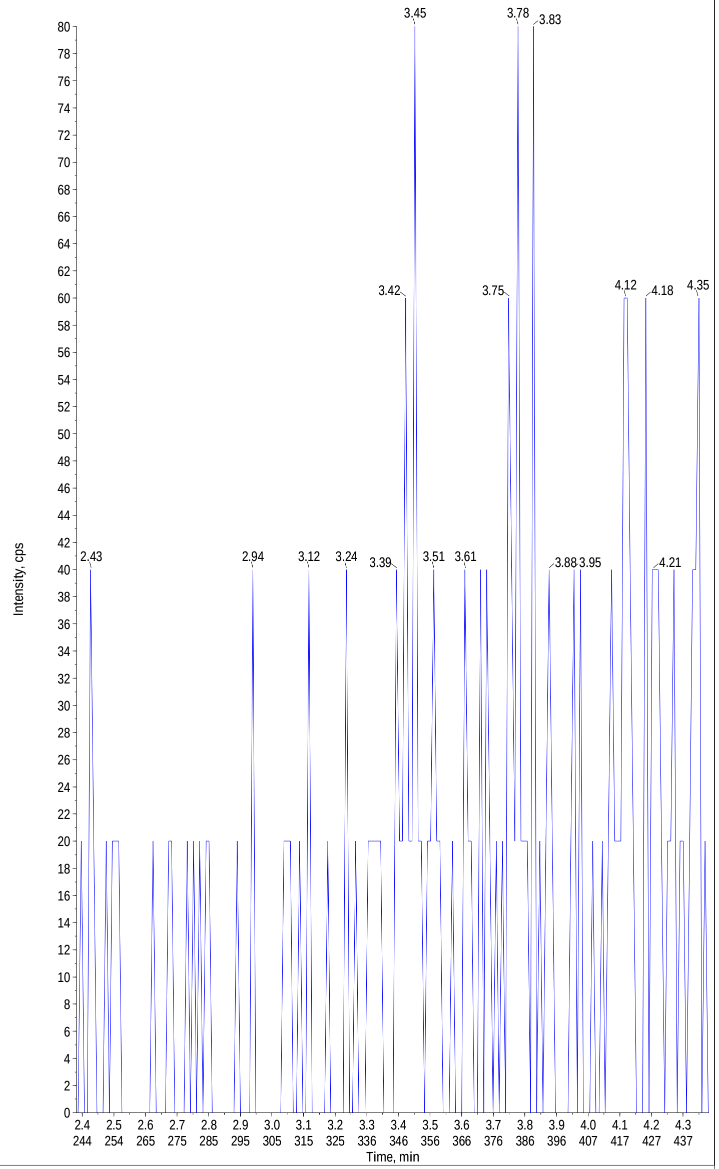

Sample Name: "KB+IS1"   Sample ID: ""   File: "20220801-1.wiff"
Peak Name: "OHMPT"   Mass(es): "235.200/150.200 Da"
Comment: ""   Annotation: ""

```
Sample Index:       10
Sample Type:      Unknown
Concentration:       N/A
Calculated Conc:     0.00    ng/mL
Acq. Date:        8/1/2022
Acq. Time:        12:03:04 PM

Modified:            No
```

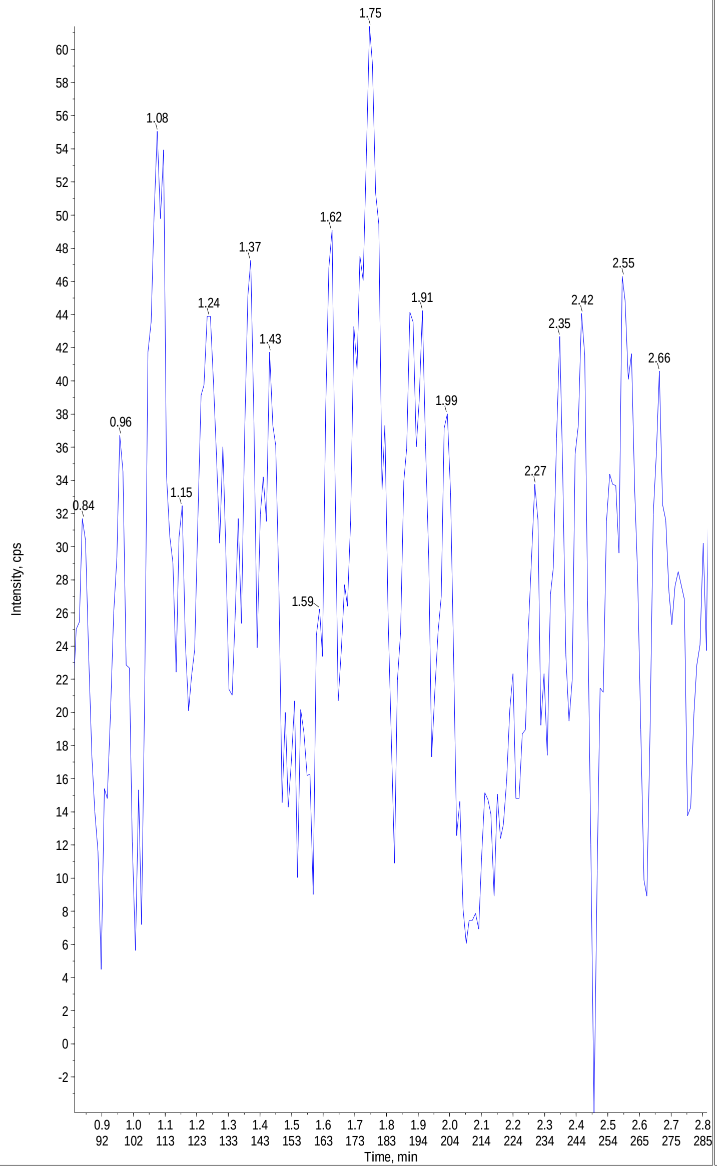

Sample Name: "KB+IS1"   Sample ID: ""   File: "20220801-1.wiff"
Peak Name: "DXP(IS)"   Mass(es): "285.500/154.100 Da"
Comment: ""   Annotation: ""

```
Sample Index:       10
Sample Type:      Unknown
Concentration:       1.00    ng/mL
Calculated Conc:     N/A
Acq. Date:        8/1/2022
Acq. Time:        12:03:04 PM

Modified:            No
Proc. Algorithm: Specify Parameters - MQ III
Noise Percentage:     50
Base. Sub. Window:    1.00   min
Peak-Split. Factor:   2
Report Largest Peak:   Yes
Min. Peak Height:     500.00   cps
Min. Peak Width:       0.00    sec
Smoothing Width:       0       points
RT Window:          30.0    sec
Expected RT:         3.38   min
Use Relative RT:     No

Int. Type:        Base To Base
Retention Time:      3.37   min
Area:          1.68e+006   counts
Height:          2.70e+005   cps
Start Time:          3.25   min
End Time:            4.29   min
```

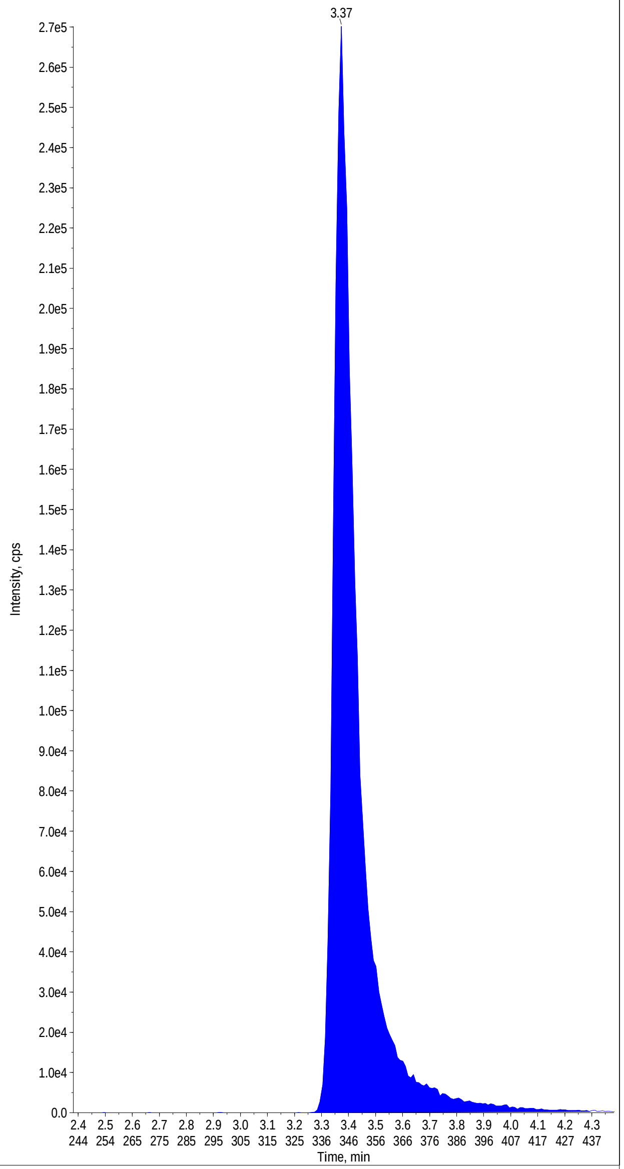

Sample Name: "KB+IS2" Sample ID: "" File: "20220801-1.wiff"
Peak Name: "OHMPT" Mass(es): "235.200/150.200 Da"
Comment: "" Annotation: ""

Sample Index: 11
Sample Type: Unknown
Concentration: N/A
Calculated Conc: 0.00 ng/mL
Acq. Date: 8/1/2022
Acq. Time: 12:08:03 PM

Modified: No

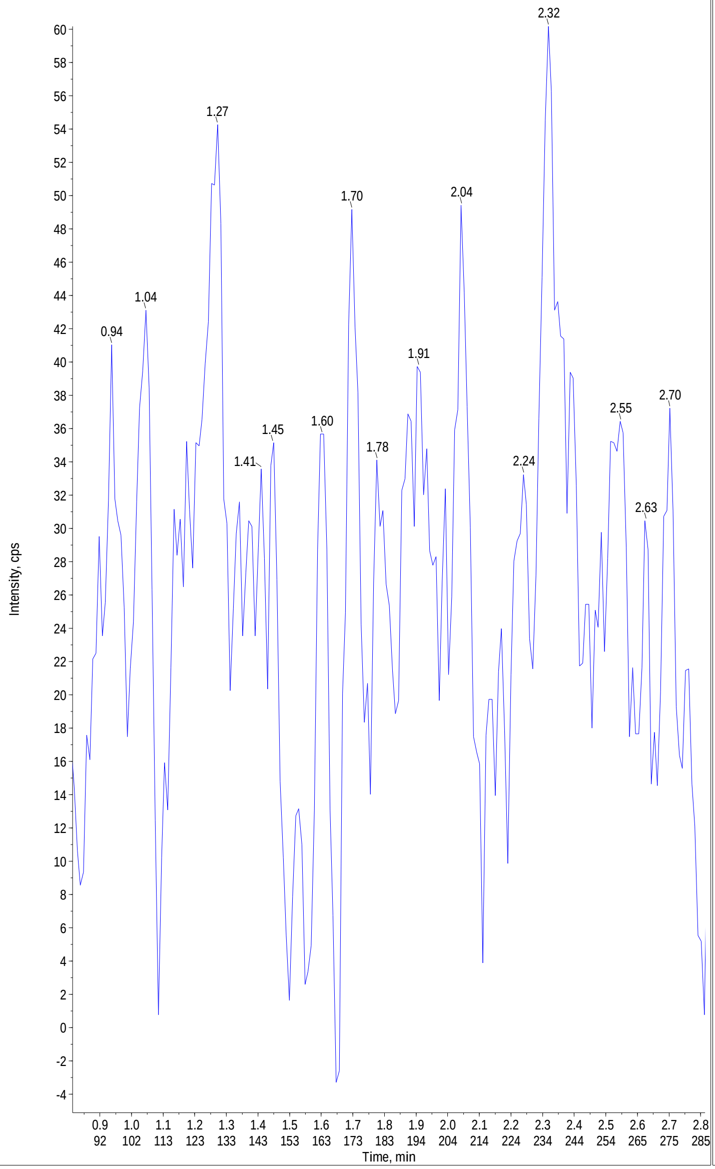

Sample Name: "KB+IS2" Sample ID: "" File: "20220801-1.wiff"
Peak Name: "DXP(IS)" Mass(es): "285.500/154.100 Da"
Comment: "" Annotation: ""

Sample Index: 11
Sample Type: Unknown
Concentration: 1.00 ng/mL
Calculated Conc: N/A
Acq. Date: 8/1/2022
Acq. Time: 12:08:03 PM

Modified: No
Proc. Algorithm: Specify Parameters - MQ III
Noise Percentage: 50
Base. Sub. Window: 1.00 min
Peak-Split. Factor: 2
Report Largest Peak: Yes
Min. Peak Height: 500.00 cps
Min. Peak Width: 0.00 sec
Smoothing Width: 0 points
RT Window: 30.0 sec
Expected RT: 3.38 min
Use Relative RT: No

Int. Type: Valley
Retention Time: 3.39 min
Area: 1.41e+006 counts
Height: 2.28e+005 cps
Start Time: 3.27 min
End Time: 4.13 min

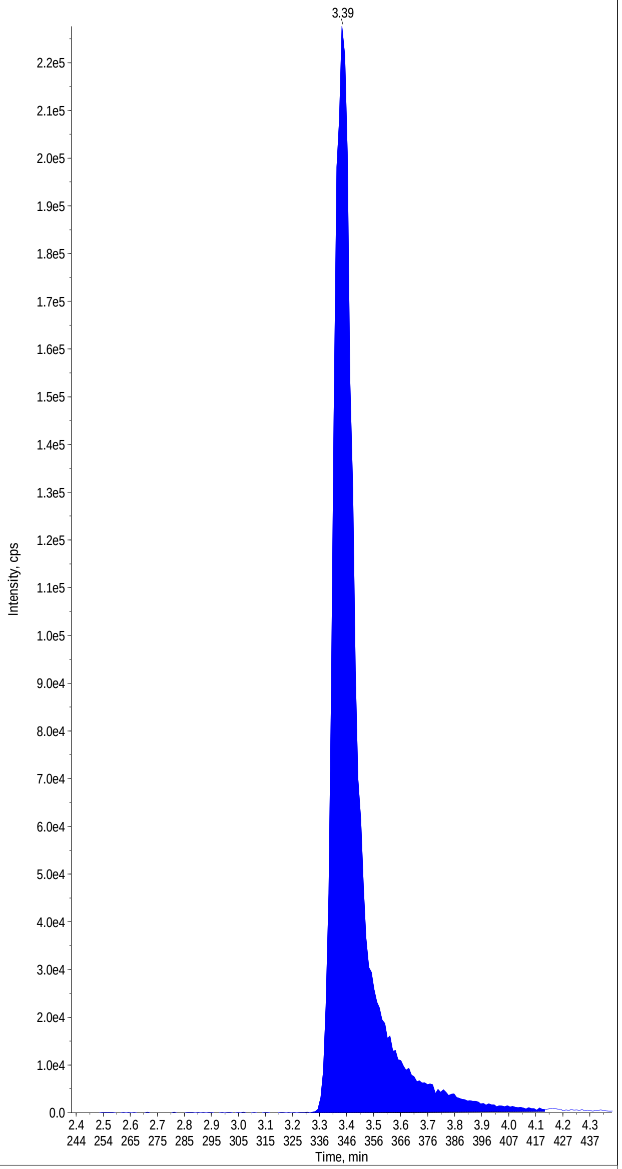

Sample Name: "KB+IS3" Sample ID: "" File: "20220801-1.wiff"
Peak Name: "OHMPT" Mass(es): "235.200/150.200 Da"
Comment: "" Annotation: ""

Sample Index:	12
Sample Type:	Unknown
Concentration:	N/A
Calculated Conc:	0.00 ng/mL
Acq. Date:	8/1/2022
Acq. Time:	12:13:08 PM
Modified:	No

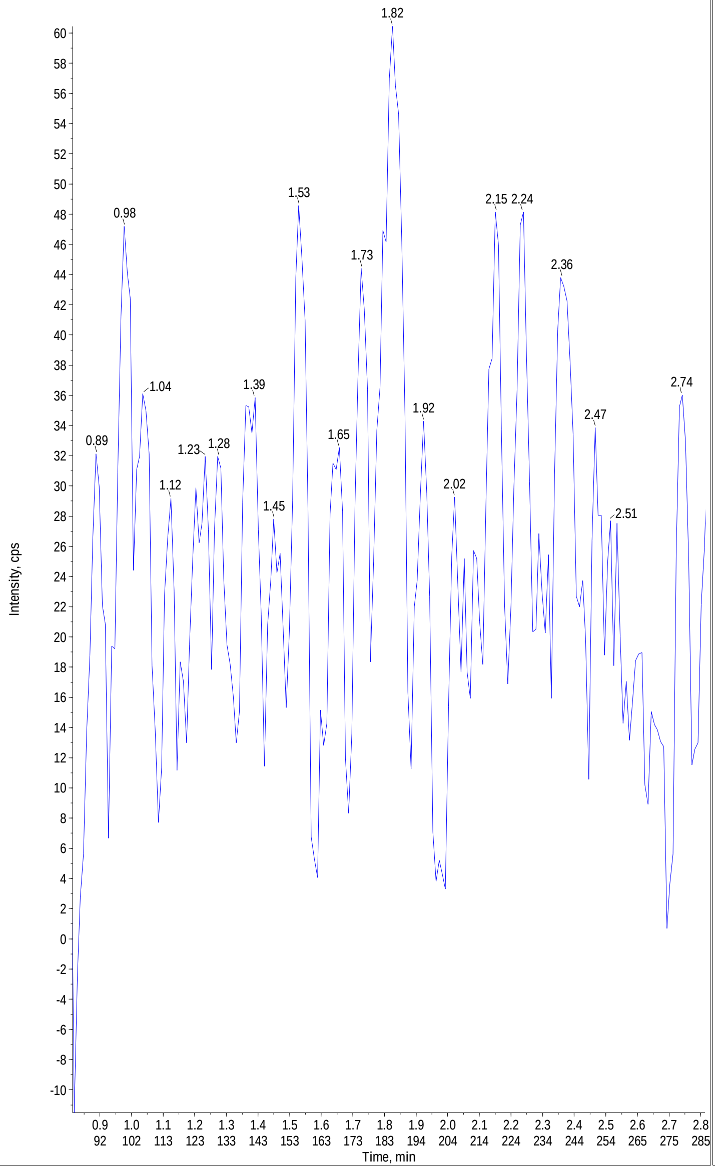

Sample Name: "KB+IS3" Sample ID: "" File: "20220801-1.wiff"
Peak Name: "DXP(IS)" Mass(es): "285.500/154.100 Da"
Comment: "" Annotation: ""

Sample Index:	12
Sample Type:	Unknown
Concentration:	1.00 ng/mL
Calculated Conc:	N/A
Acq. Date:	8/1/2022
Acq. Time:	12:13:08 PM
Modified:	No
Proc. Algorithm:	Specify Parameters - MQ III
Noise Percentage:	50
Base. Sub. Window:	1.00 min
Peak-Split. Factor:	2
Report Largest Peak:	Yes
Min. Peak Height:	500.00 cps
Min. Peak Width:	0.00 sec
Smoothing Width:	0 points
RT Window:	30.0 sec
Expected RT:	3.38 min
Use Relative RT:	No
Int. Type:	Valley
Retention Time:	3.38 min
Area:	1.41e+006 counts
Height:	2.25e+005 cps
Start Time:	3.25 min
End Time:	4.26 min

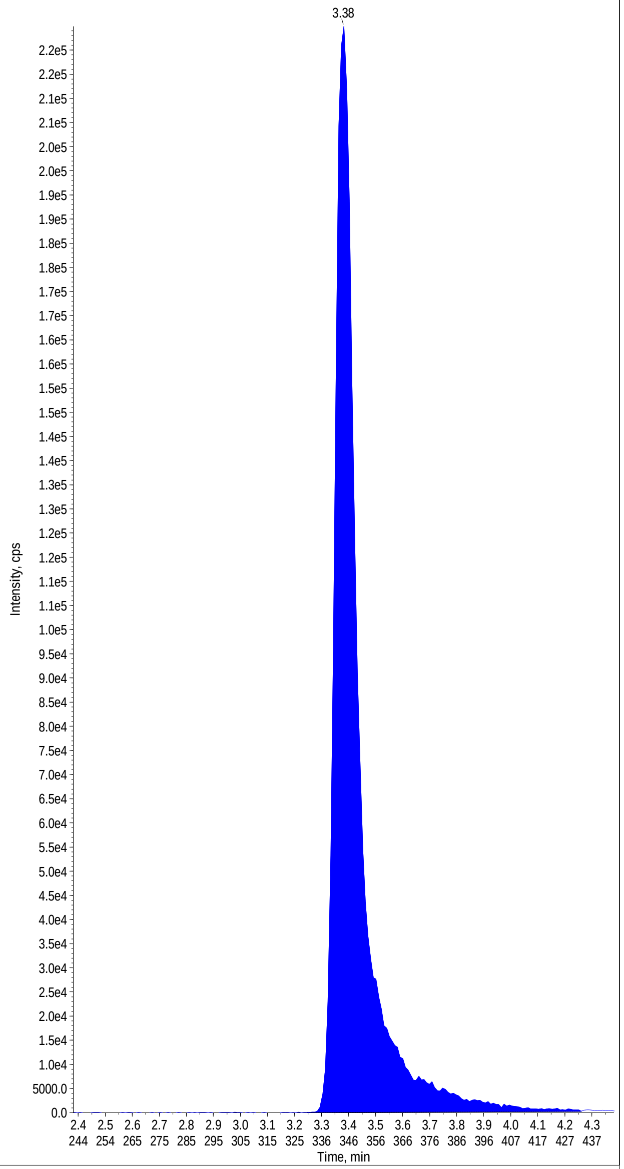

Sample Name: "KB+IS4"   Sample ID: ""   File: "20220801-1.wiff"
Peak Name: "OHMPT"   Mass(es): "235.200/150.200 Da"
Comment: ""   Annotation: ""

Sample Index:	13
Sample Type:	Unknown
Concentration:	N/A
Calculated Conc:	0.00 ng/mL
Acq. Date:	8/1/2022
Acq. Time:	12:18:06 PM
Modified:	No

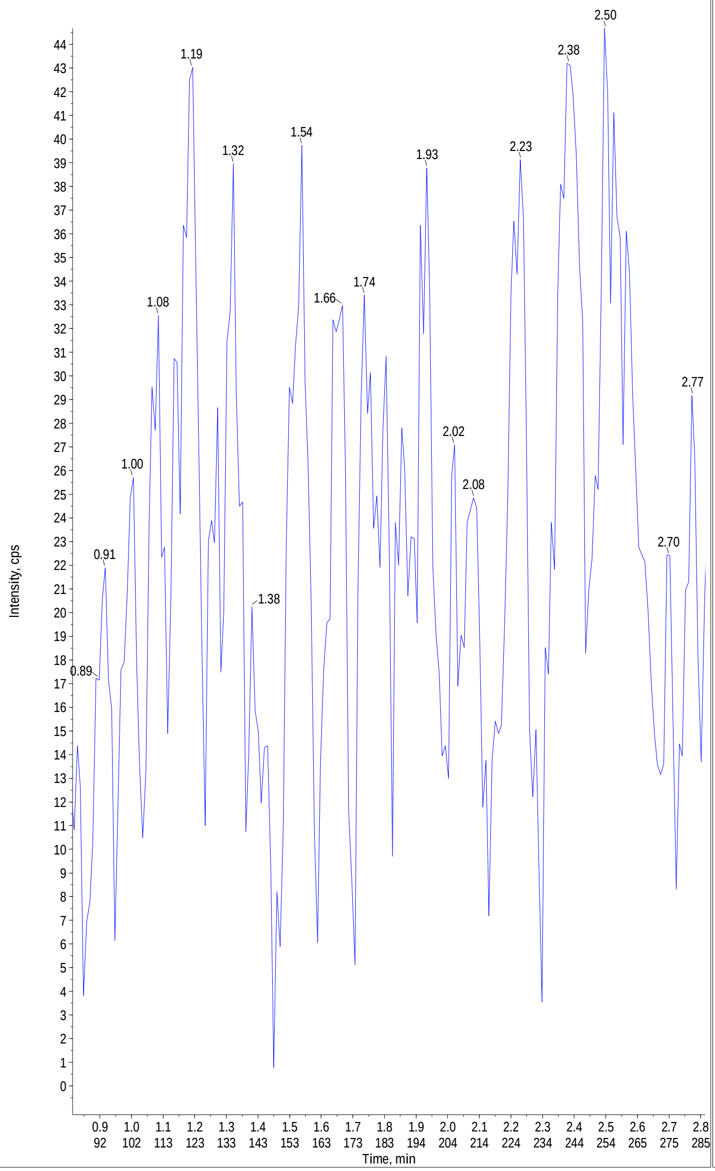

Sample Name: "KB+IS4"   Sample ID: ""   File: "20220801-1.wiff"
Peak Name: "DXP(IS)"   Mass(es): "285.500/154.100 Da"
Comment: ""   Annotation: ""

Sample Index:	13
Sample Type:	Unknown
Concentration:	1.00 ng/mL
Calculated Conc:	N/A
Acq. Date:	8/1/2022
Acq. Time:	12:18:06 PM
Modified:	No
Proc. Algorithm:	Specify Parameters - MQ III
Noise Percentage:	50
Base. Sub. Window:	1.00 min
Peak-Split. Factor:	2
Report Largest Peak:	Yes
Min. Peak Height:	500.00 cps
Min. Peak Width:	0.00 sec
Smoothing Width:	0 points
RT Window:	30.0 sec
Expected RT:	3.38 min
Use Relative RT:	No
Int. Type:	Valley
Retention Time:	3.38 min
Area:	1.39e+006 counts
Height:	2.22e+005 cps
Start Time:	3.26 min
End Time:	4.06 min

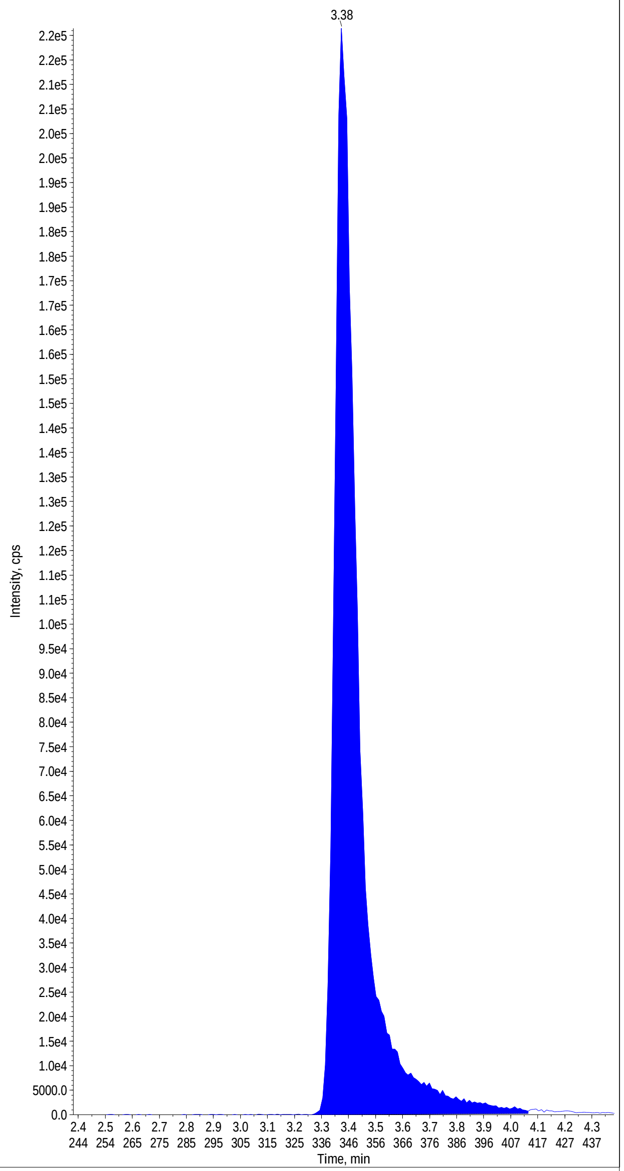

Sample Name: "KB+IS5" Sample ID: "" File: "20220801-1.wiff"
Peak Name: "OHMPT" Mass(es): "235.200/150.200 Da"
Comment: "" Annotation: ""

```
Sample Index:      14
Sample Type:      Unknown
Concentration:      N/A
Calculated Conc:     0.00    ng/mL
Acq. Date:       8/1/2022
Acq. Time:       12:23:11 PM

Modified:          No
```

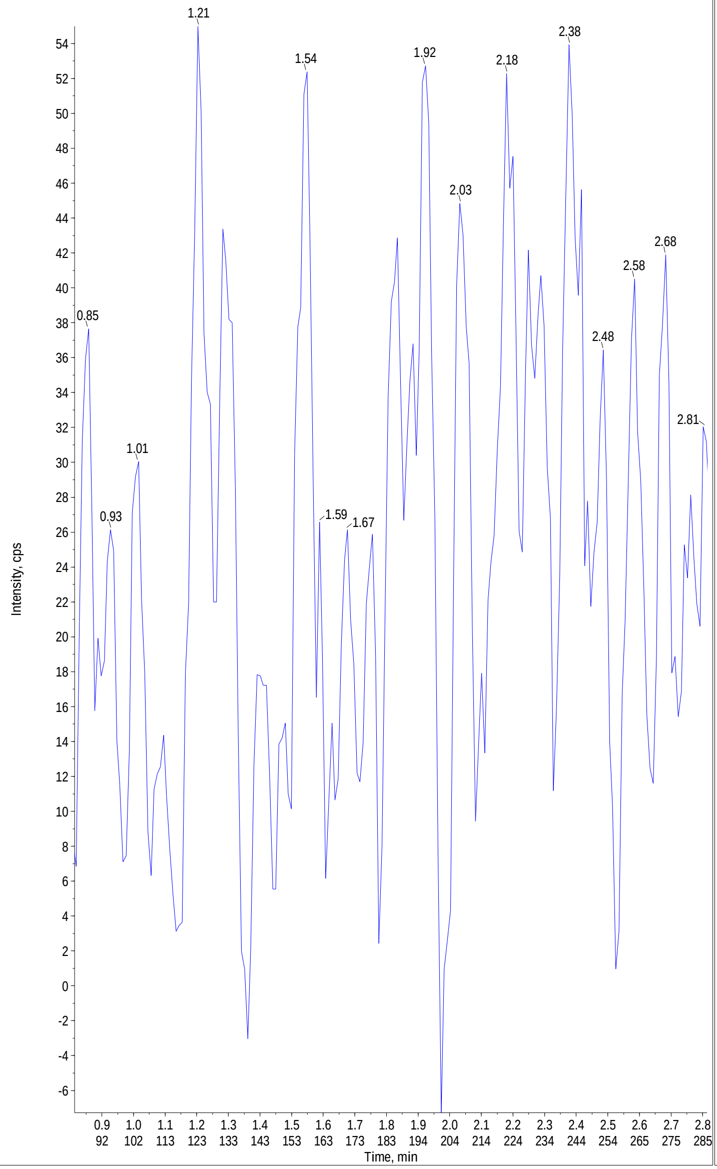

Sample Name: "KB+IS5" Sample ID: "" File: "20220801-1.wiff"
Peak Name: "DXP(IS)" Mass(es): "285.500/154.100 Da"
Comment: "" Annotation: ""

```
Sample Index:      14
Sample Type:      Unknown
Concentration:      1.00    ng/mL
Calculated Conc:    N/A
Acq. Date:       8/1/2022
Acq. Time:       12:23:11 PM

Modified:          No
Proc. Algorithm: Specify Parameters - MQ III
Noise Percentage:     50
Base. Sub. Window:    1.00   min
Peak-Split. Factor:   2
Report Largest Peak:   Yes
Min. Peak Height:    500.00   cps
Min. Peak Width:      0.00   sec
Smoothing Width:      0      points
RT Window:        30.0    sec
Expected RT:       3.38   min
Use Relative RT:    No

Int. Type:        Valley
Retention Time:     3.37   min
Area:          1.35e+006   counts
Height:          2.30e+005   cps
Start Time:        3.27   min
End Time:         3.96   min
```

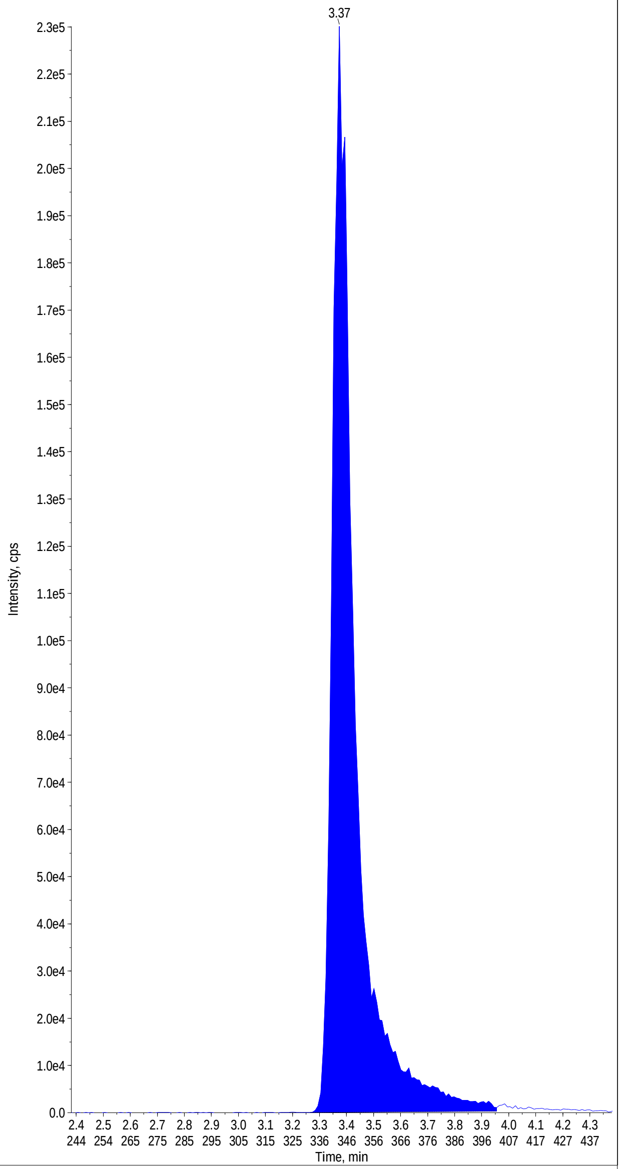

Sample Name: "KB+IS6" Sample ID: "" File: "20220801-1.wiff"
Peak Name: "OHMPT" Mass(es): "235.200/150.200 Da"
Comment: "" Annotation: ""

```
Sample Index:      15
Sample Type:       Unknown
Concentration:      N/A
Calculated Conc:    0.00    ng/mL
Acq. Date:         8/1/2022
Acq. Time:         12:28:13 PM

Modified:           No
```

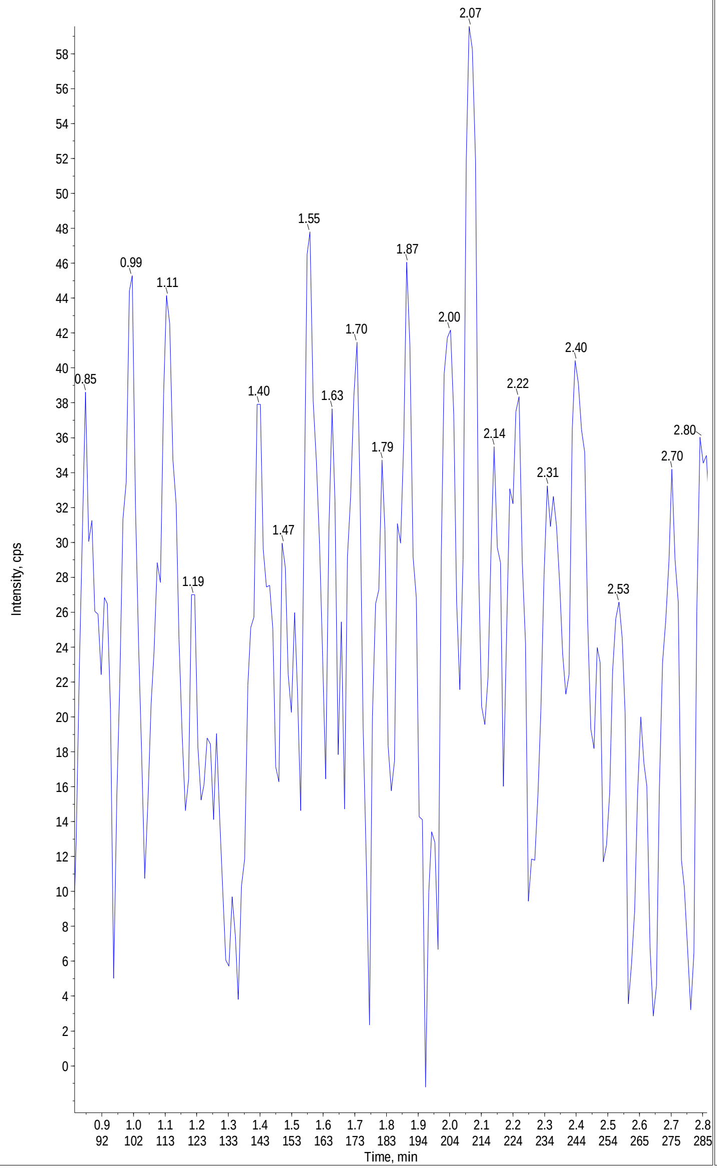

Sample Name: "KB+IS6" Sample ID: "" File: "20220801-1.wiff"
Peak Name: "DXP(IS)" Mass(es): "285.500/154.100 Da"
Comment: "" Annotation: ""

```
Sample Index:      15
Sample Type:       Unknown
Concentration:      1.00    ng/mL
Calculated Conc:    N/A
Acq. Date:         8/1/2022
Acq. Time:         12:28:13 PM

Modified:           No
Proc. Algorithm: Specify Parameters - MQ III
Noise Percentage:     50
Base. Sub. Window:     1.00   min
Peak-Split. Factor:   2
Report Largest Peak:   Yes
Min. Peak Height:     500.00   cps
Min. Peak Width:        0.00   sec
Smoothing Width:        0       points
RT Window:          30.0    sec
Expected RT:         3.38   min
Use Relative RT:     No

Int. Type:        Base To Base
Retention Time:      3.39   min
Area:          1.36e+006   counts
Height:          2.04e+005   cps
Start Time:          3.27   min
End Time:            4.28   min
```

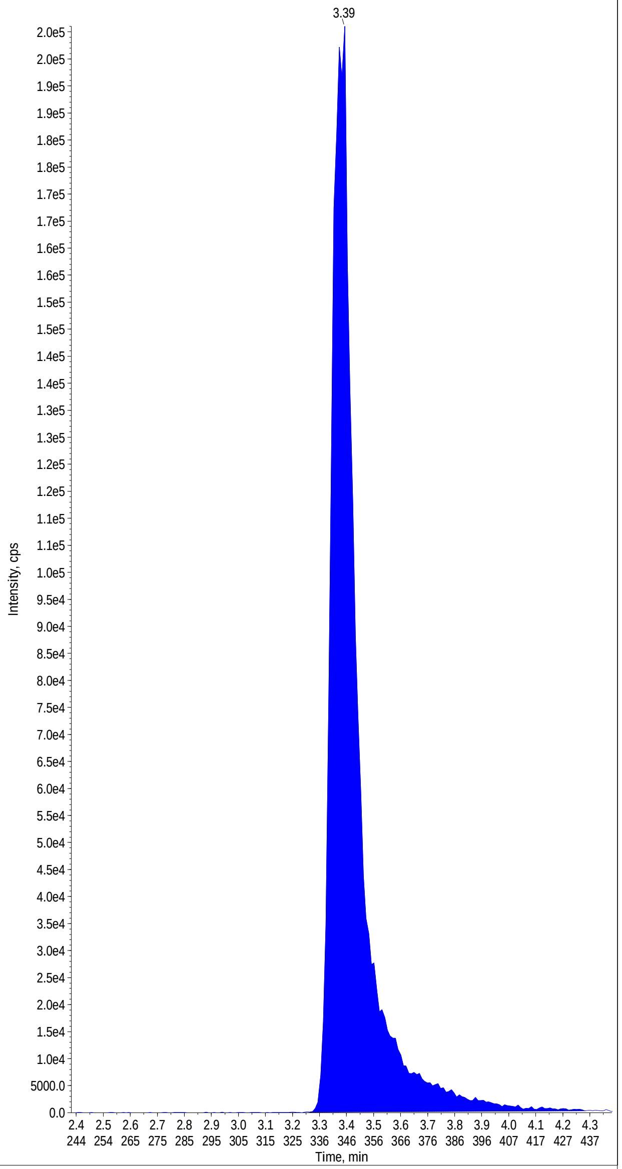

Sample Name: "SPIKE SAMPLE1" Sample ID: "" File: "20220801-1.wiff"
Peak Name: "OHMPT" Mass(es): "235.200/150.200 Da"
Comment: "" Annotation: ""

Sample Index: 43
Sample Type: Unknown
Concentration: N/A
Calculated Conc: No Intercept
Acq. Date: 8/1/2022
Acq. Time: 6:55:36 PM

Modified: No
Proc. Algorithm: Specify Parameters - MQ III
Noise Percentage: 50
Base. Sub. Window: 1.00 min
Peak-Split. Factor: 2
Report Largest Peak: Yes
Min. Peak Height: 300.00 cps
Min. Peak Width: 0.00 sec
Smoothing Width: 9 points
RT Window: 30.0 sec
Expected RT: 1.81 min
Use Relative RT: No

Int. Type: Valley
Retention Time: 1.83 min
Area: 5.21e+003 counts
Height: 7.13e+002 cps
Start Time: 1.74 min
End Time: 2.12 min

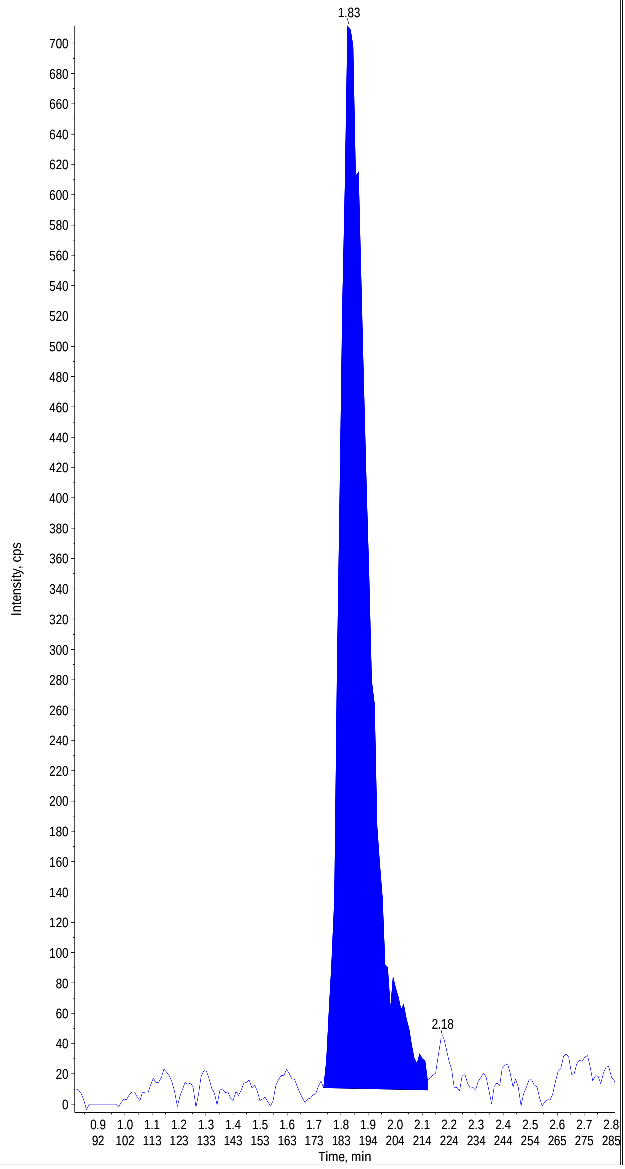

Sample Name: "SPIKE SAMPLE1" Sample ID: "" File: "20220801-1.wiff"
Peak Name: "DXP(IS)" Mass(es): "285.500/154.100 Da"
Comment: "" Annotation: ""

Sample Index: 43
Sample Type: Unknown
Concentration: 1.00 ng/mL
Calculated Conc: N/A
Acq. Date: 8/1/2022
Acq. Time: 6:55:36 PM

Modified: No

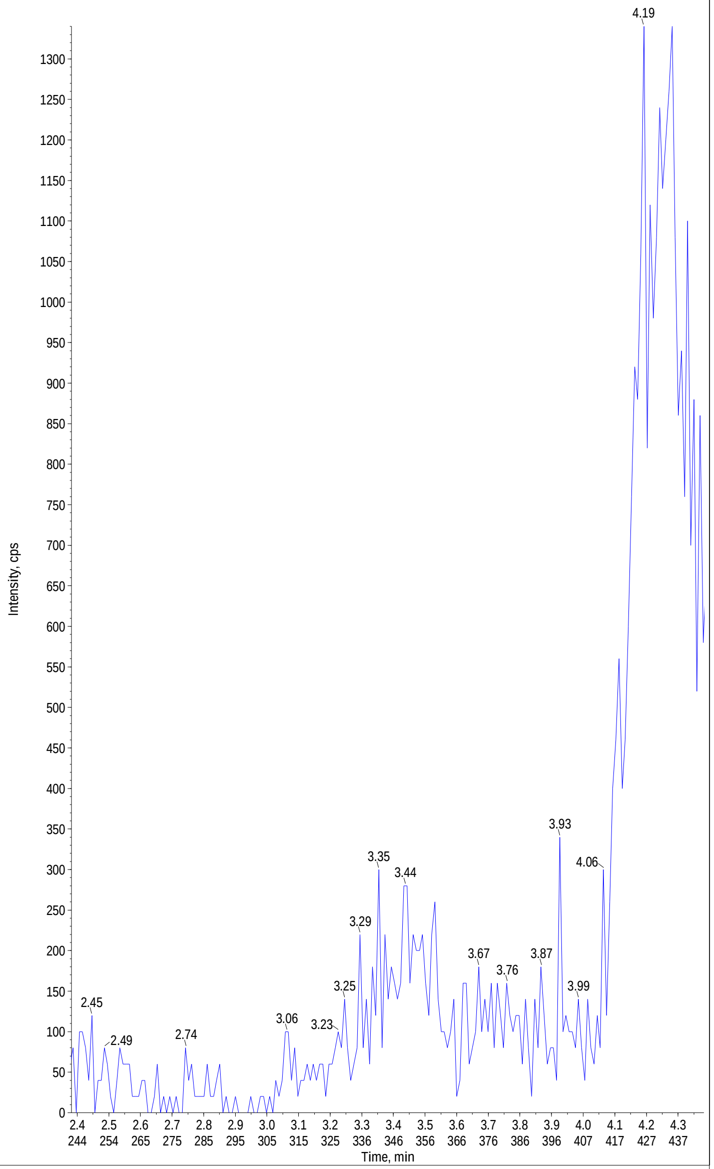

Sample Name: "SPIKE SAMPLE2"   Sample ID: ""   File: "20220801-1.wiff"
Peak Name: "OHMPT"   Mass(es): "235.200/150.200 Da"
Comment: ""   Annotation: ""

```
Sample Index:       44
Sample Type:       Unknown
Concentration:       N/A
Calculated Conc: No Intercept
Acq. Date:        8/1/2022
Acq. Time:        7:00:38 PM

Modified:            No
Proc. Algorithm: Specify Parameters - MQ III
Noise Percentage:     50
Base. Sub. Window:     1.00   min
Peak-Split. Factor:   2
Report Largest Peak:   Yes
Min. Peak Height:    300.00   cps
Min. Peak Width:       0.00   sec
Smoothing Width:       9       points
RT Window:          30.0    sec
Expected RT:         1.81    min
Use Relative RT:     No

Int. Type:        Base To Base
Retention Time:      1.83    min
Area:          6.00e+003    counts
Height:          8.52e+002   cps
Start Time:          1.73    min
End Time:            2.10    min
```

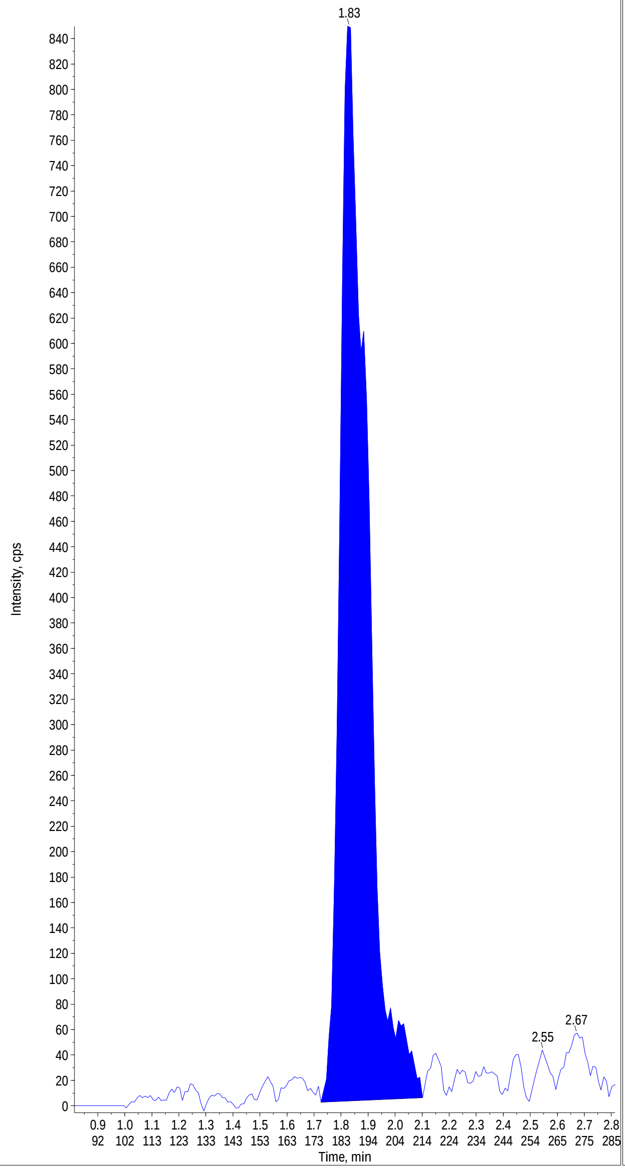

Sample Name: "SPIKE SAMPLE2"   Sample ID: ""   File: "20220801-1.wiff"
Peak Name: "DXP(IS)"   Mass(es): "285.500/154.100 Da"
Comment: ""   Annotation: ""

```
Sample Index:       44
Sample Type:       Unknown
Concentration:       1.00    ng/mL
Calculated Conc:     N/A
Acq. Date:        8/1/2022
Acq. Time:        7:00:38 PM

Modified:            No
```

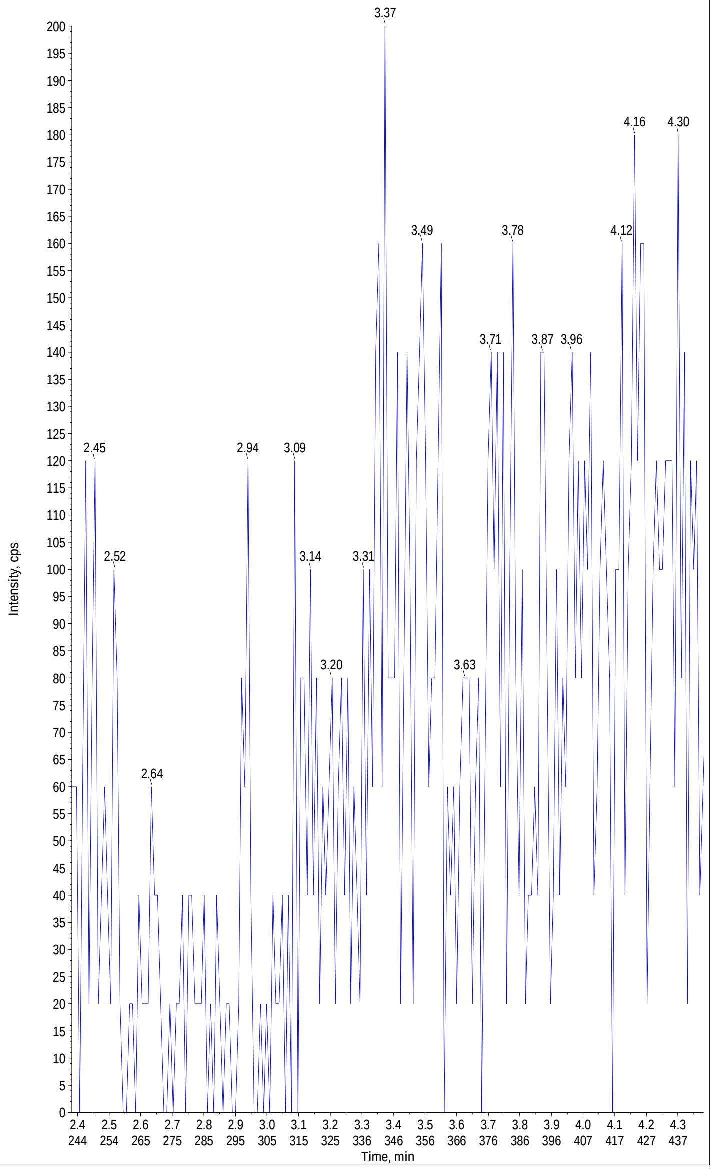

Sample Name: "SPIKE SAMPLE3" Sample ID: "" File: "20220801-1.wiff"
Peak Name: "OHMPT" Mass(es): "235.200/150.200 Da"
Comment: "" Annotation: ""

```
Sample Index:      45
Sample Type:      Unknown
Concentration:      N/A
Calculated Conc: No Intercept
Acq. Date:        8/1/2022
Acq. Time:        7:05:39 PM

Modified:            No
Proc. Algorithm: Specify Parameters - MQ III
Noise Percentage:     50
Base. Sub. Window:    1.00   min
Peak-Split. Factor:   2
Report Largest Peak:   Yes
Min. Peak Height:    300.00   cps
Min. Peak Width:       0.00   sec
Smoothing Width:       9       points
RT Window:          30.0    sec
Expected RT:         1.81    min
Use Relative RT:     No

Int. Type:        Base To Base
Retention Time:     1.84    min
Area:          5.80e+003   counts
Height:          9.27e+002  cps
Start Time:         1.76    min
End Time:           2.16    min
```

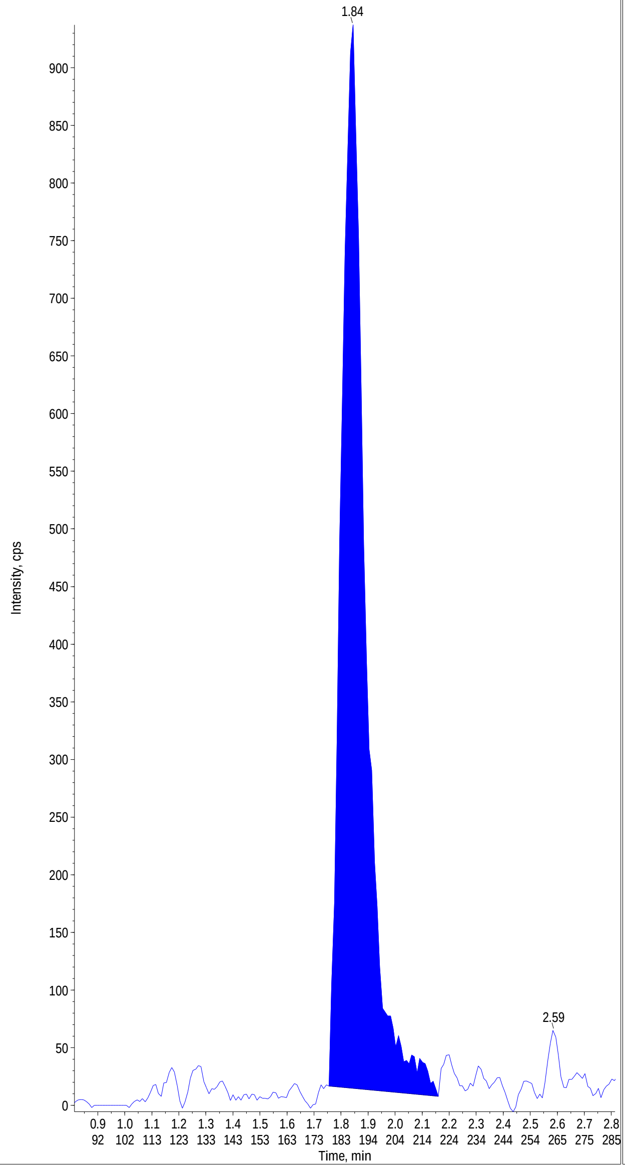

Sample Name: "SPIKE SAMPLE3" Sample ID: "" File: "20220801-1.wiff"
Peak Name: "DXP(IS)" Mass(es): "285.500/154.100 Da"
Comment: "" Annotation: ""

```
Sample Index:      45
Sample Type:      Unknown
Concentration:      1.00    ng/mL
Calculated Conc:    N/A
Acq. Date:        8/1/2022
Acq. Time:        7:05:39 PM

Modified:            No
```

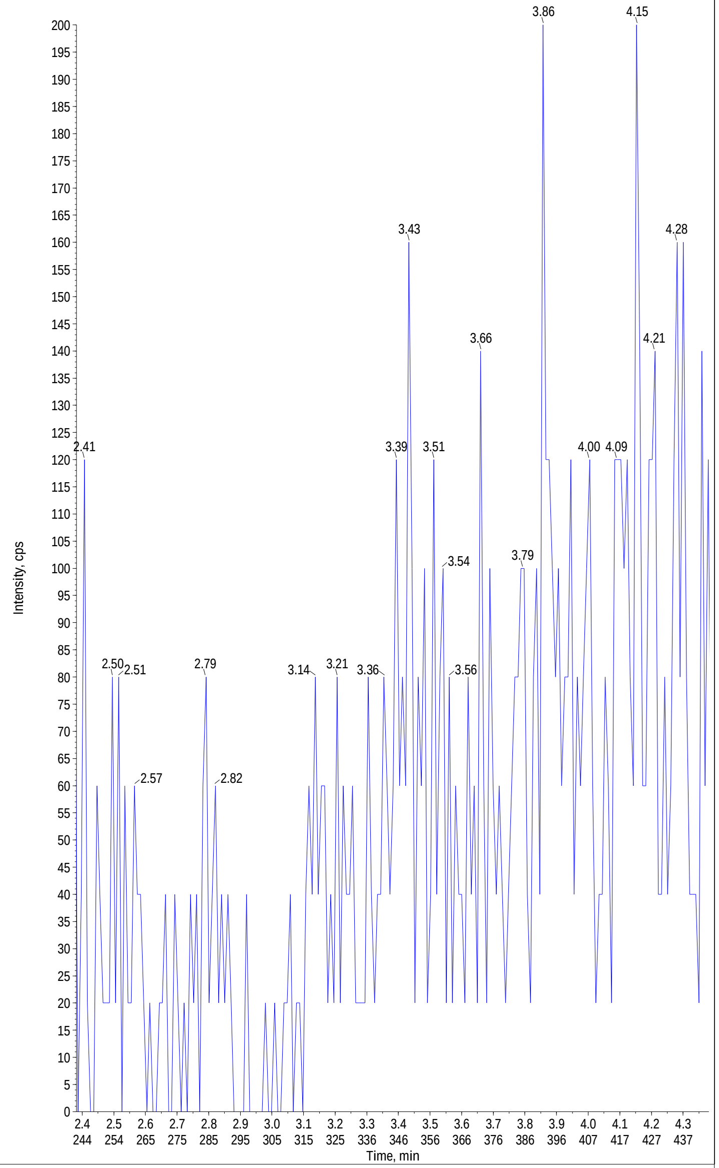

Sample Name: "SPIKE SAMPLE4" Sample ID: "" File: "20220801-1.wiff"
Peak Name: "OHMPT" Mass(es): "235.200/150.200 Da"
Comment: "" Annotation: ""

```
Sample Index:      46
Sample Type:       Unknown
Concentration:     N/A
Calculated Conc: No Intercept
Acq. Date:         8/1/2022
Acq. Time:         7:10:44 PM

Modified:          No
Proc. Algorithm: Specify Parameters - MQ III
Noise Percentage:     50
Base. Sub. Window:    1.00   min
Peak-Split. Factor:   2
Report Largest Peak:  Yes
Min. Peak Height:    300.00   cps
Min. Peak Width:       0.00   sec
Smoothing Width:       9       points
RT Window:           30.0    sec
Expected RT:          1.81   min
Use Relative RT:      No

Int. Type:         Base To Base
Retention Time:       1.84   min
Area:            5.76e+003   counts
Height:          8.39e+002   cps
Start Time:           1.75   min
End Time:             2.07   min
```

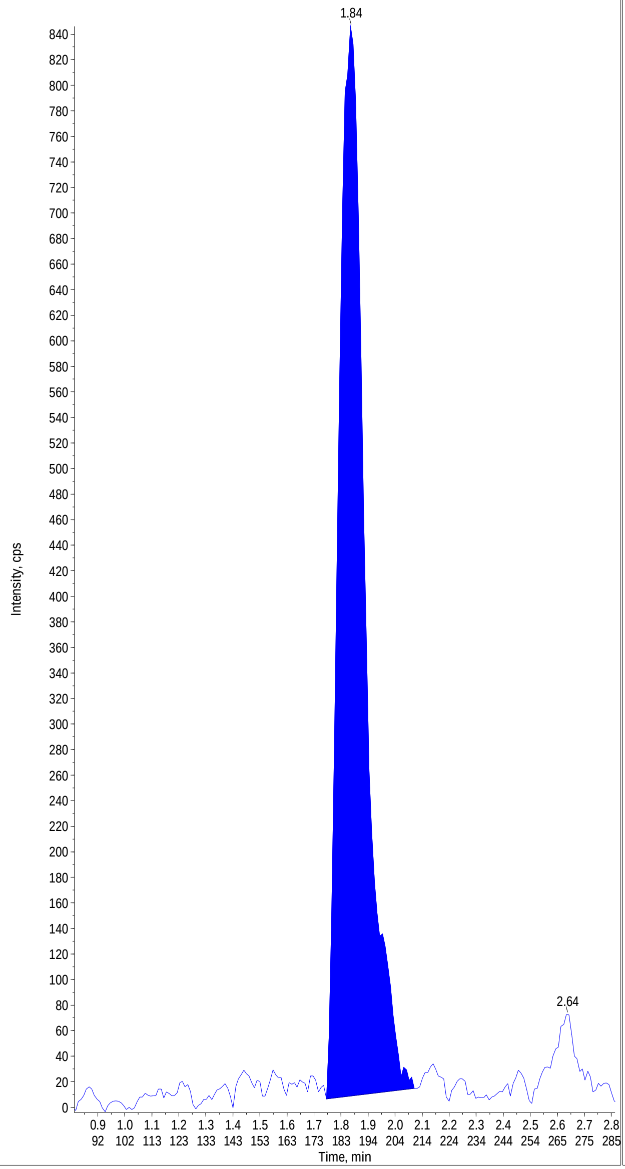

Sample Name: "SPIKE SAMPLE4" Sample ID: "" File: "20220801-1.wiff"
Peak Name: "DXP(IS)" Mass(es): "285.500/154.100 Da"
Comment: "" Annotation: ""

```
Sample Index:      46
Sample Type:       Unknown
Concentration:     1.00    ng/mL
Calculated Conc:   N/A
Acq. Date:         8/1/2022
Acq. Time:         7:10:44 PM

Modified:          No
```

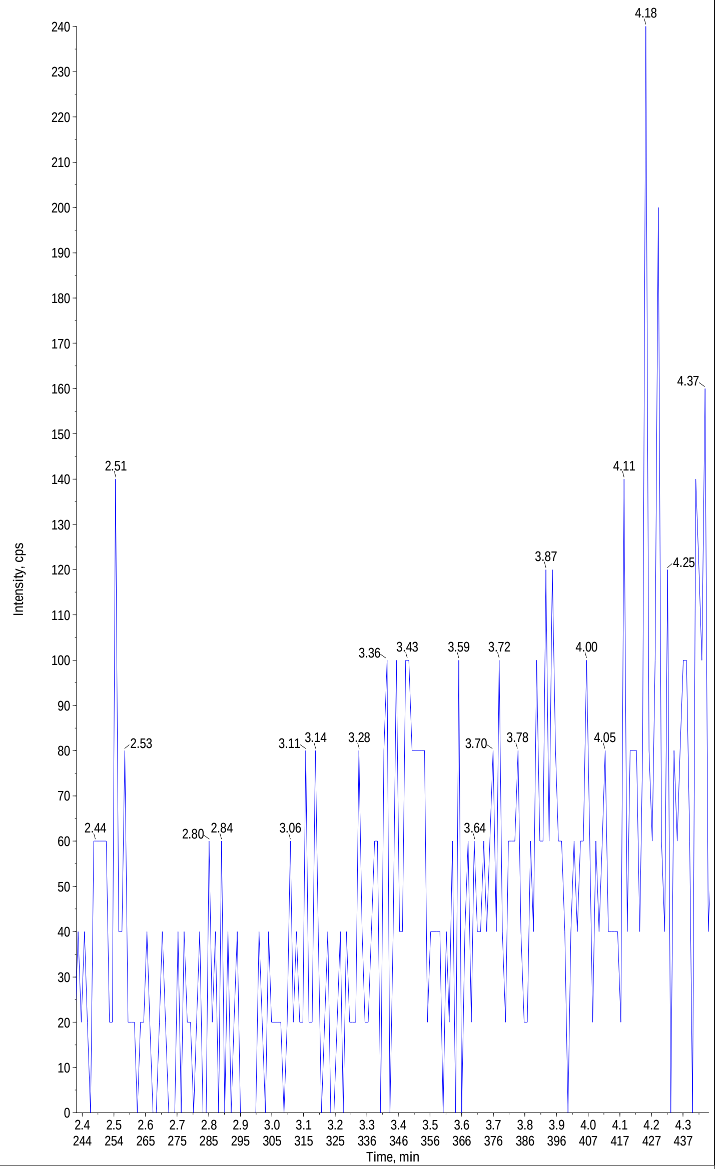

Sample Name: "SPIKE SAMPLE5" Sample ID: "" File: "20220801-1.wiff"
Peak Name: "OHMPT" Mass(es): "235.200/150.200 Da"
Comment: "" Annotation: ""

Sample Index: 47
Sample Type: Unknown
Concentration: N/A
Calculated Conc: No Intercept
Acq. Date: 8/1/2022
Acq. Time: 7:15:45 PM

Modified: No
Proc. Algorithm: Specify Parameters - MQ III
Noise Percentage: 50
Base. Sub. Window: 1.00 min
Peak-Split. Factor: 2
Report Largest Peak: Yes
Min. Peak Height: 300.00 cps
Min. Peak Width: 0.00 sec
Smoothing Width: 9 points
RT Window: 30.0 sec
Expected RT: 1.81 min
Use Relative RT: No

Int. Type: Valley
Retention Time: 1.83 min
Area: 6.33e+003 counts
Height: 9.08e+002 cps
Start Time: 1.72 min
End Time: 2.08 min

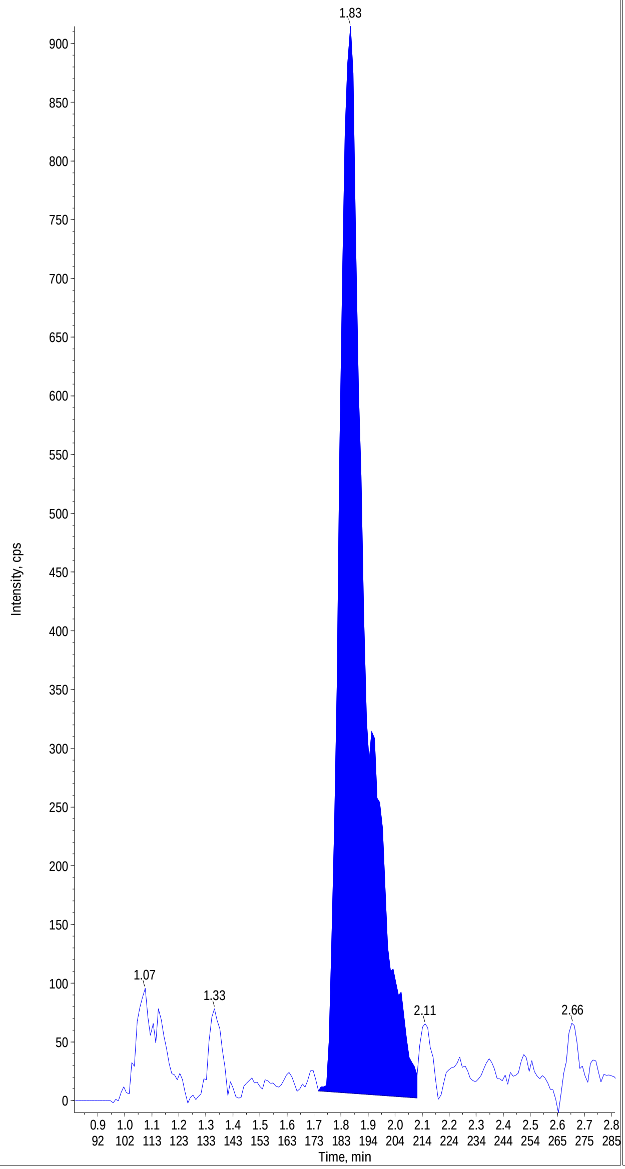

Sample Name: "SPIKE SAMPLE5" Sample ID: "" File: "20220801-1.wiff"
Peak Name: "DXP(IS)" Mass(es): "285.500/154.100 Da"
Comment: "" Annotation: ""

Sample Index: 47
Sample Type: Unknown
Concentration: 1.00 ng/mL
Calculated Conc: N/A
Acq. Date: 8/1/2022
Acq. Time: 7:15:45 PM

Modified: No

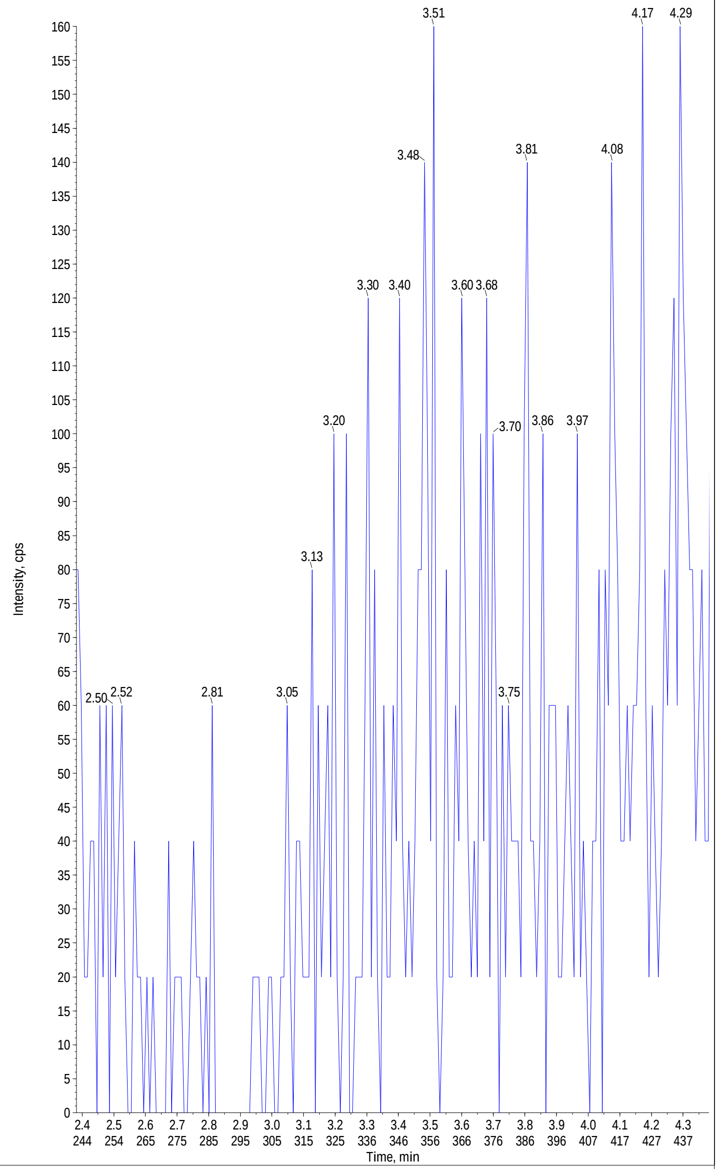

Sample Name: "SPIKE SAMPLE6" Sample ID: "" File: "20220801-1.wiff"
Peak Name: "OHMPT" Mass(es): "235.200/150.200 Da"
Comment: "" Annotation: ""

```
Sample Index:      48
Sample Type:      Unknown
Concentration:      N/A
Calculated Conc: No Intercept
Acq. Date:        8/1/2022
Acq. Time:        7:20:49 PM

Modified:           No
Proc. Algorithm: Specify Parameters - MQ III
Noise Percentage:     50
Base. Sub. Window:    1.00   min
Peak-Split. Factor:   2
Report Largest Peak:   Yes
Min. Peak Height:    300.00   cps
Min. Peak Width:       0.00   sec
Smoothing Width:       9       points
RT Window:          30.0    sec
Expected RT:         1.81    min
Use Relative RT:    No

Int. Type:       Base To Base
Retention Time:     1.83    min
Area:          5.58e+003    counts
Height:          9.58e+002  cps
Start Time:         1.75    min
End Time:           2.06    min
```

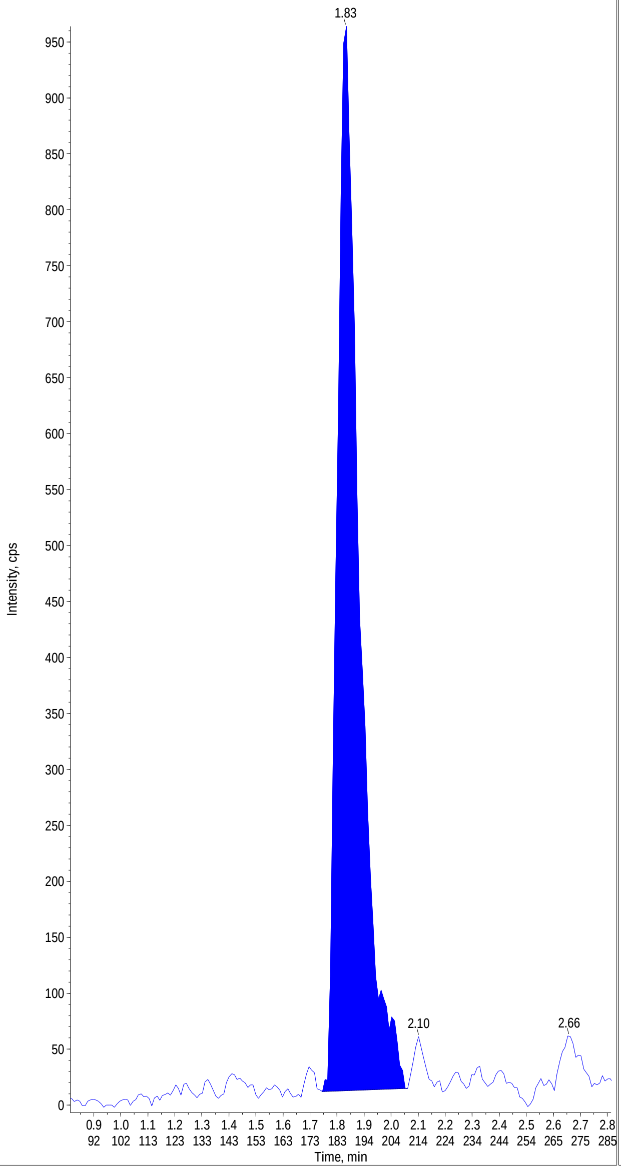

Sample Name: "SPIKE SAMPLE6" Sample ID: "" File: "20220801-1.wiff"
Peak Name: "DXP(IS)" Mass(es): "285.500/154.100 Da"
Comment: "" Annotation: ""

```
Sample Index:      48
Sample Type:      Unknown
Concentration:      1.00    ng/mL
Calculated Conc:    N/A
Acq. Date:        8/1/2022
Acq. Time:        7:20:49 PM

Modified:           No
```

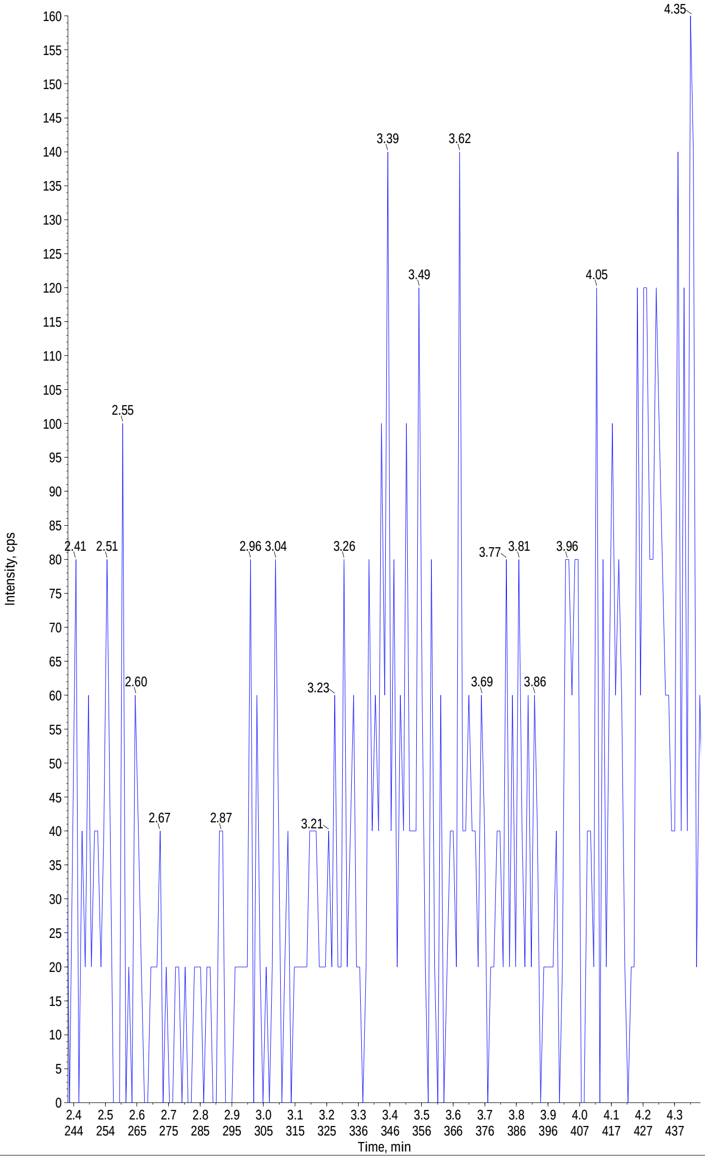

Sample Name: "LLOQ1" Sample ID: "" File: "20220801-1.wiff"
Peak Name: "OHMPT" Mass(es): "235.200/150.200 Da"
Comment: "" Annotation: ""

Sample Index:	36	
Sample Type:	Unknown	
Concentration:	N/A	
Calculated Conc:	0.00	ng/mL
Acq. Date:	8/1/2022	
Acq. Time:	3:38:38 PM	
Modified:	No	
Proc. Algorithm:	Specify Parameters - MQ III	
Noise Percentage:	50	
Base. Sub. Window:	1.00	min
Peak-Split. Factor:	2	
Report Largest Peak:	Yes	
Min. Peak Height:	300.00	cps
Min. Peak Width:	0.00	sec
Smoothing Width:	9	points
RT Window:	30.0	sec
Expected RT:	1.81	min
Use Relative RT:	No	
Int. Type:	Valley	
Retention Time:	1.80	min
Area:	1.92e+004	counts
Height:	2.61e+003	cps
Start Time:	1.67	min
End Time:	1.93	min

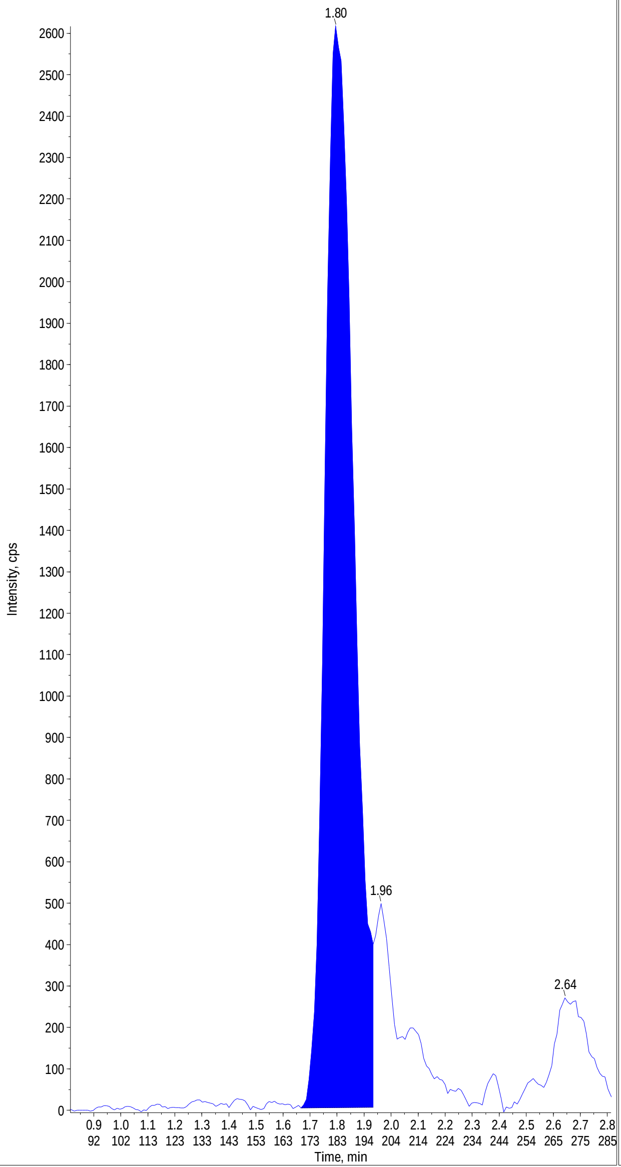

Sample Name: "LLOQ1" Sample ID: "" File: "20220801-1.wiff"
Peak Name: "DXP(IS)" Mass(es): "285.500/154.100 Da"
Comment: "" Annotation: ""

Sample Index:	36	
Sample Type:	Unknown	
Concentration:	1.00	ng/mL
Calculated Conc:	N/A	
Acq. Date:	8/1/2022	
Acq. Time:	3:38:38 PM	
Modified:	No	
Proc. Algorithm:	Specify Parameters - MQ III	
Noise Percentage:	50	
Base. Sub. Window:	1.00	min
Peak-Split. Factor:	2	
Report Largest Peak:	Yes	
Min. Peak Height:	500.00	cps
Min. Peak Width:	0.00	sec
Smoothing Width:	0	points
RT Window:	30.0	sec
Expected RT:	3.38	min
Use Relative RT:	No	
Int. Type:	Base To Base	
Retention Time:	3.38	min
Area:	1.20e+007	counts
Height:	1.90e+006	cps
Start Time:	3.25	min
End Time:	4.32	min

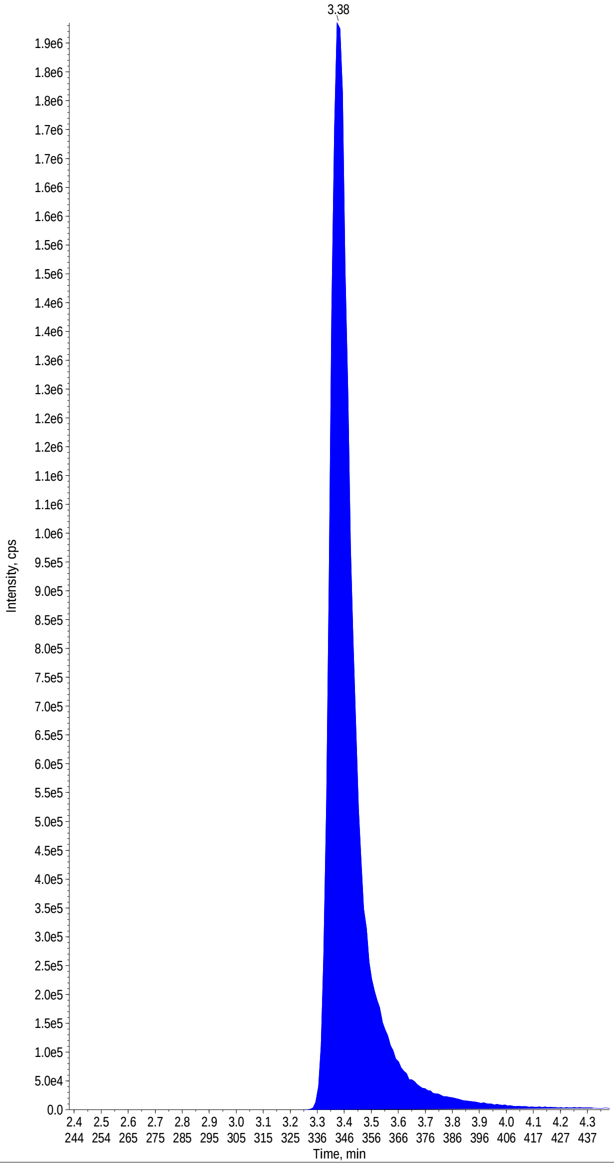

Sample Name: "LLOQ2" Sample ID: "" File: "20220801-1.wiff"
Peak Name: "OHMPT" Mass(es): "235.200/150.200 Da"
Comment: "" Annotation: ""

```
Sample Index:       37
Sample Type:        Unknown
Concentration:      N/A
Calculated Conc:    0.00    ng/mL
Acq. Date:          8/1/2022
Acq. Time:          3:43:40 PM

Modified:           No
Proc. Algorithm: Specify Parameters - MQ III
Noise Percentage:     50
Base. Sub. Window:    1.00   min
Peak-Split. Factor:   2
Report Largest Peak:   Yes
Min. Peak Height:     300.00   cps
Min. Peak Width:       0.00    sec
Smoothing Width:       9       points
RT Window:          30.0    sec
Expected RT:        1.81    min
Use Relative RT:    No

Int. Type:          Valley
Retention Time:     1.80    min
Area:            2.08e+004   counts
Height:            2.58e+003  cps
Start Time:         1.67    min
End Time:           2.23    min
```

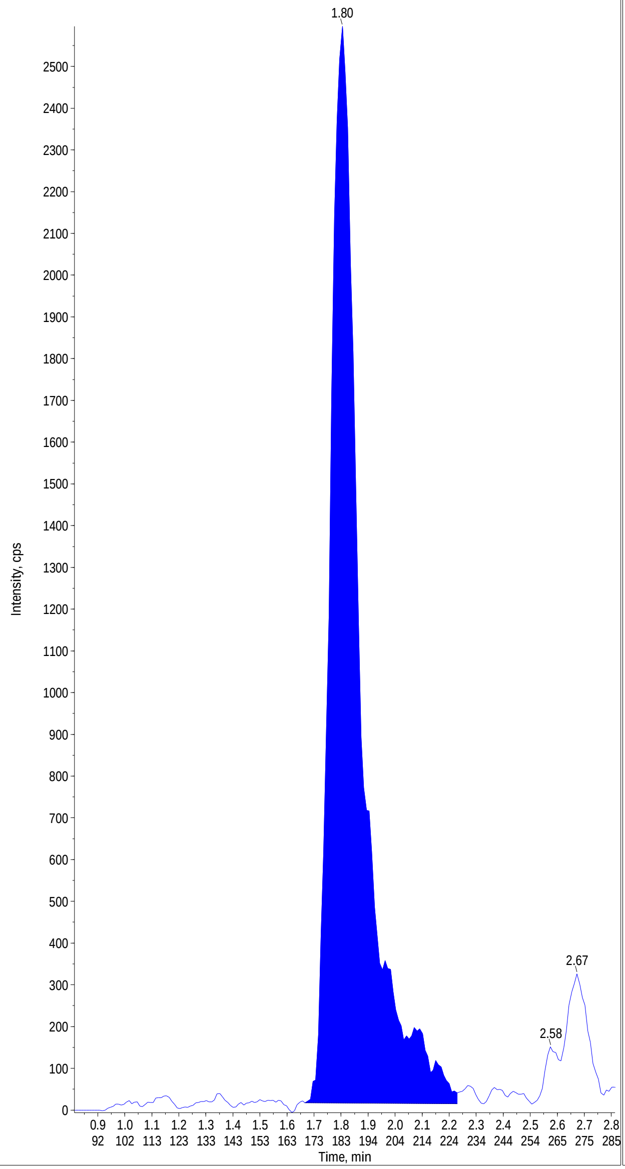

Sample Name: "LLOQ2" Sample ID: "" File: "20220801-1.wiff"
Peak Name: "DXP(IS)" Mass(es): "285.500/154.100 Da"
Comment: "" Annotation: ""

```
Sample Index:       37
Sample Type:        Unknown
Concentration:      1.00    ng/mL
Calculated Conc:    N/A
Acq. Date:          8/1/2022
Acq. Time:          3:43:40 PM

Modified:           No
Proc. Algorithm: Specify Parameters - MQ III
Noise Percentage:     50
Base. Sub. Window:    1.00   min
Peak-Split. Factor:   2
Report Largest Peak:   Yes
Min. Peak Height:     500.00   cps
Min. Peak Width:       0.00    sec
Smoothing Width:       0       points
RT Window:          30.0    sec
Expected RT:        3.38    min
Use Relative RT:    No

Int. Type:          Base To Base
Retention Time:     3.38    min
Area:            1.03e+007   counts
Height:            1.74e+006  cps
Start Time:         3.26    min
End Time:           4.34    min
```

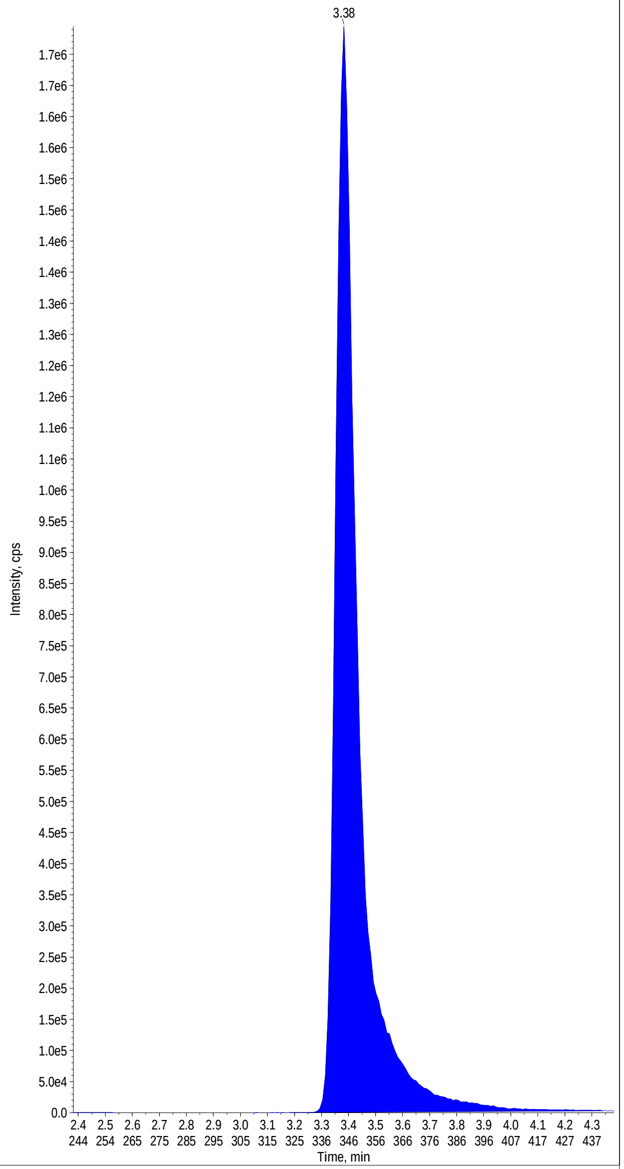

Sample Name: "LLOQ3" Sample ID: "" File: "20220801-1.wiff"
Peak Name: "OHMPT" Mass(es): "235.200/150.200 Da"
Comment: "" Annotation: ""

```
Sample Index:       38
Sample Type:        Unknown
Concentration:      N/A
Calculated Conc:    0.00     ng/mL
Acq. Date:          8/1/2022
Acq. Time:          3:48:44 PM

Modified:           No
Proc. Algorithm: Specify Parameters - MQ III
Noise Percentage:   50
Base. Sub. Window:  1.00    min
Peak-Split. Factor: 2
Report Largest Peak:  Yes
Min. Peak Height:   300.00   cps
Min. Peak Width:    0.00     sec
Smoothing Width:    9        points
RT Window:          30.0     sec
Expected RT:        1.81     min
Use Relative RT:    No

Int. Type:          Valley
Retention Time:     1.81     min
Area:               2.03e+004  counts
Height:             2.53e+003  cps
Start Time:         1.69     min
End Time:           2.15     min
```

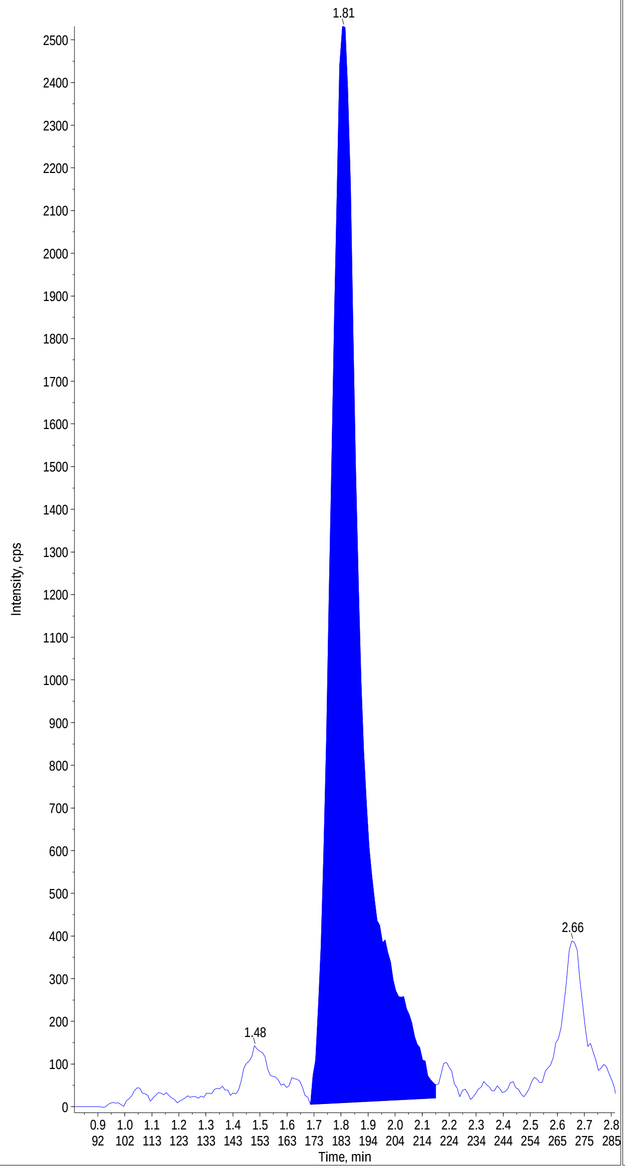

Sample Name: "LLOQ3" Sample ID: "" File: "20220801-1.wiff"
Peak Name: "DXP(IS)" Mass(es): "285.500/154.100 Da"
Comment: "" Annotation: ""

```
Sample Index:       38
Sample Type:        Unknown
Concentration:      1.00     ng/mL
Calculated Conc:    N/A
Acq. Date:          8/1/2022
Acq. Time:          3:48:44 PM

Modified:           No
Proc. Algorithm: Specify Parameters - MQ III
Noise Percentage:   50
Base. Sub. Window:  1.00    min
Peak-Split. Factor: 2
Report Largest Peak:  Yes
Min. Peak Height:   500.00   cps
Min. Peak Width:    0.00     sec
Smoothing Width:    0        points
RT Window:          30.0     sec
Expected RT:        3.38     min
Use Relative RT:    No

Int. Type:          Base To Base
Retention Time:     3.38     min
Area:               1.05e+007  counts
Height:             1.73e+006  cps
Start Time:         3.26     min
End Time:           4.31     min
```

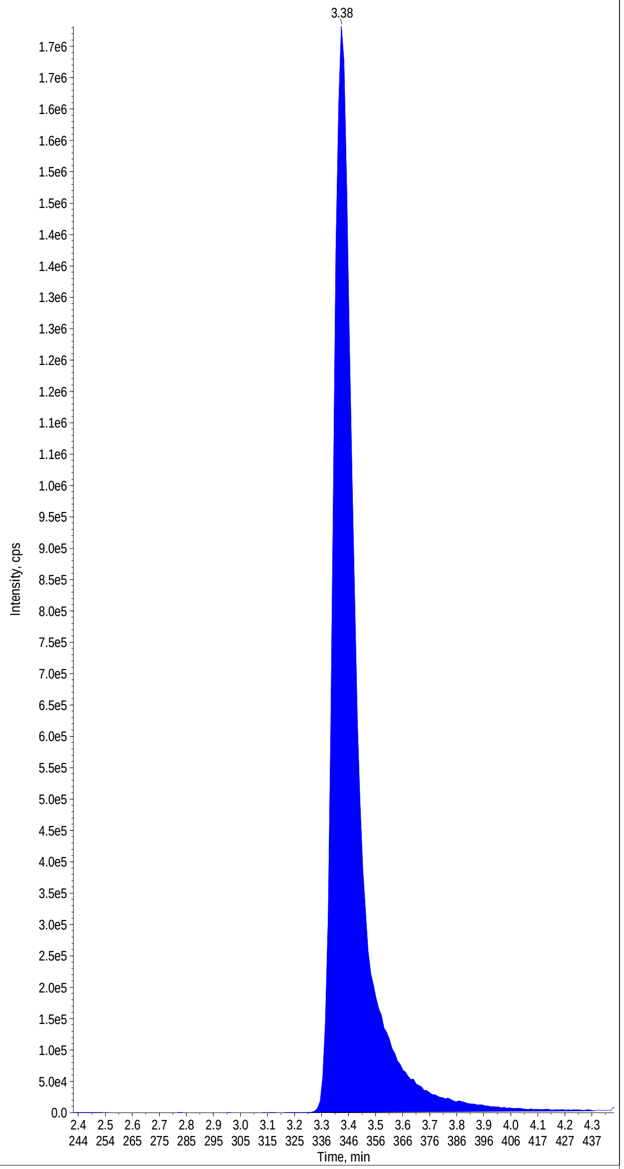

Sample Name: "LLOQ4"   Sample ID: ""   File: "20220801-1.wiff"
Peak Name: "OHMPT"   Mass(es): "235.200/150.200 Da"
Comment: ""   Annotation: ""

```
Sample Index:       39
Sample Type:      Unknown
Concentration:      N/A
Calculated Conc:    0.00    ng/mL
Acq. Date:        8/1/2022
Acq. Time:        3:53:47 PM

Modified:           No
Proc. Algorithm: Specify Parameters - MQ III
Noise Percentage:     50
Base. Sub. Window:    1.00   min
Peak-Split. Factor:   2
Report Largest Peak:   Yes
Min. Peak Height:    300.00   cps
Min. Peak Width:       0.00   sec
Smoothing Width:       9       points
RT Window:          30.0    sec
Expected RT:        1.81    min
Use Relative RT:    No

Int. Type:        Base To Base
Retention Time:     1.80    min
Area:          2.15e+004    counts
Height:          2.74e+003   cps
Start Time:         1.68    min
End Time:           2.27    min
```

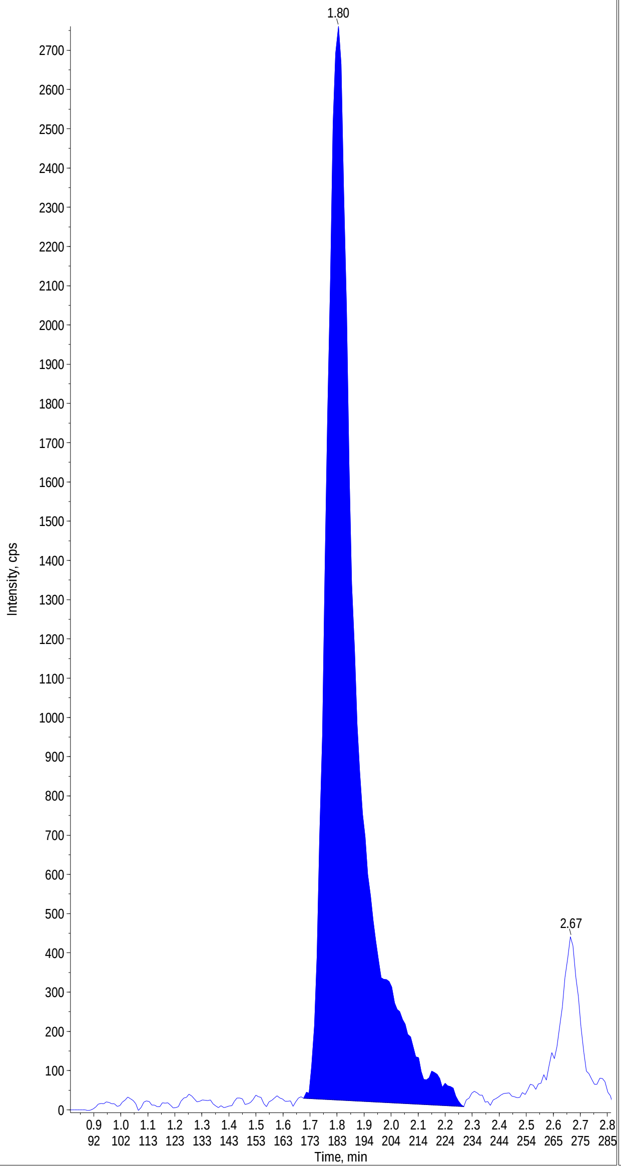

Sample Name: "LLOQ4"   Sample ID: ""   File: "20220801-1.wiff"
Peak Name: "DXP(IS)"   Mass(es): "285.500/154.100 Da"
Comment: ""   Annotation: ""

```
Sample Index:       39
Sample Type:      Unknown
Concentration:      1.00    ng/mL
Calculated Conc:    N/A
Acq. Date:        8/1/2022
Acq. Time:        3:53:47 PM

Modified:           No
Proc. Algorithm: Specify Parameters - MQ III
Noise Percentage:     50
Base. Sub. Window:    1.00   min
Peak-Split. Factor:   2
Report Largest Peak:   Yes
Min. Peak Height:    500.00   cps
Min. Peak Width:       0.00   sec
Smoothing Width:       0       points
RT Window:          30.0    sec
Expected RT:        3.38    min
Use Relative RT:    No

Int. Type:        Base To Base
Retention Time:     3.38    min
Area:          1.04e+007    counts
Height:          1.69e+006   cps
Start Time:         3.26    min
End Time:           4.31    min
```

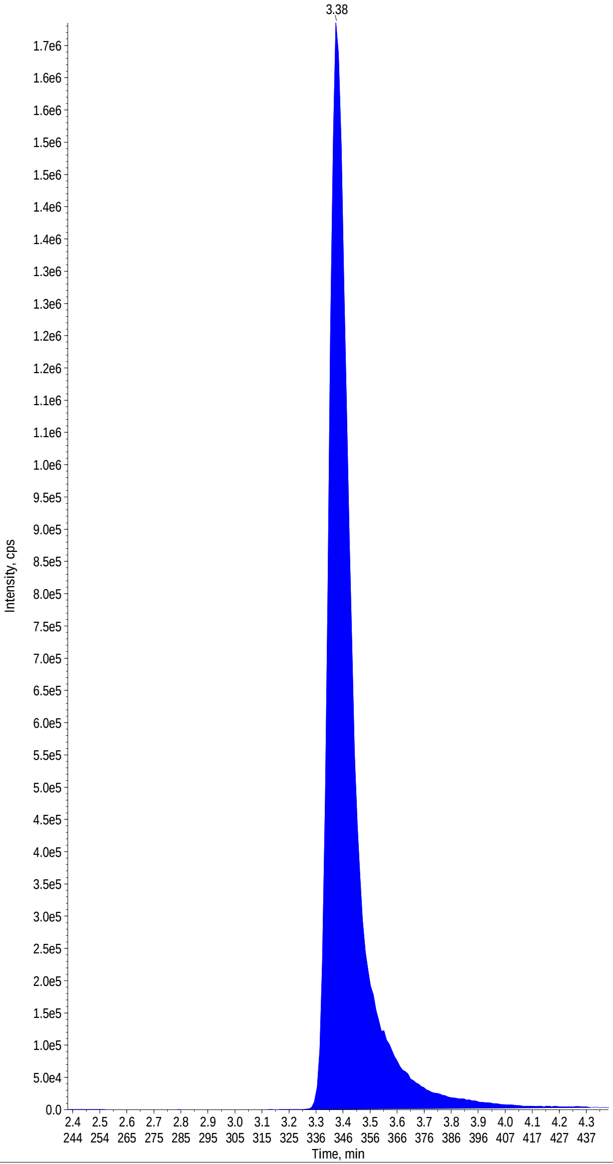

Sample Name: "LLOQ5"   Sample ID: ""   File: "20220801-1.wiff"
Peak Name: "OHMPT"   Mass(es): "235.200/150.200 Da"
Comment: ""   Annotation: ""

```
Sample Index:       40
Sample Type:      Unknown
Concentration:       N/A
Calculated Conc:     0.00     ng/mL
Acq. Date:        8/1/2022
Acq. Time:        3:58:48 PM

Modified:            No
Proc. Algorithm: Specify Parameters - MQ III
Noise Percentage:      50
Base. Sub. Window:     1.00    min
Peak-Split. Factor:    2
Report Largest Peak:   Yes
Min. Peak Height:     300.00    cps
Min. Peak Width:        0.00    sec
Smoothing Width:        9        points
RT Window:           30.0     sec
Expected RT:         1.81     min
Use Relative RT:     No

Int. Type:         Valley
Retention Time:      1.81     min
Area:          2.16e+004    counts
Height:          2.55e+003   cps
Start Time:          1.63     min
End Time:            2.15     min
```

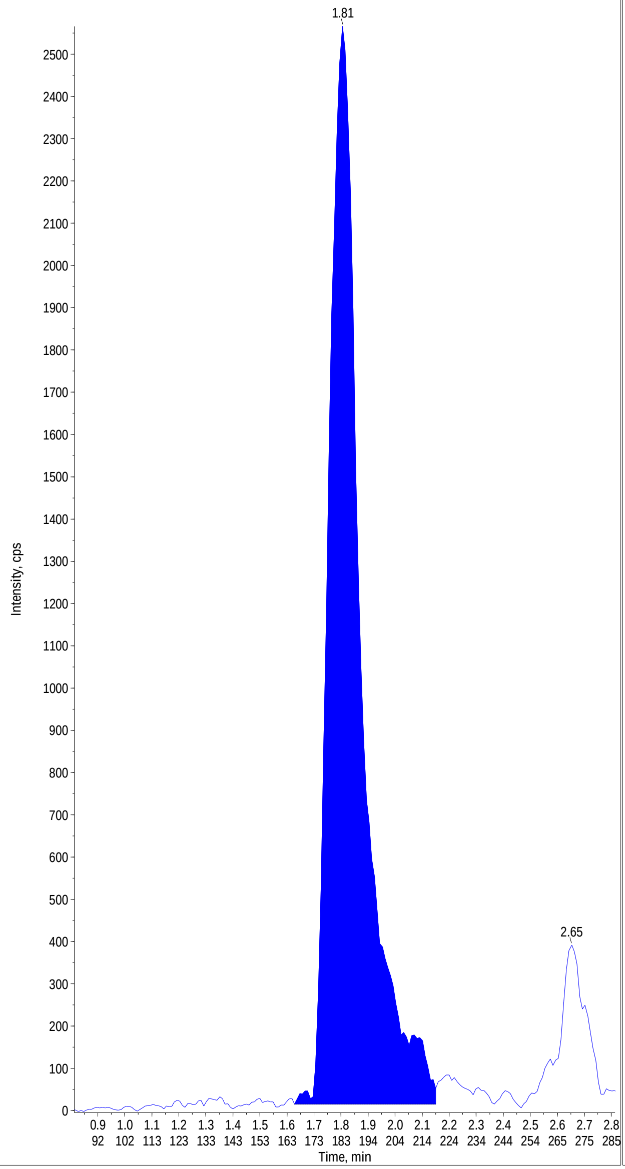

Sample Name: "LLOQ5"   Sample ID: ""   File: "20220801-1.wiff"
Peak Name: "DXP(IS)"   Mass(es): "285.500/154.100 Da"
Comment: ""   Annotation: ""

```
Sample Index:       40
Sample Type:      Unknown
Concentration:       1.00     ng/mL
Calculated Conc:     N/A
Acq. Date:        8/1/2022
Acq. Time:        3:58:48 PM

Modified:            No
Proc. Algorithm: Specify Parameters - MQ III
Noise Percentage:      50
Base. Sub. Window:     1.00    min
Peak-Split. Factor:    2
Report Largest Peak:   Yes
Min. Peak Height:     500.00    cps
Min. Peak Width:        0.00    sec
Smoothing Width:        0        points
RT Window:           30.0     sec
Expected RT:         3.38     min
Use Relative RT:     No

Int. Type:         Base To Base
Retention Time:      3.37     min
Area:          1.05e+007    counts
Height:          1.77e+006   cps
Start Time:          3.24     min
End Time:            4.32     min
```

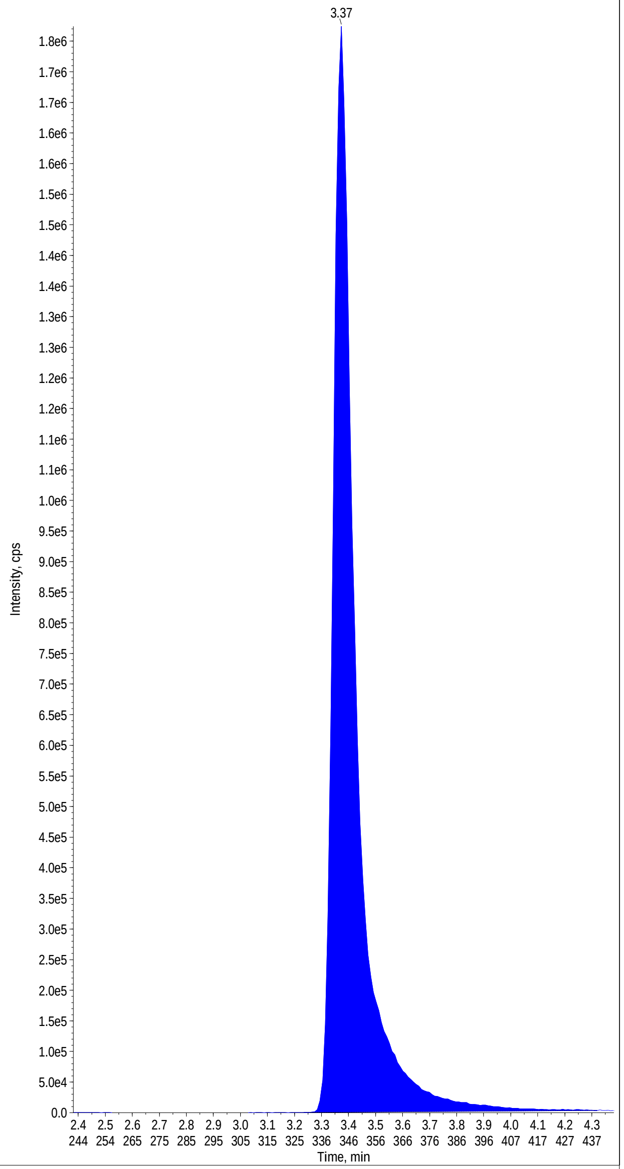

Sample Name: "LLOQ6"   Sample ID: ""   File: "20220801-1.wiff"
Peak Name: "OHMPT"   Mass(es): "235.200/150.200 Da"
Comment: ""   Annotation: ""

Sample Index:	41	
Sample Type:	Unknown	
Concentration:	N/A	
Calculated Conc:	0.00	ng/mL
Acq. Date:	8/1/2022	
Acq. Time:	4:03:50 PM	
Modified:	No	
Proc. Algorithm:	Specify Parameters - MQ III	
Noise Percentage:	50	
Base. Sub. Window:	1.00	min
Peak-Split. Factor:	2	
Report Largest Peak:	Yes	
Min. Peak Height:	300.00	cps
Min. Peak Width:	0.00	sec
Smoothing Width:	9	points
RT Window:	30.0	sec
Expected RT:	1.81	min
Use Relative RT:	No	
Int. Type:	Valley	
Retention Time:	1.80	min
Area:	1.93e+004	counts
Height:	2.45e+003	cps
Start Time:	1.67	min
End Time:	2.04	min

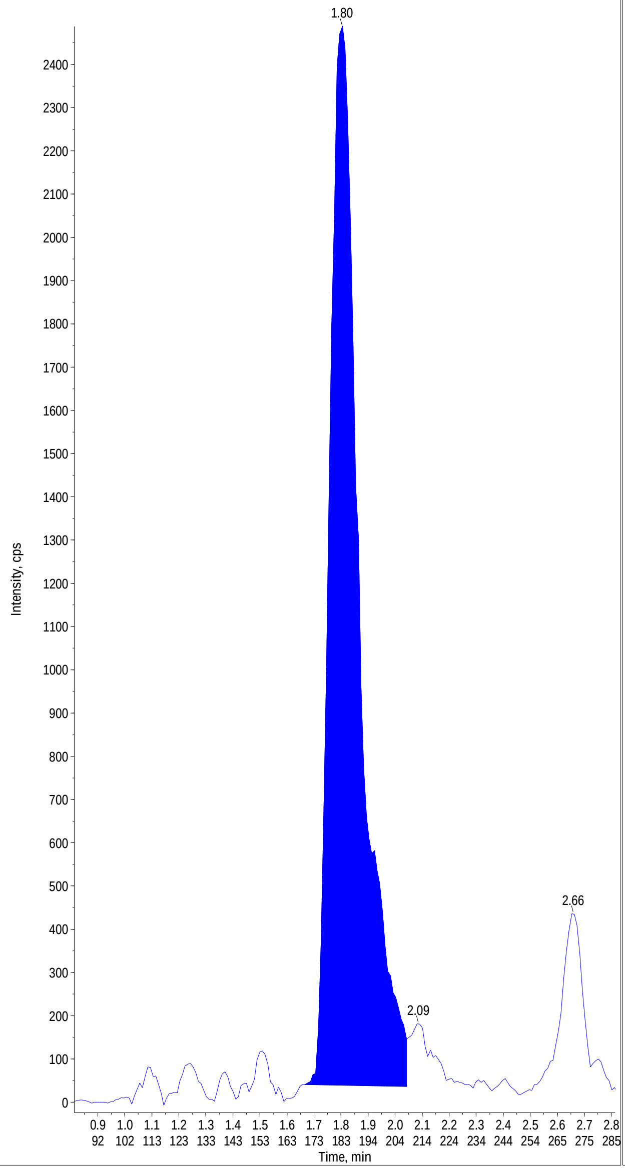

Sample Name: "LLOQ6"   Sample ID: ""   File: "20220801-1.wiff"
Peak Name: "DXP(IS)"   Mass(es): "285.500/154.100 Da"
Comment: ""   Annotation: ""

Sample Index:	41	
Sample Type:	Unknown	
Concentration:	1.00	ng/mL
Calculated Conc:	N/A	
Acq. Date:	8/1/2022	
Acq. Time:	4:03:50 PM	
Modified:	No	
Proc. Algorithm:	Specify Parameters - MQ III	
Noise Percentage:	50	
Base. Sub. Window:	1.00	min
Peak-Split. Factor:	2	
Report Largest Peak:	Yes	
Min. Peak Height:	500.00	cps
Min. Peak Width:	0.00	sec
Smoothing Width:	0	points
RT Window:	30.0	sec
Expected RT:	3.38	min
Use Relative RT:	No	
Int. Type:	Base To Base	
Retention Time:	3.38	min
Area:	1.04e+007	counts
Height:	1.77e+006	cps
Start Time:	3.25	min
End Time:	4.32	min

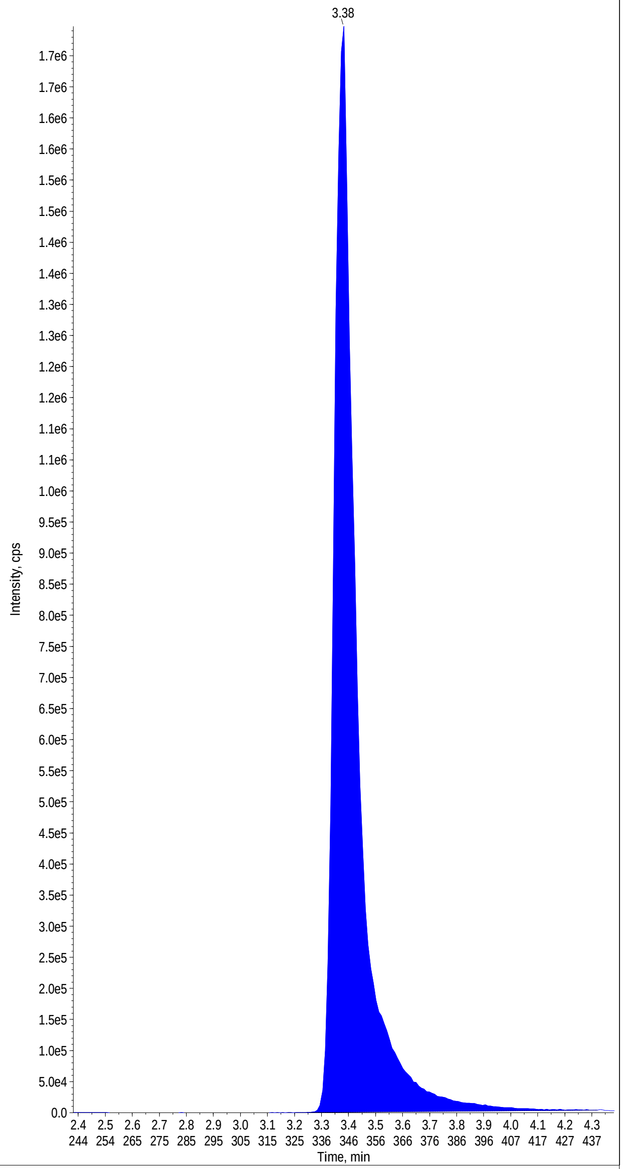

Sample Name: "MEOH" Sample ID: "" File: "20220801-1.wiff"
Peak Name: "OHMPT" Mass(es): "235.200/150.200 Da"
Comment: "" Annotation: ""

```
Sample Index:       42
Sample Type:      Unknown
Concentration:       N/A
Calculated Conc:     0.00     ng/mL
Acq. Date:        8/1/2022
Acq. Time:        6:50:32 PM

Modified:            No
```

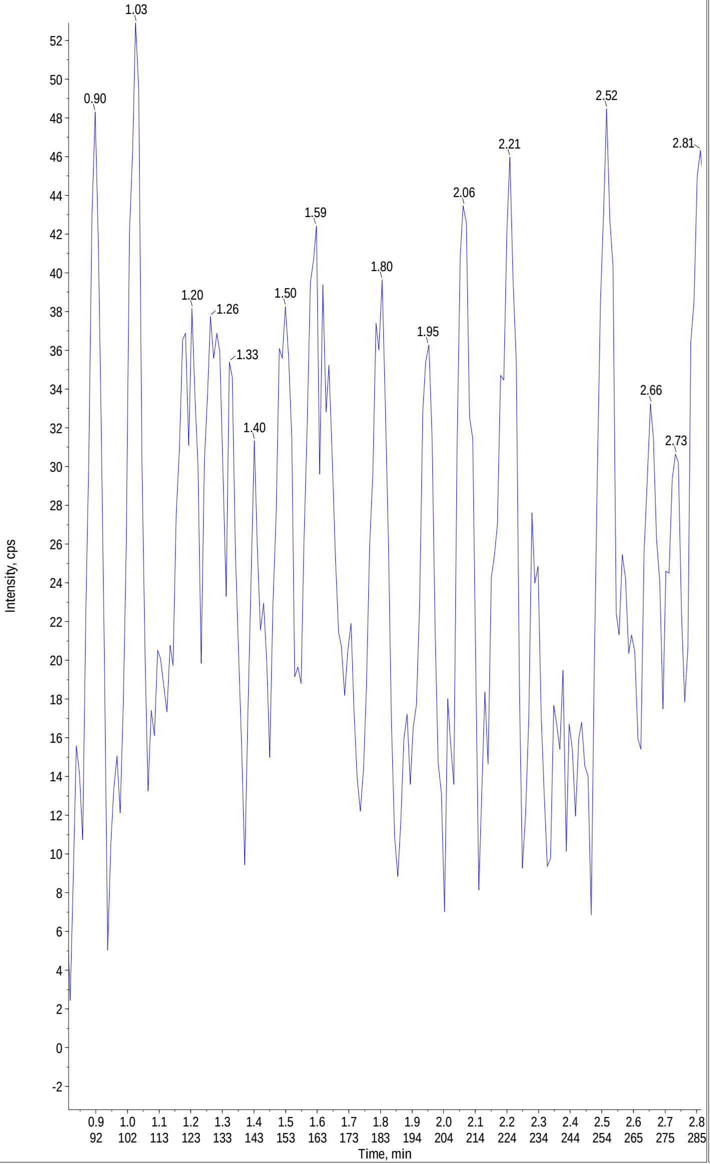

Sample Name: "MEOH" Sample ID: "" File: "20220801-1.wiff"
Peak Name: "DXP(IS)" Mass(es): "285.500/154.100 Da"
Comment: "" Annotation: ""

```
Sample Index:       42
Sample Type:      Unknown
Concentration:       1.00     ng/mL
Calculated Conc:     N/A
Acq. Date:        8/1/2022
Acq. Time:        6:50:32 PM

Modified:            No
Proc. Algorithm: Specify Parameters - MQ III
Noise Percentage:     50
Base. Sub. Window:     1.00    min
Peak-Split. Factor:   2
Report Largest Peak:    Yes
Min. Peak Height:     500.00    cps
Min. Peak Width:        0.00    sec
Smoothing Width:        0        points
RT Window:          30.0     sec
Expected RT:         3.38    min
Use Relative RT:     No

Int. Type:        Base To Base
Retention Time:      3.32    min
Area:          2.15e+004    counts
Height:          1.45e+003   cps
Start Time:          3.07    min
End Time:            3.73    min
```

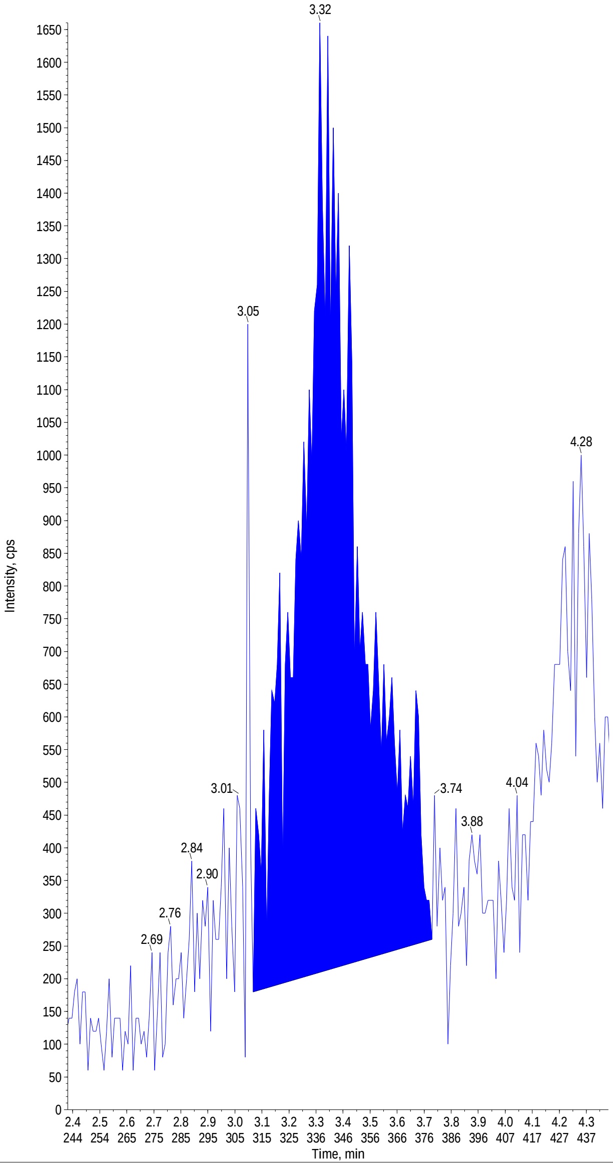

Supplement: Supplemental Information 1 — The raw data for sucrose preference, average moving speed, total moving distance, freezing time, times entering the central area, cortex 5-HT level, plasma clopidogrel level, pharmacokinetic parameters of clopidogrel in rats’ plasma , CYP450 activity and expression in rat livers (Fig. 2–Fig. 5, and Tables 2 and 3) [file peerj-10-14111-s001.zip › OHMPT.pdf]
