# Supplementary material for: Effect of chronic unpredicted mild stress-induced depression on clopidogrel pharmacokinetics in rats
Source: PeerJ. 2022 Oct 3;10:e14111. doi: 10.7717/peerj.14111 (PMC9536304; doi:10.7717/peerj.14111)

Sample Name: "MEOH" Sample ID: "" File: "20220801-1.wiff"
Peak Name: "OHTOL" Mass(es): "287.300/170.800 Da"
Comment: "" Annotation: ""

Sample Index:	3
Sample Type:	Unknown
Concentration:	N/A
Calculated Conc:	No Intercept
Acq. Date:	8/1/2022
Acq. Time:	11:27:52 AM
Modified:	No

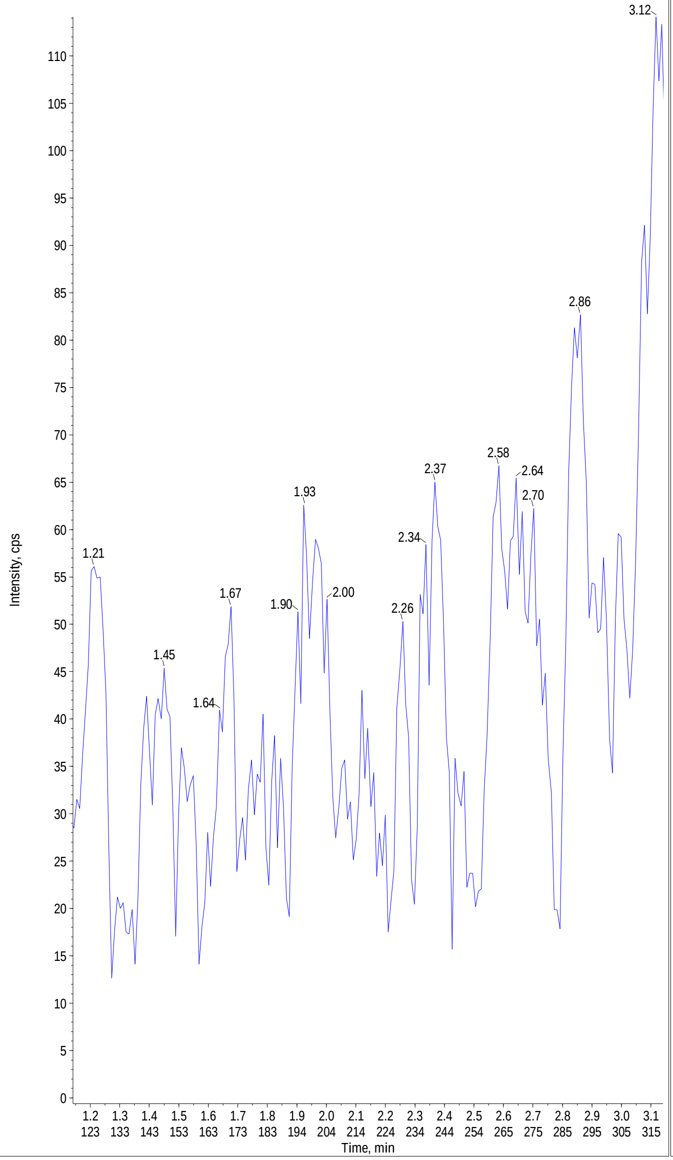

Sample Name: "MEOH" Sample ID: "" File: "20220801-1.wiff"
Peak Name: "DXP(IS)" Mass(es): "285.500/154.100 Da"
Comment: "" Annotation: ""

Sample Index:	3
Sample Type:	Unknown
Concentration:	1.00 ng/mL
Calculated Conc:	N/A
Acq. Date:	8/1/2022
Acq. Time:	11:27:52 AM
Modified:	No

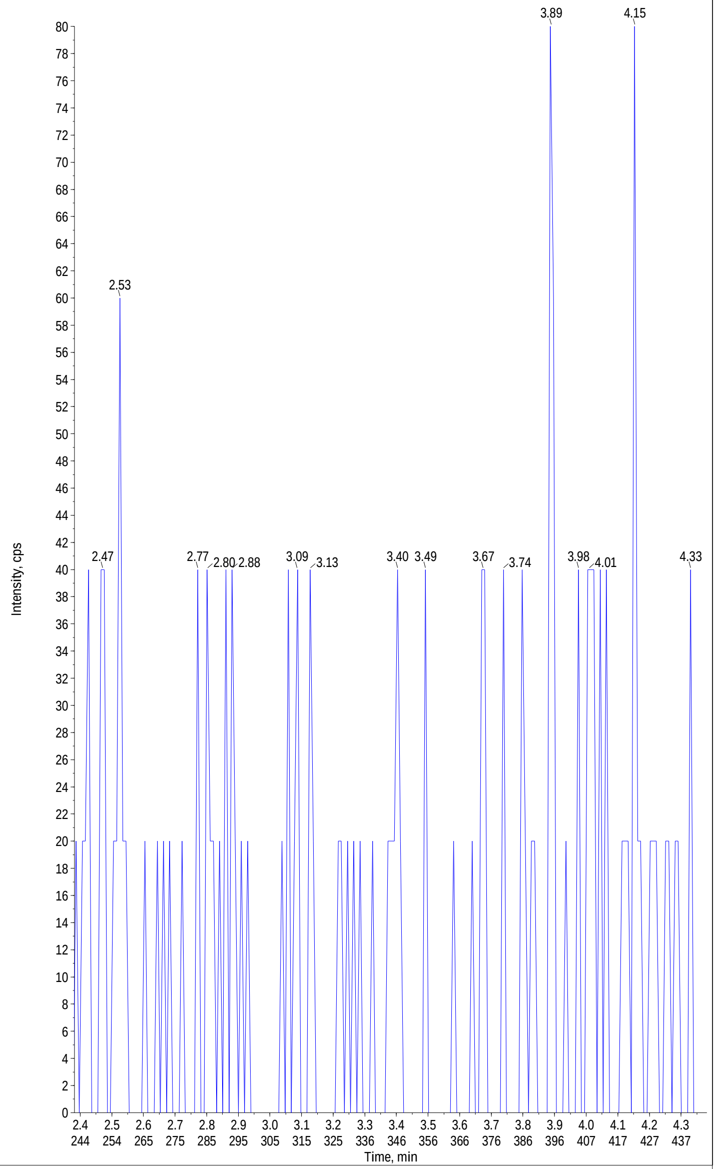

Sample Name: "KB1" Sample ID: "" File: "20220801-1.wiff"
Peak Name: "OHTOL" Mass(es): "287.300/170.800 Da"
Comment: "" Annotation: ""

Sample Index: 4
Sample Type: Unknown
Concentration: N/A
Calculated Conc: No Intercept
Acq. Date: 8/1/2022
Acq. Time: 11:32:55 AM

Modified: No

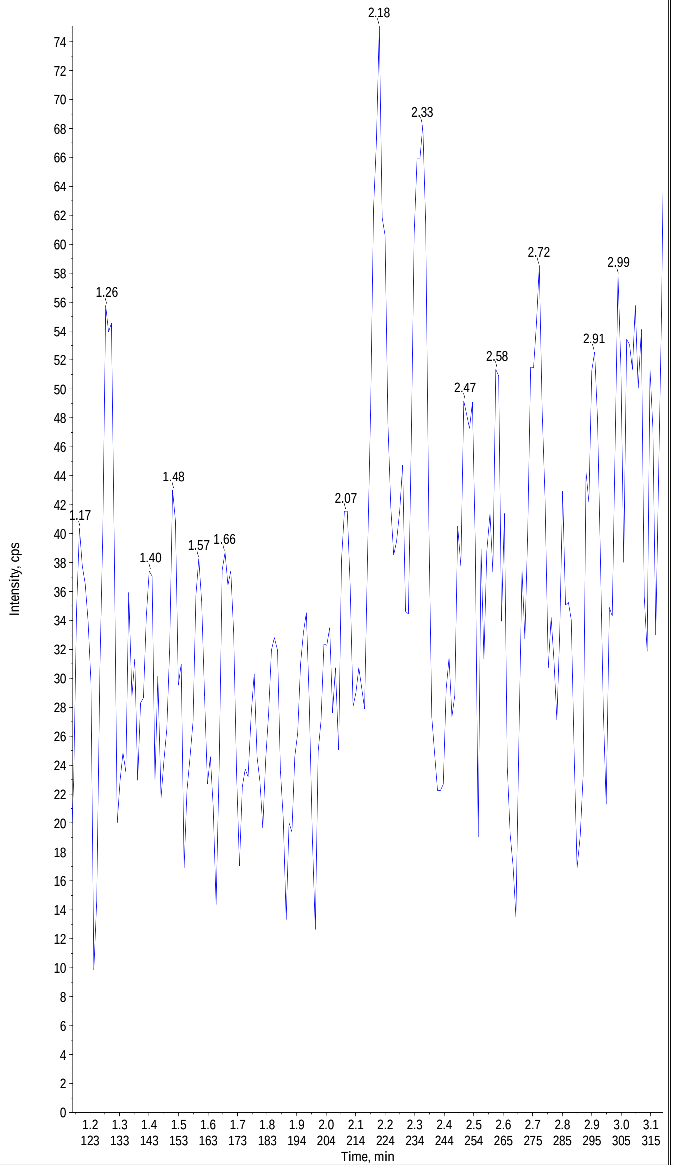

Sample Name: "KB1" Sample ID: "" File: "20220801-1.wiff"
Peak Name: "DXP(IS)" Mass(es): "285.500/154.100 Da"
Comment: "" Annotation: ""

Sample Index: 4
Sample Type: Unknown
Concentration: 1.00 ng/mL
Calculated Conc: N/A
Acq. Date: 8/1/2022
Acq. Time: 11:32:55 AM

Modified: No

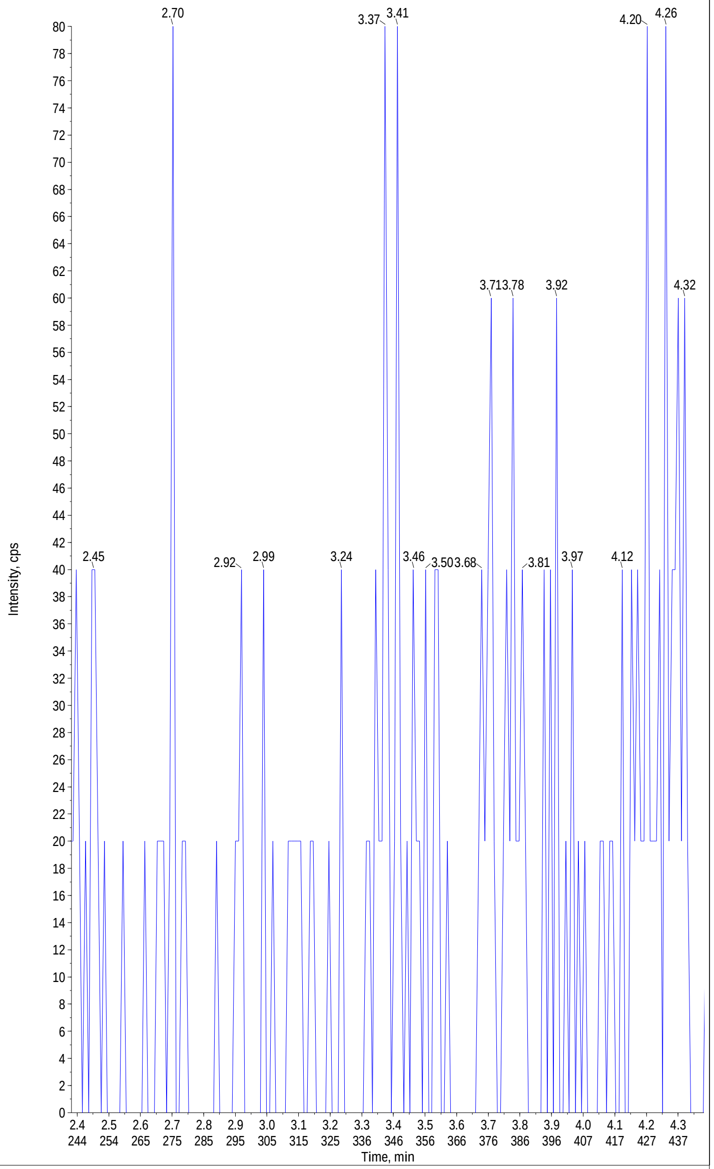

Sample Name: "KB2" Sample ID: "" File: "20220801-1.wiff"
Peak Name: "OHTOL" Mass(es): "287.300/170.800 Da"
Comment: "" Annotation: ""

Sample Index:	5
Sample Type:	Unknown
Concentration:	N/A
Calculated Conc:	No Intercept
Acq. Date:	8/1/2022
Acq. Time:	11:37:57 AM
Modified:	No

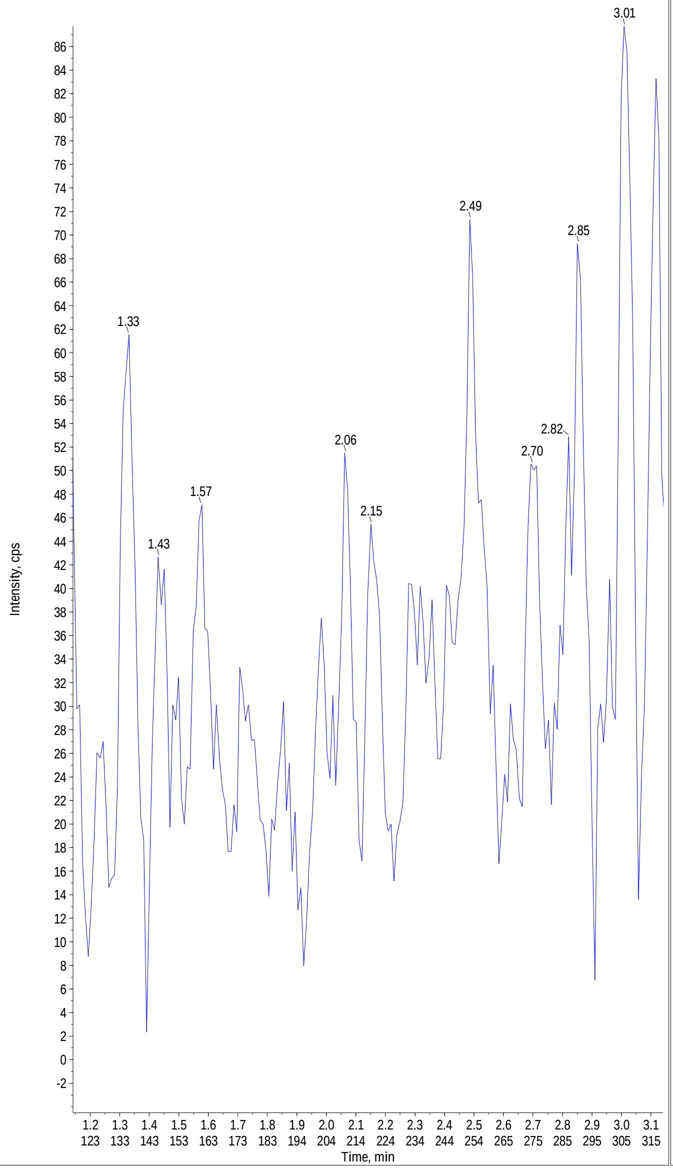

Sample Name: "KB2" Sample ID: "" File: "20220801-1.wiff"
Peak Name: "DXP(IS)" Mass(es): "285.500/154.100 Da"
Comment: "" Annotation: ""

Sample Index:	5
Sample Type:	Unknown
Concentration:	1.00 ng/mL
Calculated Conc:	N/A
Acq. Date:	8/1/2022
Acq. Time:	11:37:57 AM
Modified:	No

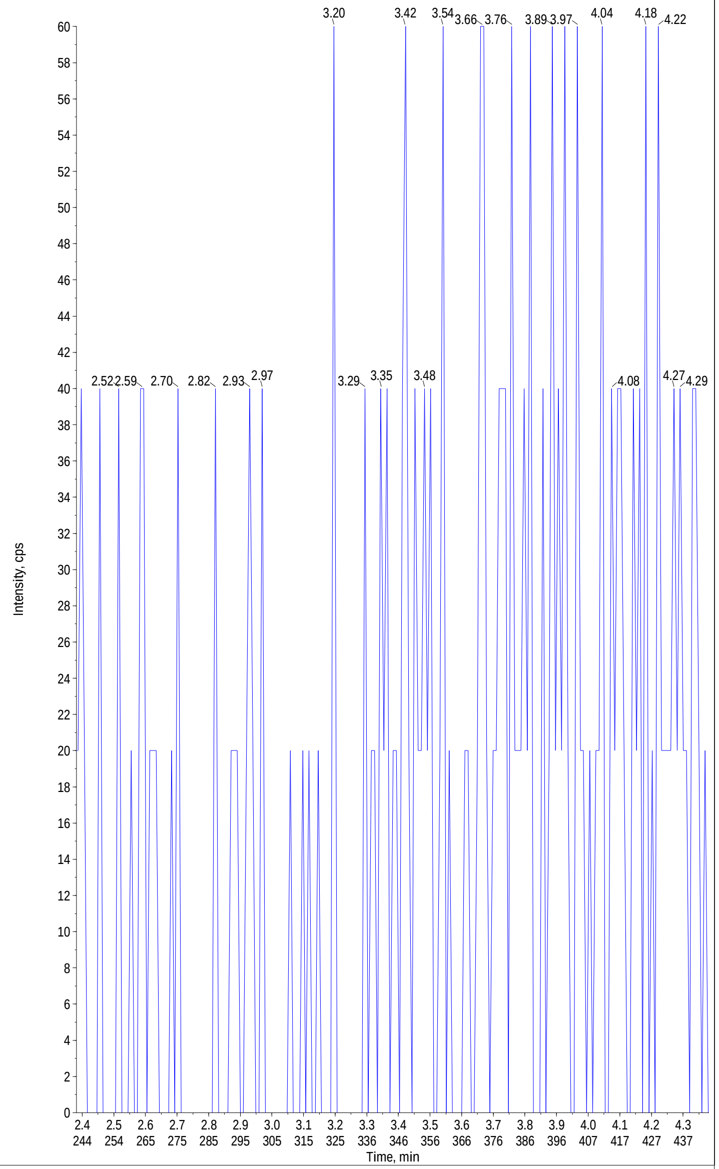

Sample Name: "KB3"   Sample ID: ""   File: "20220801-1.wiff"
Peak Name: "OHTOL"   Mass(es): "287.300/170.800 Da"
Comment: ""   Annotation: ""

```
Sample Index:        6
Sample Type:         Unknown
Concentration:       N/A
Calculated Conc:     No Intercept
Acq. Date:           8/1/2022
Acq. Time:           11:42:59 AM

Modified:            No
```

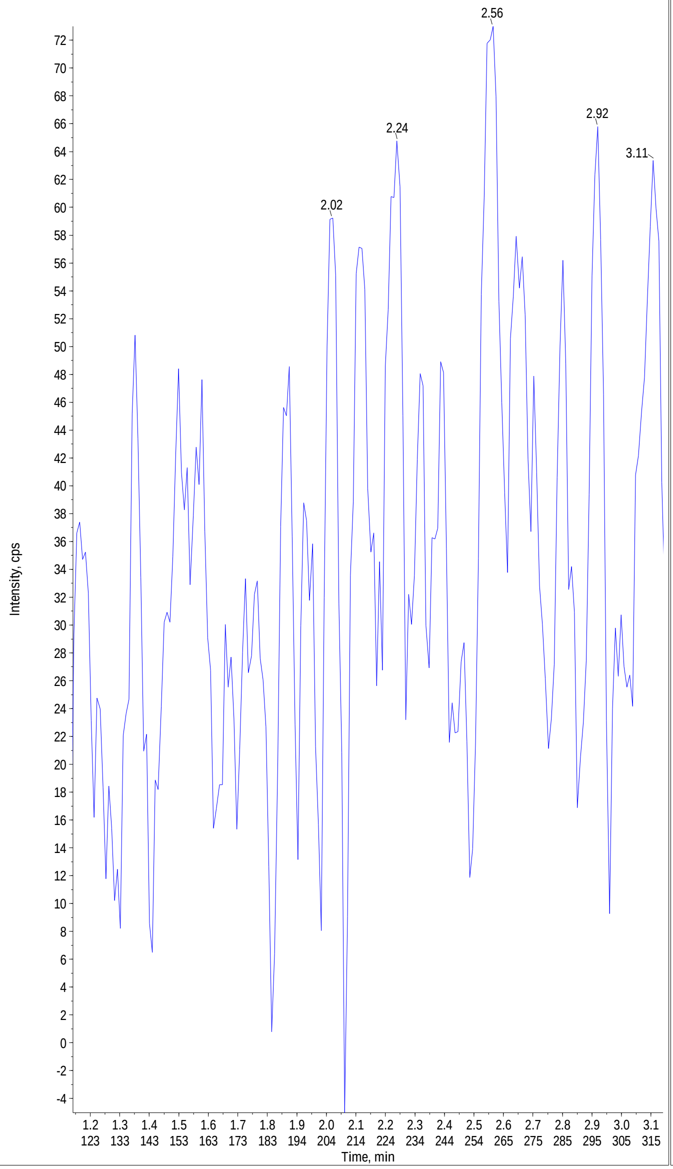

Sample Name: "KB3"   Sample ID: ""   File: "20220801-1.wiff"
Peak Name: "DXP(IS)"   Mass(es): "285.500/154.100 Da"
Comment: ""   Annotation: ""

```
Sample Index:        6
Sample Type:         Unknown
Concentration:       1.00    ng/mL
Calculated Conc:     N/A
Acq. Date:           8/1/2022
Acq. Time:           11:42:59 AM

Modified:            No
```

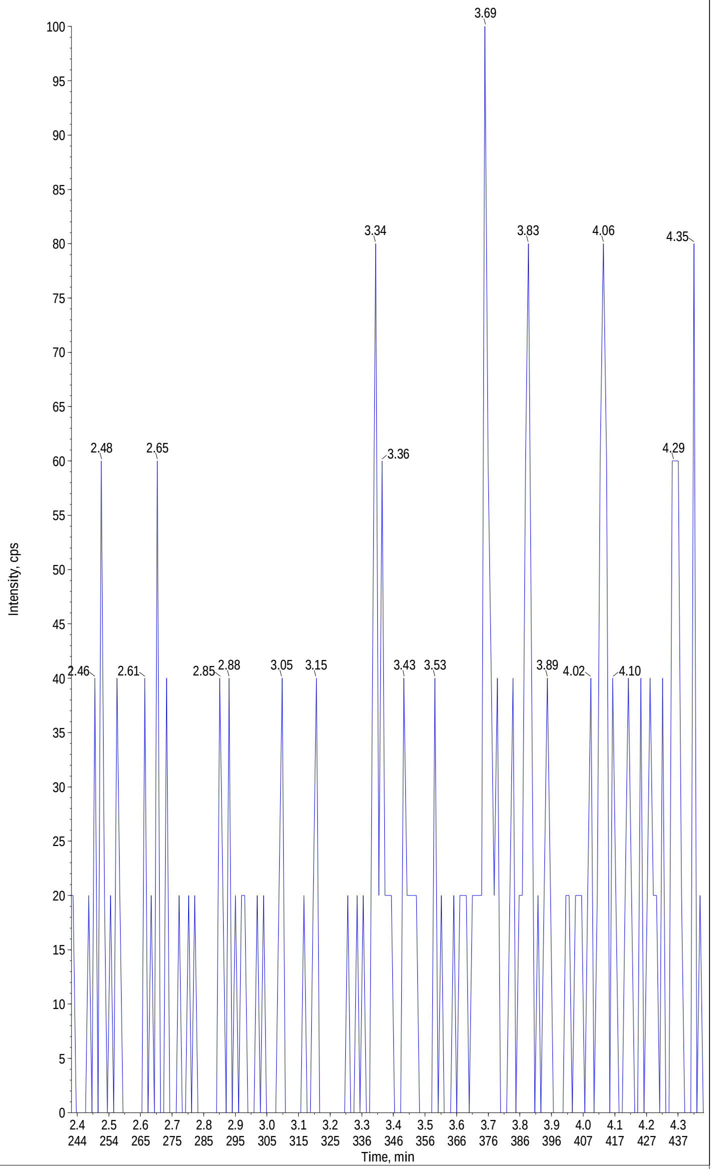

Sample Name: "KB4" Sample ID: "" File: "20220801-1.wiff"
Peak Name: "OHTOL" Mass(es): "287.300/170.800 Da"
Comment: "" Annotation: ""

```
Sample Index:       7
Sample Type:        Unknown
Concentration:      N/A
Calculated Conc:    No Intercept
Acq. Date:          8/1/2022
Acq. Time:          11:48:02 AM

Modified:           No
```

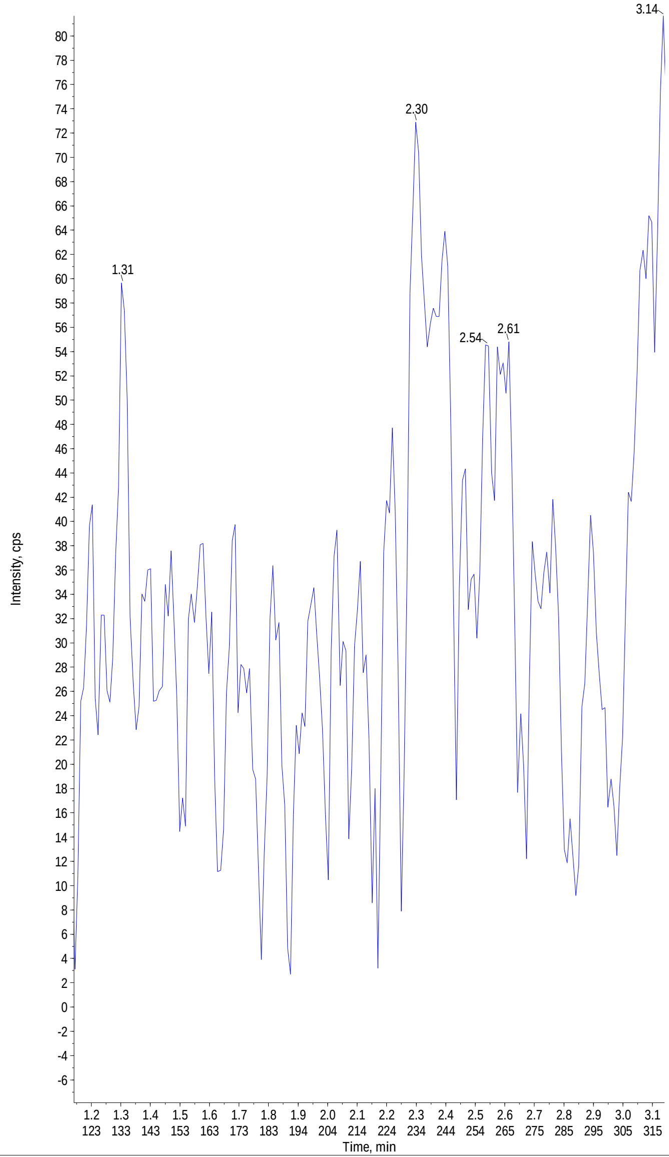

Sample Name: "KB4" Sample ID: "" File: "20220801-1.wiff"
Peak Name: "DXP(IS)" Mass(es): "285.500/154.100 Da"
Comment: "" Annotation: ""

```
Sample Index:       7
Sample Type:        Unknown
Concentration:      1.00    ng/mL
Calculated Conc:    N/A
Acq. Date:          8/1/2022
Acq. Time:          11:48:02 AM

Modified:           No
```

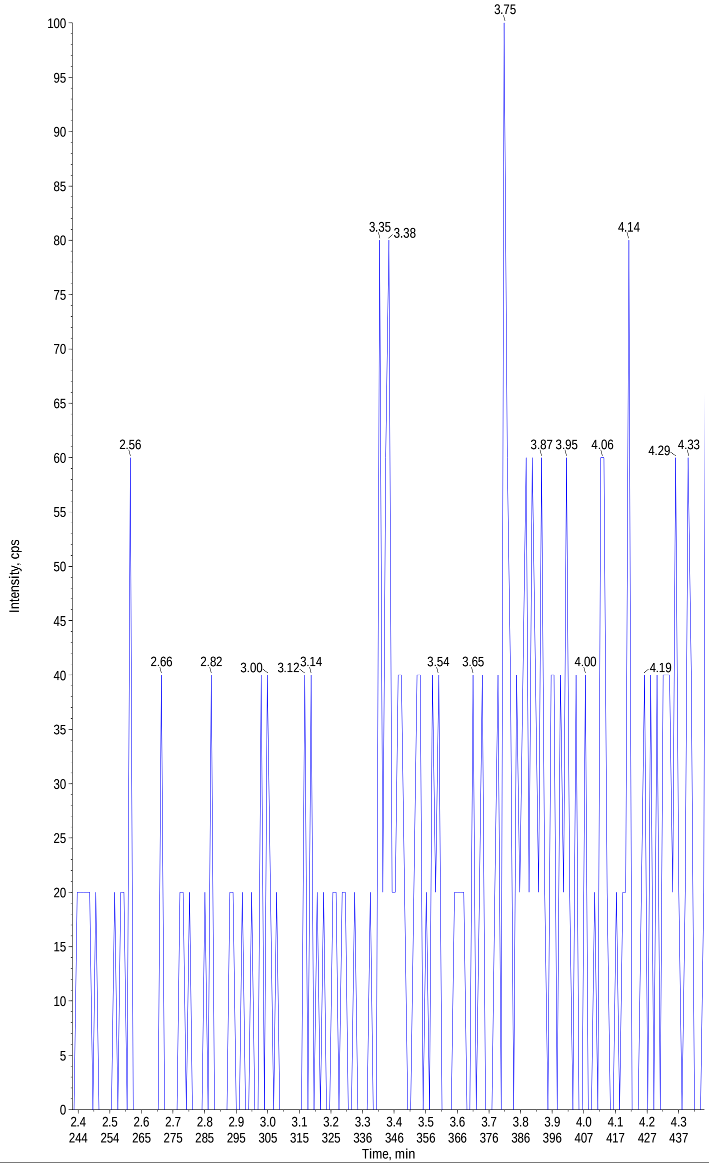

Sample Name: "KB5"   Sample ID: ""   File: "20220801-1.wiff"
Peak Name: "OHTOL"   Mass(es): "287.300/170.800 Da"
Comment: ""   Annotation: ""

Sample Index: 8
Sample Type: Unknown
Concentration: N/A
Calculated Conc: No Intercept
Acq. Date: 8/1/2022
Acq. Time: 11:53:05 AM

Modified: No

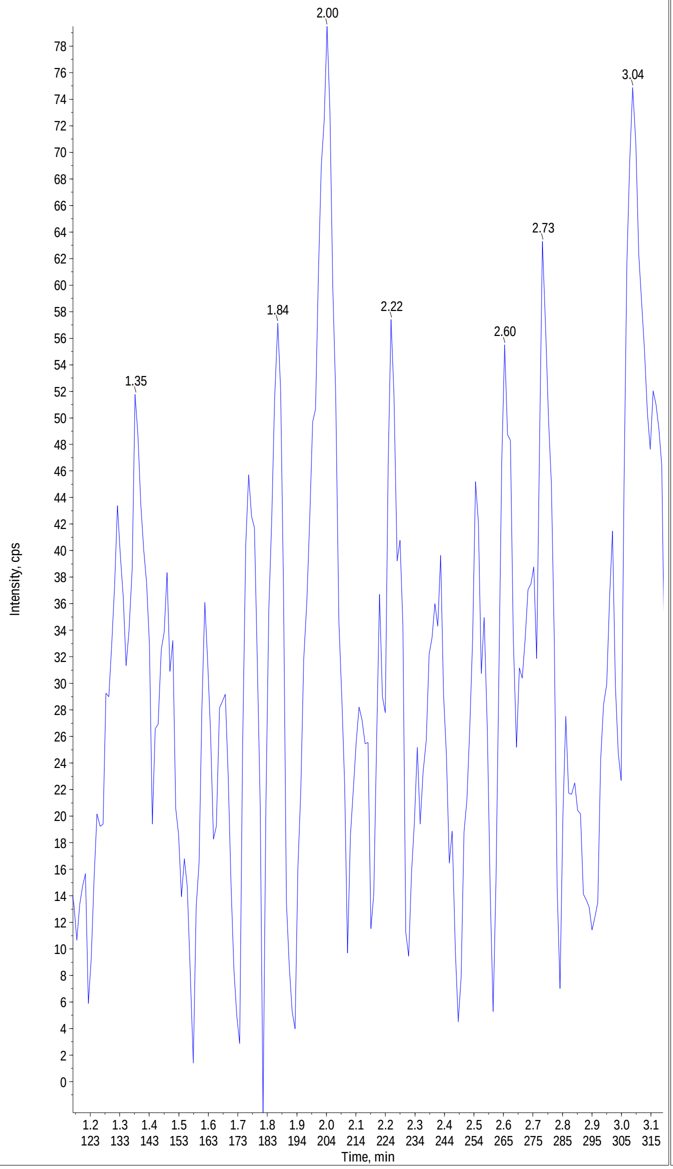

Sample Name: "KB5"   Sample ID: ""   File: "20220801-1.wiff"
Peak Name: "DXP(IS)"   Mass(es): "285.500/154.100 Da"
Comment: ""   Annotation: ""

Sample Index: 8
Sample Type: Unknown
Concentration: 1.00 ng/mL
Calculated Conc: N/A
Acq. Date: 8/1/2022
Acq. Time: 11:53:05 AM

Modified: No

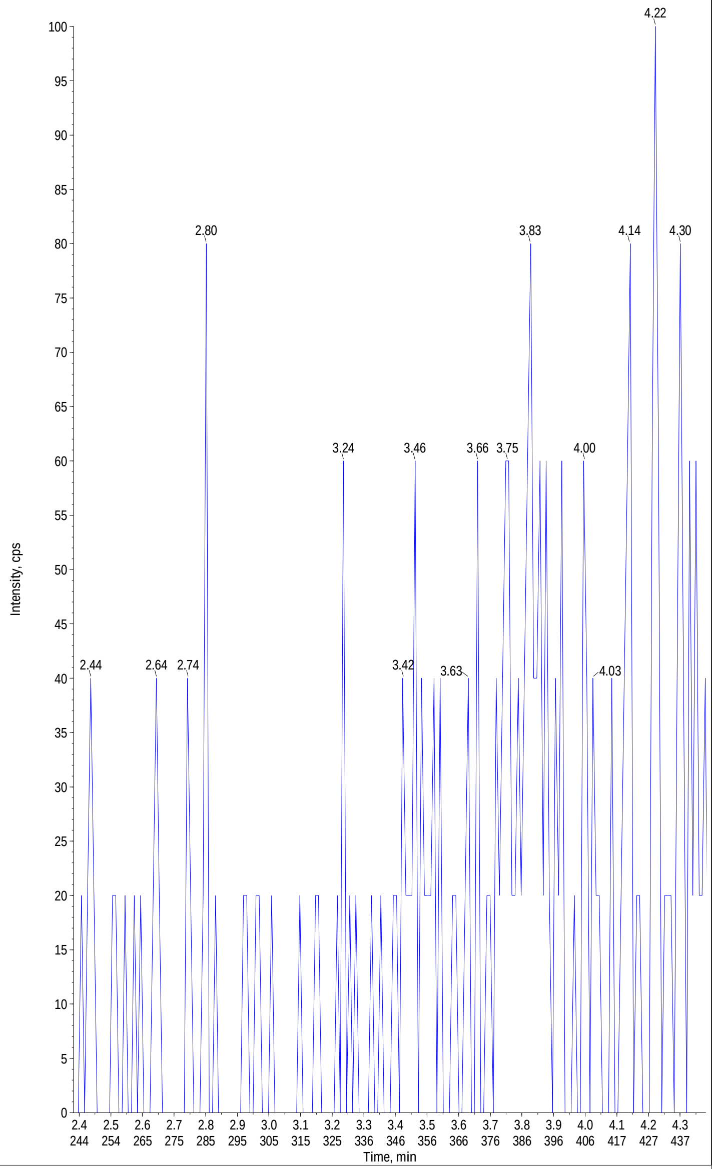

Sample Name: "KB6"   Sample ID: ""   File: "20220801-1.wiff"
Peak Name: "OHTOL"   Mass(es): "287.300/170.800 Da"
Comment: ""   Annotation: ""

**Sample Index:**	**9**
**Sample Type:**	**Unknown**
**Concentration:**	**N/A**
**Calculated Conc:**	**No Intercept**
**Acq. Date:**	**8/1/2022**
**Acq. Time:**	**11:58:04 AM**
**Modified:**	**No**

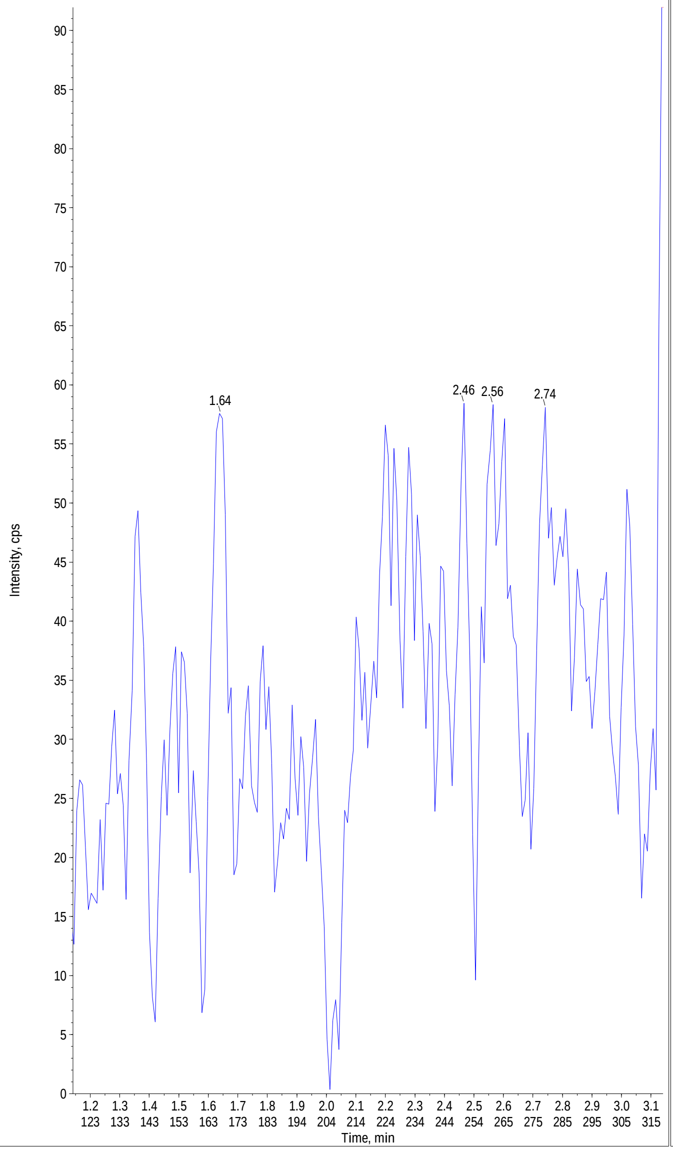

Sample Name: "KB6"   Sample ID: ""   File: "20220801-1.wiff"
Peak Name: "DXP(IS)"   Mass(es): "285.500/154.100 Da"
Comment: ""   Annotation: ""

**Sample Index:**	**9**
**Sample Type:**	**Unknown**
**Concentration:**	**1.00 ng/mL**
**Calculated Conc:**	**N/A**
**Acq. Date:**	**8/1/2022**
**Acq. Time:**	**11:58:04 AM**
**Modified:**	**No**

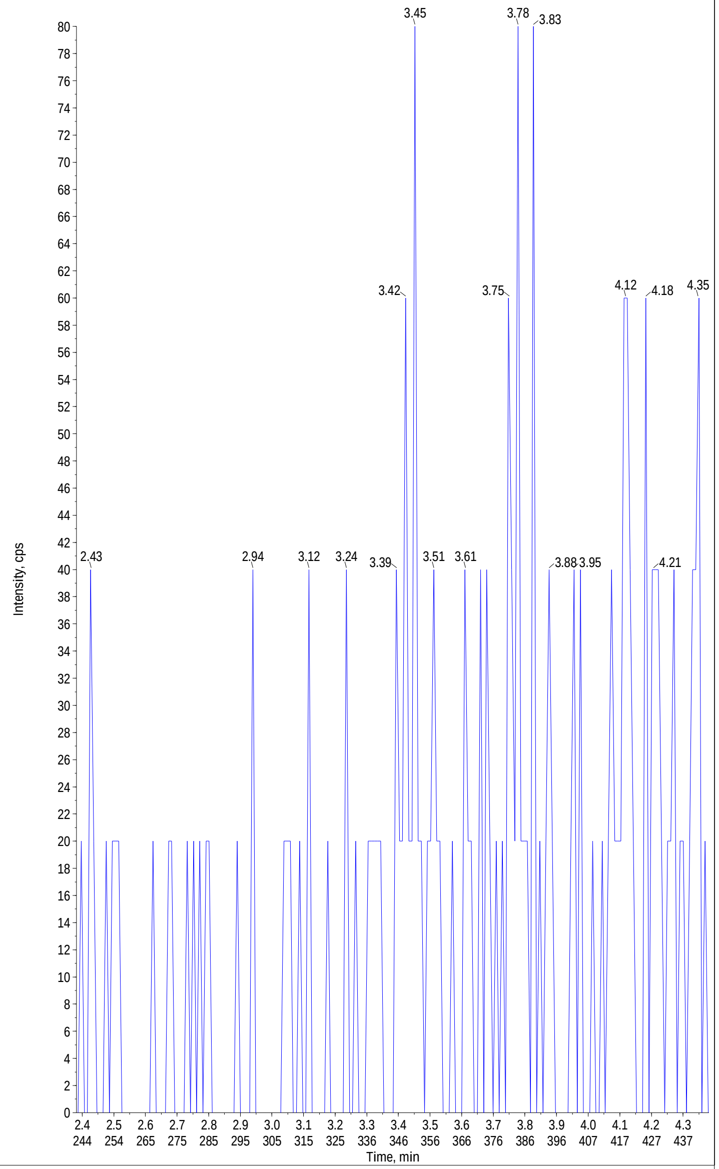

Sample Name: "KB+IS1"   Sample ID: ""   File: "20220801-1.wiff"
Peak Name: "OHTOL"   Mass(es): "287.300/170.800 Da"
Comment: ""   Annotation: ""

```
Sample Index:        10
Sample Type:         Unknown
Concentration:       N/A
Calculated Conc:     0.00     ng/mL
Acq. Date:           8/1/2022
Acq. Time:           12:03:04 PM

Modified:            No
```

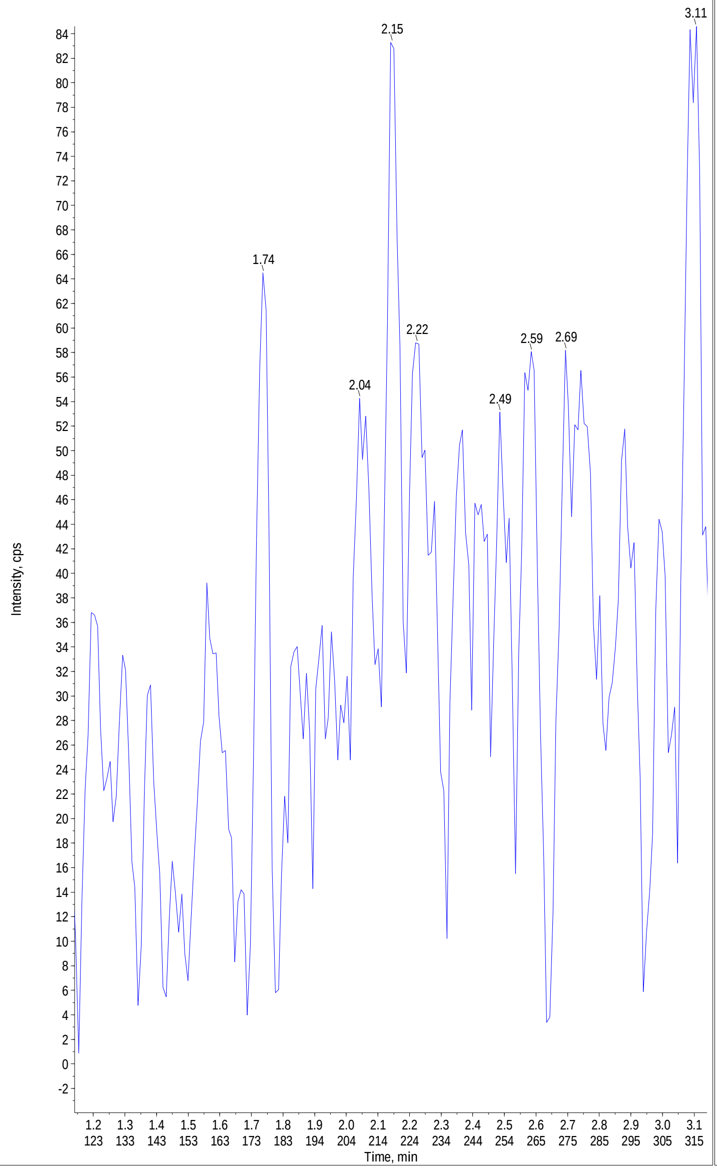

Sample Name: "KB+IS1"   Sample ID: ""   File: "20220801-1.wiff"
Peak Name: "DXP(IS)"   Mass(es): "285.500/154.100 Da"
Comment: ""   Annotation: ""

```
Sample Index:        10
Sample Type:         Unknown
Concentration:       1.00     ng/mL
Calculated Conc:     N/A
Acq. Date:           8/1/2022
Acq. Time:           12:03:04 PM

Modified:            No
Proc. Algorithm: Specify Parameters - MQ III
Noise Percentage:      50
Base. Sub. Window:     1.00    min
Peak-Split. Factor:    2
Report Largest Peak:   Yes
Min. Peak Height:      500.00   cps
Min. Peak Width:       0.00     sec
Smoothing Width:       0        points
RT Window:             30.0     sec
Expected RT:           3.38     min
Use Relative RT:       No

Int. Type:             Base To Base
Retention Time:        3.37     min
Area:                  1.68e+006   counts
Height:                2.70e+005   cps
Start Time:            3.25     min
End Time:              4.29     min
```

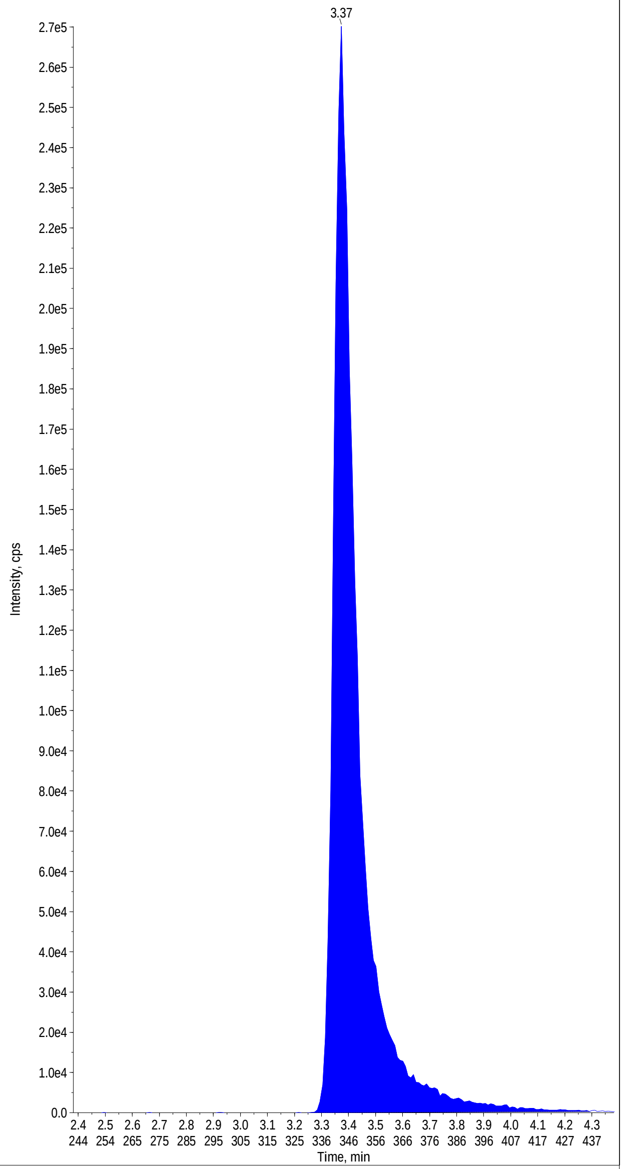

Sample Name: "KB+IS2" Sample ID: "" File: "20220801-1.wiff"
Peak Name: "OHTOL" Mass(es): "287.300/170.800 Da"
Comment: "" Annotation: ""

Sample Index: 11
Sample Type: Unknown
Concentration: N/A
Calculated Conc: 0.00 ng/mL
Acq. Date: 8/1/2022
Acq. Time: 12:08:03 PM

Modified: No

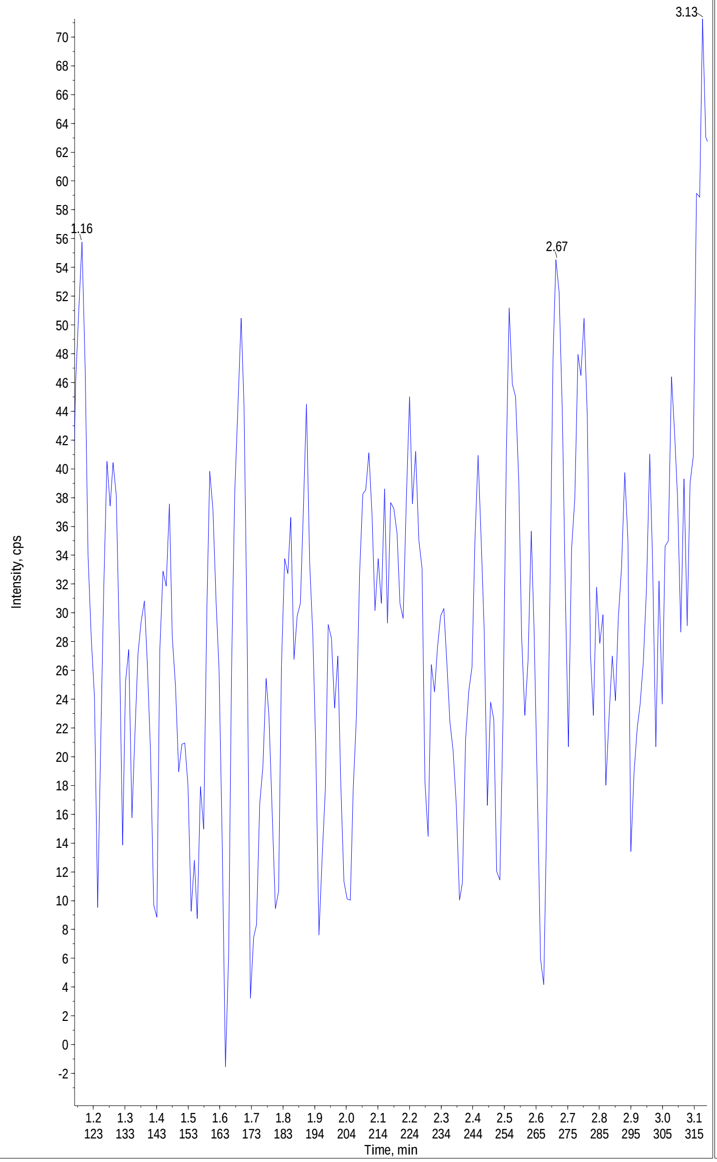

Sample Name: "KB+IS2" Sample ID: "" File: "20220801-1.wiff"
Peak Name: "DXP(IS)" Mass(es): "285.500/154.100 Da"
Comment: "" Annotation: ""

Sample Index: 11
Sample Type: Unknown
Concentration: 1.00 ng/mL
Calculated Conc: N/A
Acq. Date: 8/1/2022
Acq. Time: 12:08:03 PM

Modified: No
Proc. Algorithm: Specify Parameters - MQ III
Noise Percentage: 50
Base. Sub. Window: 1.00 min
Peak-Split. Factor: 2
Report Largest Peak: Yes
Min. Peak Height: 500.00 cps
Min. Peak Width: 0.00 sec
Smoothing Width: 0 points
RT Window: 30.0 sec
Expected RT: 3.38 min
Use Relative RT: No

Int. Type: Valley
Retention Time: 3.39 min
Area: 1.41e+006 counts
Height: 2.28e+005 cps
Start Time: 3.27 min
End Time: 4.13 min

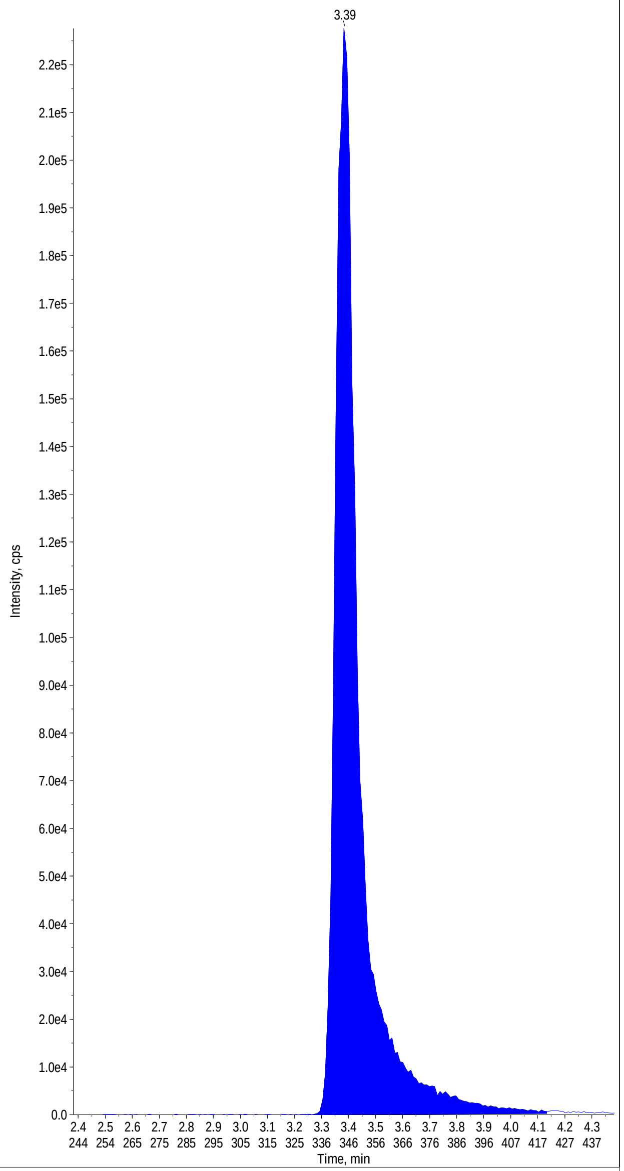

Sample Name: "KB+IS3" Sample ID: "" File: "20220801-1.wiff"
Peak Name: "OHTOL" Mass(es): "287.300/170.800 Da"
Comment: "" Annotation: ""

```
Sample Index:       12
Sample Type:      Unknown
Concentration:      N/A
Calculated Conc:    0.00    ng/mL
Acq. Date:        8/1/2022
Acq. Time:        12:13:08 PM

Modified:            No
```

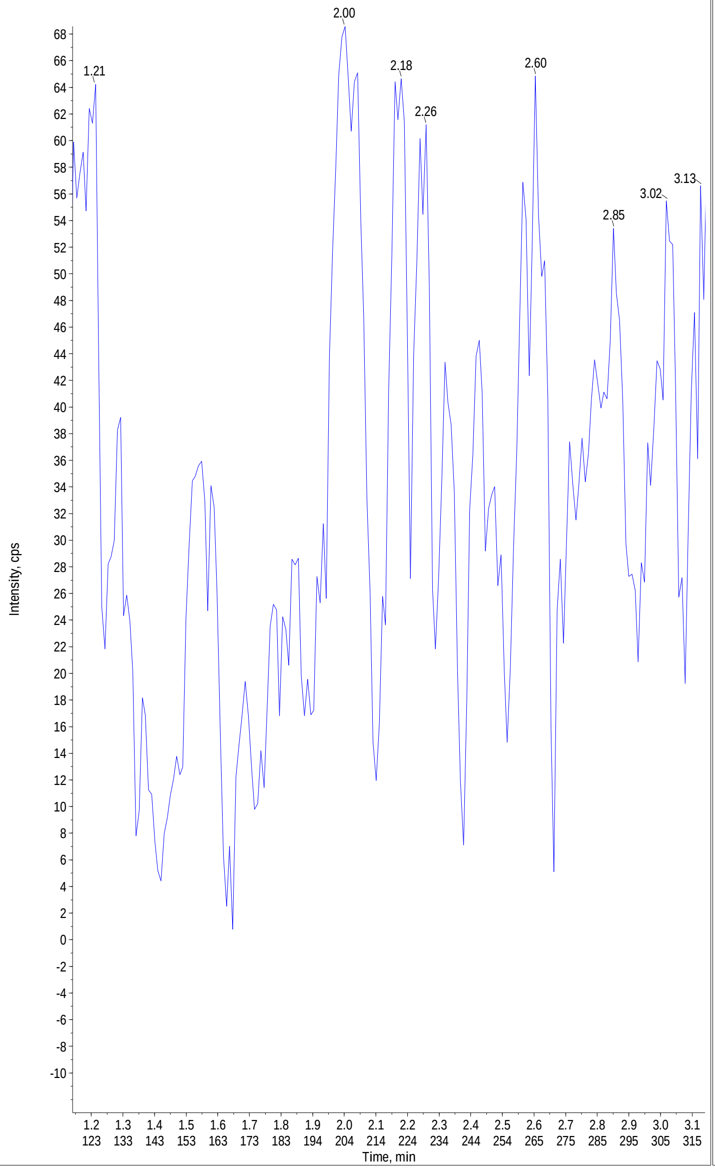

Sample Name: "KB+IS3" Sample ID: "" File: "20220801-1.wiff"
Peak Name: "DXP(IS)" Mass(es): "285.500/154.100 Da"
Comment: "" Annotation: ""

```
Sample Index:       12
Sample Type:      Unknown
Concentration:      1.00    ng/mL
Calculated Conc:    N/A
Acq. Date:        8/1/2022
Acq. Time:        12:13:08 PM

Modified:            No
Proc. Algorithm: Specify Parameters - MQ III
Noise Percentage:     50
Base. Sub. Window:    1.00   min
Peak-Split. Factor:   2
Report Largest Peak:   Yes
Min. Peak Height:    500.00   cps
Min. Peak Width:       0.00   sec
Smoothing Width:       0       points
RT Window:          30.0    sec
Expected RT:         3.38   min
Use Relative RT:     No

Int. Type:          Valley
Retention Time:      3.38   min
Area:          1.41e+006   counts
Height:          2.25e+005   cps
Start Time:          3.25   min
End Time:            4.26   min
```

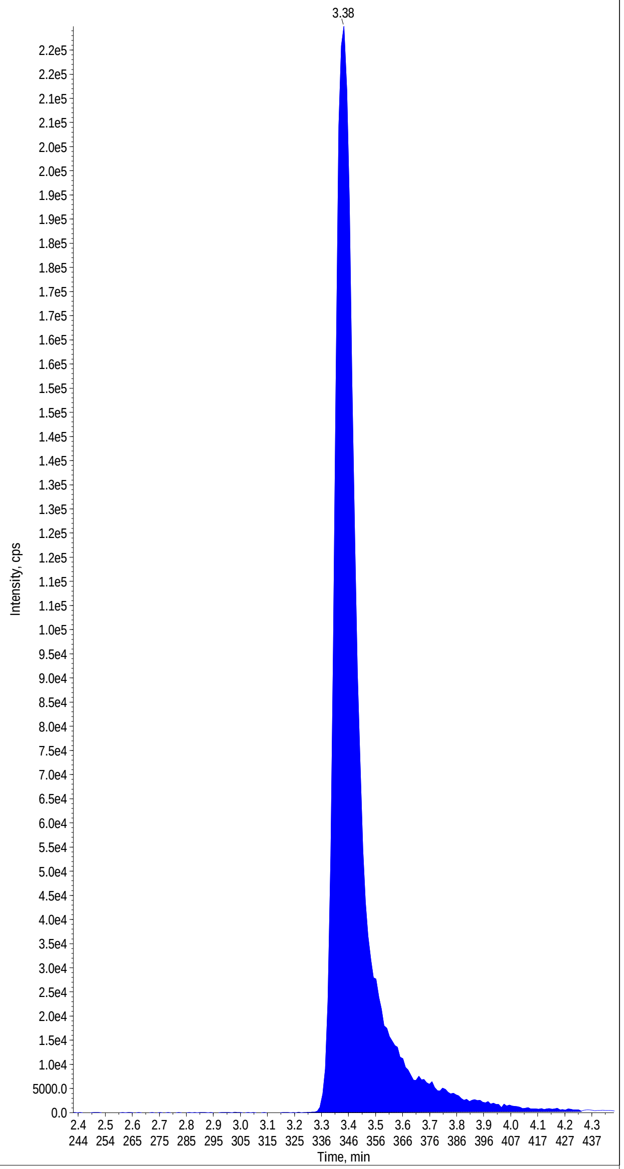

Sample Name: "KB+IS4"   Sample ID: ""   File: "20220801-1.wiff"
Peak Name: "OHTOL"   Mass(es): "287.300/170.800 Da"
Comment: ""   Annotation: ""

Sample Index:	13
Sample Type:	Unknown
Concentration:	N/A
Calculated Conc:	0.00 ng/mL
Acq. Date:	8/1/2022
Acq. Time:	12:18:06 PM
Modified:	No

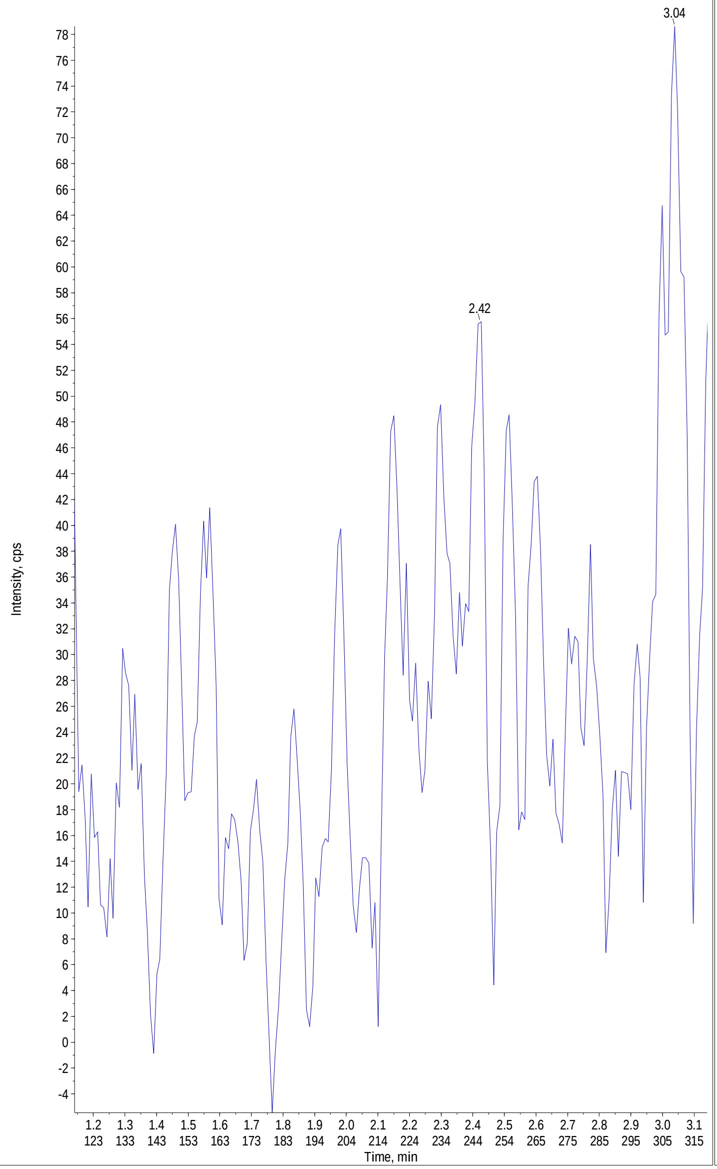

Sample Name: "KB+IS4"   Sample ID: ""   File: "20220801-1.wiff"
Peak Name: "DXP(IS)"   Mass(es): "285.500/154.100 Da"
Comment: ""   Annotation: ""

Sample Index:	13
Sample Type:	Unknown
Concentration:	1.00 ng/mL
Calculated Conc:	N/A
Acq. Date:	8/1/2022
Acq. Time:	12:18:06 PM
Modified:	No
Proc. Algorithm:	Specify Parameters - MQ III
Noise Percentage:	50
Base. Sub. Window:	1.00 min
Peak-Split. Factor:	2
Report Largest Peak:	Yes
Min. Peak Height:	500.00 cps
Min. Peak Width:	0.00 sec
Smoothing Width:	0 points
RT Window:	30.0 sec
Expected RT:	3.38 min
Use Relative RT:	No
Int. Type:	Valley
Retention Time:	3.38 min
Area:	1.39e+006 counts
Height:	2.22e+005 cps
Start Time:	3.26 min
End Time:	4.06 min

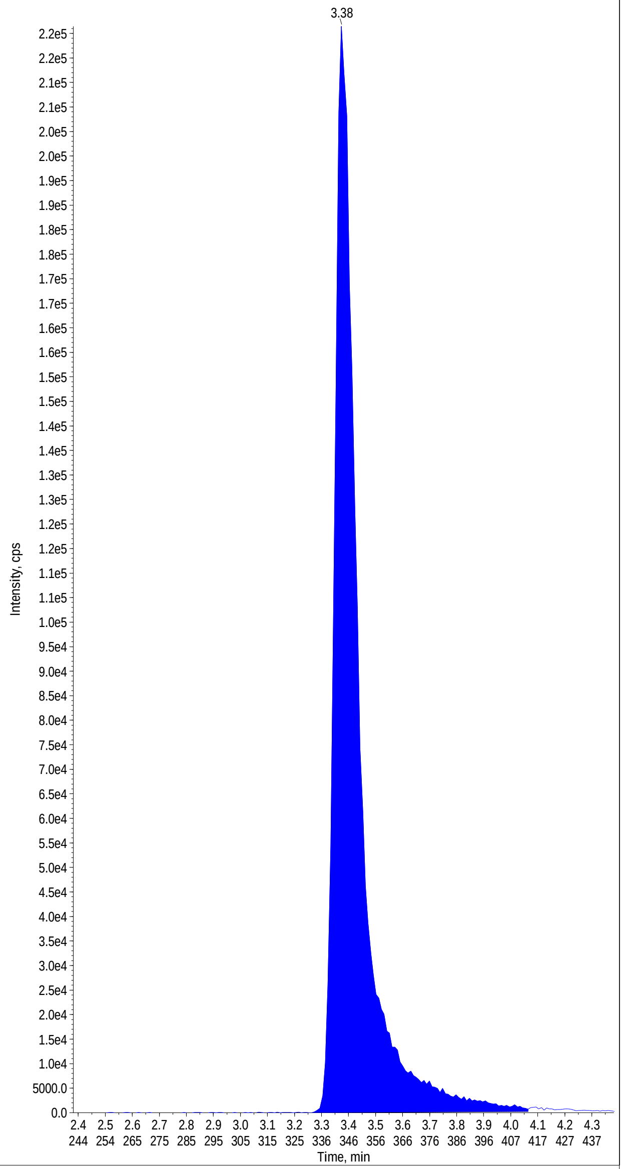

Sample Name: "KB+IS5"   Sample ID: ""   File: "20220801-1.wiff"
Peak Name: "OHTOL"   Mass(es): "287.300/170.800 Da"
Comment: ""   Annotation: ""

```
Sample Index:       14
Sample Type:        Unknown
Concentration:      N/A
Calculated Conc:    0.00    ng/mL
Acq. Date:          8/1/2022
Acq. Time:          12:23:11 PM

Modified:           No
```

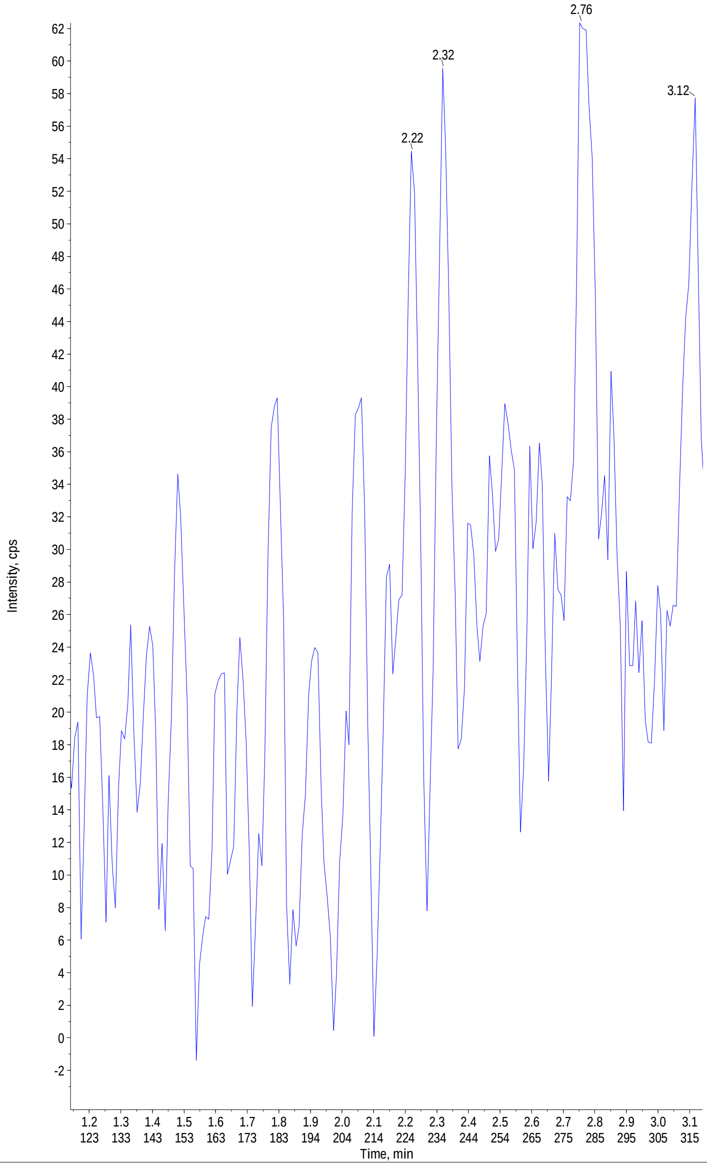

Sample Name: "KB+IS5"   Sample ID: ""   File: "20220801-1.wiff"
Peak Name: "DXP(IS)"   Mass(es): "285.500/154.100 Da"
Comment: ""   Annotation: ""

```
Sample Index:       14
Sample Type:        Unknown
Concentration:      1.00    ng/mL
Calculated Conc:    N/A
Acq. Date:          8/1/2022
Acq. Time:          12:23:11 PM

Modified:           No
Proc. Algorithm: Specify Parameters - MQ III
Noise Percentage:     50
Base. Sub. Window:    1.00   min
Peak-Split. Factor:   2
Report Largest Peak:   Yes
Min. Peak Height:     500.00   cps
Min. Peak Width:       0.00    sec
Smoothing Width:       0       points
RT Window:          30.0    sec
Expected RT:         3.38   min
Use Relative RT:     No

Int. Type:          Valley
Retention Time:      3.37   min
Area:          1.35e+006   counts
Height:          2.30e+005   cps
Start Time:          3.27   min
End Time:            3.96   min
```

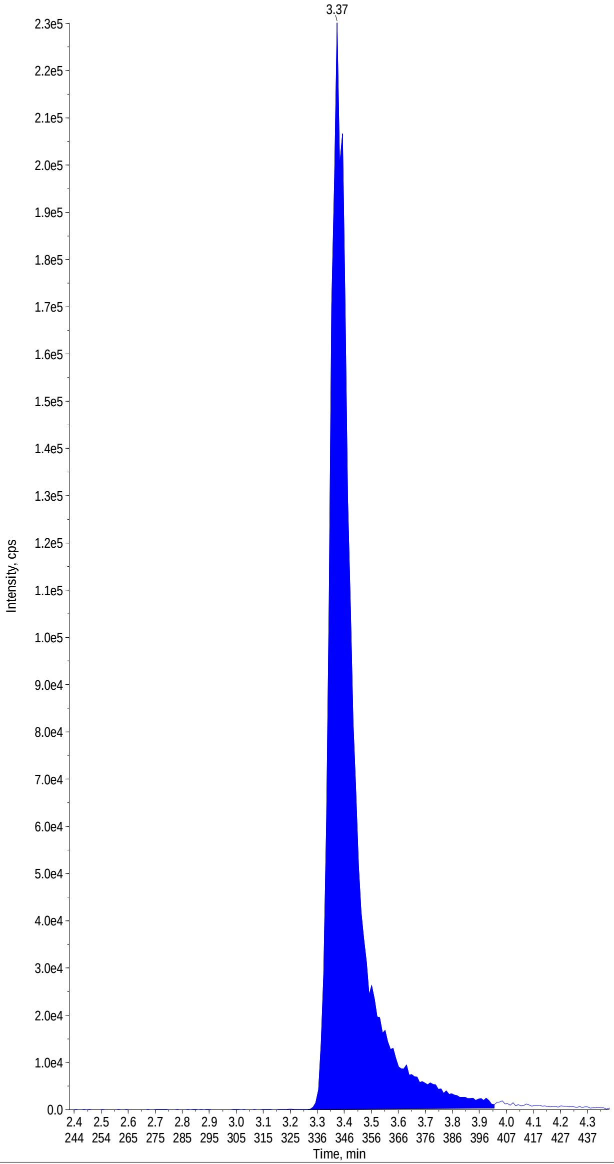

Sample Name: "KB+IS6" Sample ID: "" File: "20220801-1.wiff"
Peak Name: "OHTOL" Mass(es): "287.300/170.800 Da"
Comment: "" Annotation: ""

Sample Index: 15
Sample Type: Unknown
Concentration: N/A
Calculated Conc: 0.00 ng/mL
Acq. Date: 8/1/2022
Acq. Time: 12:28:13 PM

Modified: No

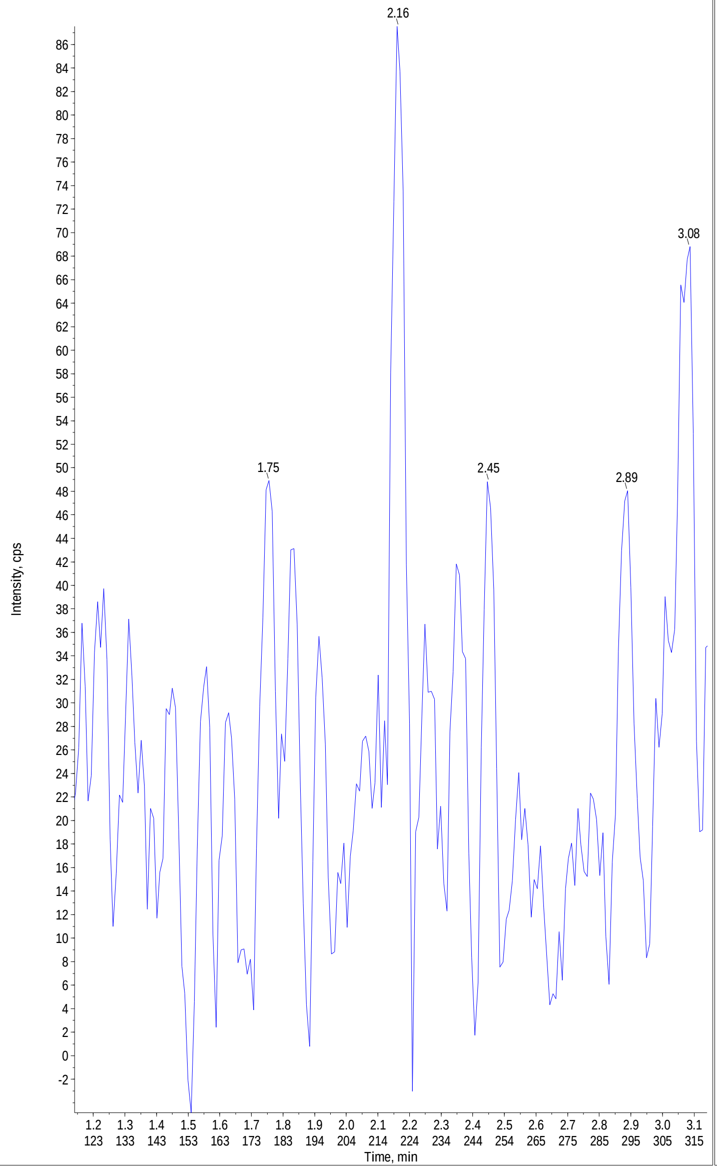

Sample Name: "KB+IS6" Sample ID: "" File: "20220801-1.wiff"
Peak Name: "DXP(IS)" Mass(es): "285.500/154.100 Da"
Comment: "" Annotation: ""

Sample Index: 15
Sample Type: Unknown
Concentration: 1.00 ng/mL
Calculated Conc: N/A
Acq. Date: 8/1/2022
Acq. Time: 12:28:13 PM

Modified: No
Proc. Algorithm: Specify Parameters - MQ III
Noise Percentage: 50
Base. Sub. Window: 1.00 min
Peak-Split. Factor: 2
Report Largest Peak: Yes
Min. Peak Height: 500.00 cps
Min. Peak Width: 0.00 sec
Smoothing Width: 0 points
RT Window: 30.0 sec
Expected RT: 3.38 min
Use Relative RT: No

Int. Type: Base To Base
Retention Time: 3.39 min
Area: 1.36e+006 counts
Height: 2.04e+005 cps
Start Time: 3.27 min
End Time: 4.28 min

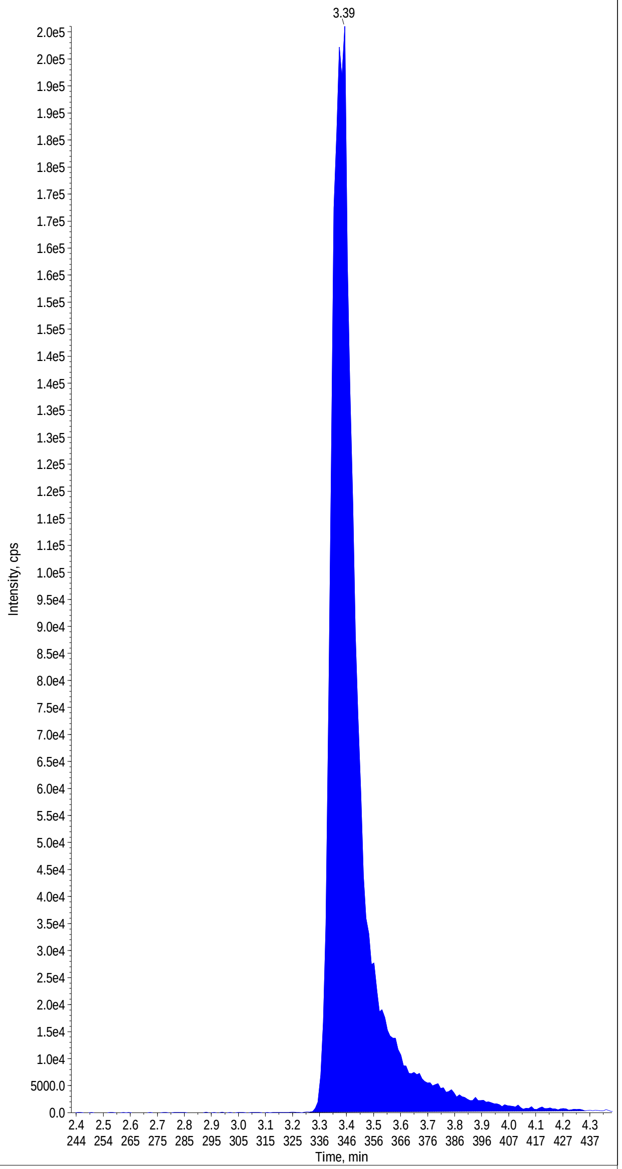

Sample Name: "SPIKE SAMPLE1"   Sample ID: ""   File: "20220801-1.wiff"
Peak Name: "OHTOL"   Mass(es): "287.300/170.800 Da"
Comment: ""   Annotation: ""

Sample Index: 43
Sample Type: Unknown
Concentration: N/A
Calculated Conc: No Intercept
Acq. Date: 8/1/2022
Acq. Time: 6:55:36 PM

Modified: No
Proc. Algorithm: Specify Parameters - MQ III
Noise Percentage: 50
Base. Sub. Window: 1.00 min
Peak-Split. Factor: 2
Report Largest Peak: Yes
Min. Peak Height: 300.00 cps
Min. Peak Width: 0.00 sec
Smoothing Width: 9 points
RT Window: 30.0 sec
Expected RT: 2.14 min
Use Relative RT: No

Int. Type: Base To Base
Retention Time: 2.18 min
Area: 8.90e+003 counts
Height: 1.43e+003 cps
Start Time: 2.03 min
End Time: 2.44 min

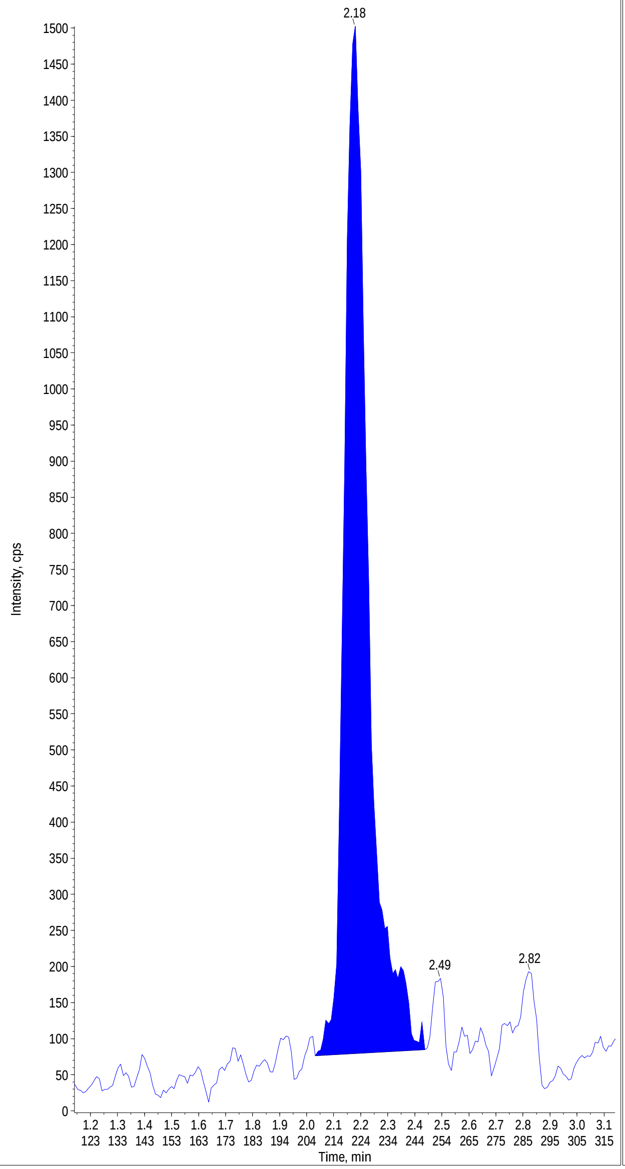

Sample Name: "SPIKE SAMPLE1"   Sample ID: ""   File: "20220801-1.wiff"
Peak Name: "DXP(IS)"   Mass(es): "285.500/154.100 Da"
Comment: ""   Annotation: ""

Sample Index: 43
Sample Type: Unknown
Concentration: 1.00 ng/mL
Calculated Conc: N/A
Acq. Date: 8/1/2022
Acq. Time: 6:55:36 PM

Modified: No

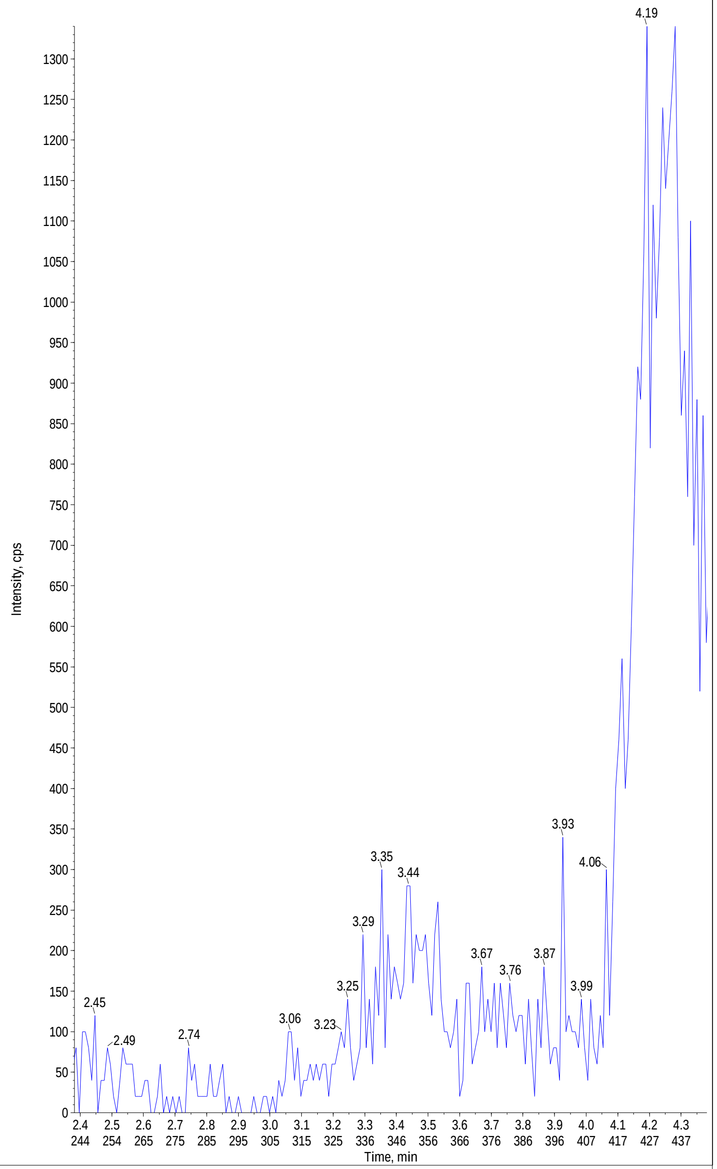

Sample Name: "SPIKE SAMPLE2" Sample ID: "" File: "20220801-1.wiff"
Peak Name: "OHTOL" Mass(es): "287.300/170.800 Da"
Comment: "" Annotation: ""

```
Sample Index:       44
Sample Type:        Unknown
Concentration:      N/A
Calculated Conc: No Intercept
Acq. Date:          8/1/2022
Acq. Time:          7:00:38 PM

Modified:           No
Proc. Algorithm: Specify Parameters - MQ III
Noise Percentage:   50
Base. Sub. Window:  1.00   min
Peak-Split. Factor: 2
Report Largest Peak: Yes
Min. Peak Height:   300.00  cps
Min. Peak Width:    0.00    sec
Smoothing Width:    9       points
RT Window:          30.0    sec
Expected RT:        2.14    min
Use Relative RT:    No

Int. Type:          Valley
Retention Time:     2.17    min
Area:               9.77e+003   counts
Height:             1.44e+003   cps
Start Time:         2.08    min
End Time:           2.49    min
```

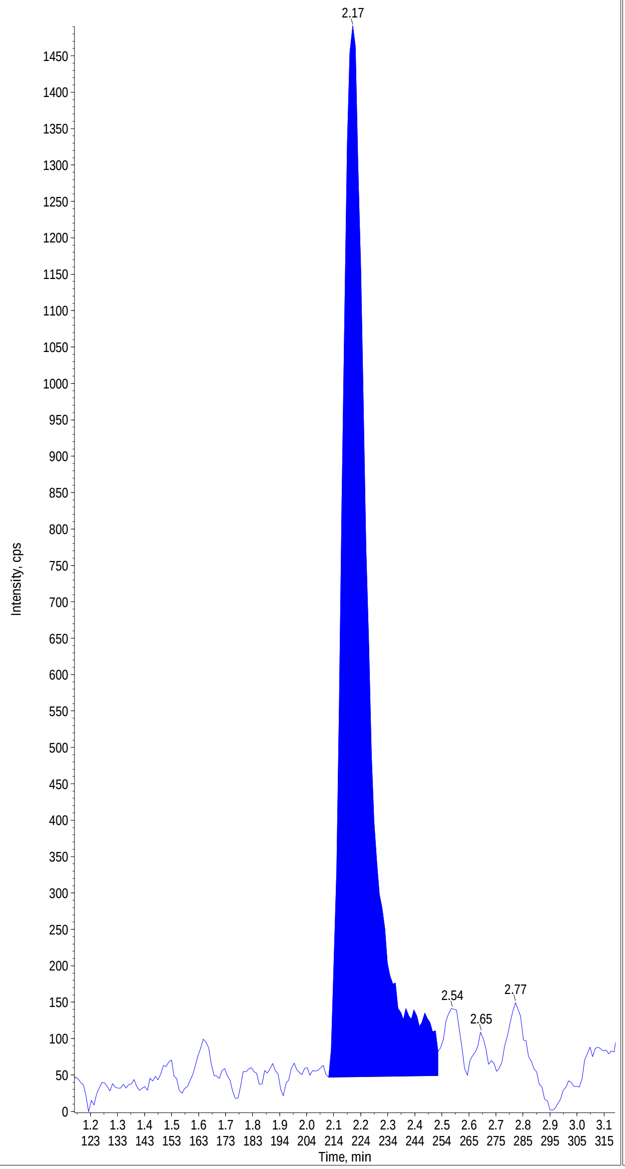

Sample Name: "SPIKE SAMPLE2" Sample ID: "" File: "20220801-1.wiff"
Peak Name: "DXP(IS)" Mass(es): "285.500/154.100 Da"
Comment: "" Annotation: ""

```
Sample Index:       44
Sample Type:        Unknown
Concentration:      1.00    ng/mL
Calculated Conc:    N/A
Acq. Date:          8/1/2022
Acq. Time:          7:00:38 PM

Modified:           No
```

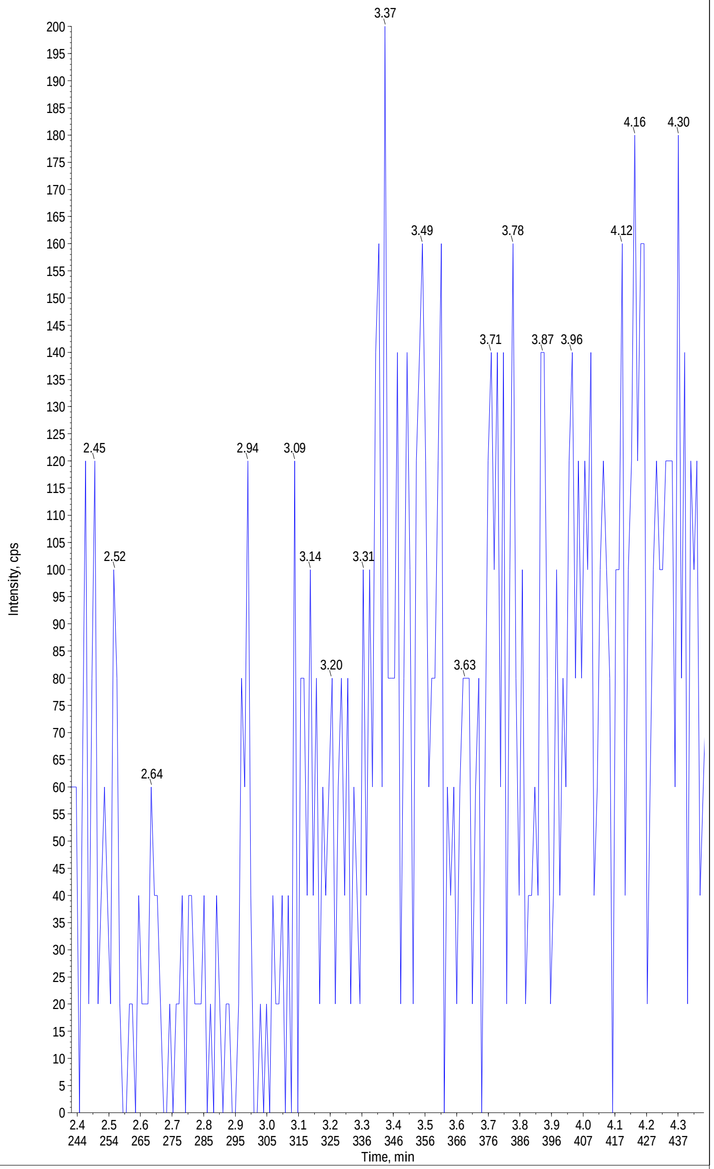

Sample Name: "SPIKE SAMPLE3"   Sample ID: ""   File: "20220801-1.wiff"
Peak Name: "OHTOL"   Mass(es): "287.300/170.800 Da"
Comment: ""   Annotation: ""

Sample Index: 45
Sample Type: Unknown
Concentration: N/A
Calculated Conc: No Intercept
Acq. Date: 8/1/2022
Acq. Time: 7:05:39 PM

Modified: No
Proc. Algorithm: Specify Parameters - MQ III
Noise Percentage: 50
Base. Sub. Window: 1.00 min
Peak-Split. Factor: 2
Report Largest Peak: Yes
Min. Peak Height: 300.00 cps
Min. Peak Width: 0.00 sec
Smoothing Width: 9 points
RT Window: 30.0 sec
Expected RT: 2.14 min
Use Relative RT: No

Int. Type: Valley
Retention Time: 2.17 min
Area: 9.23e+003 counts
Height: 1.52e+003 cps
Start Time: 2.06 min
End Time: 2.40 min

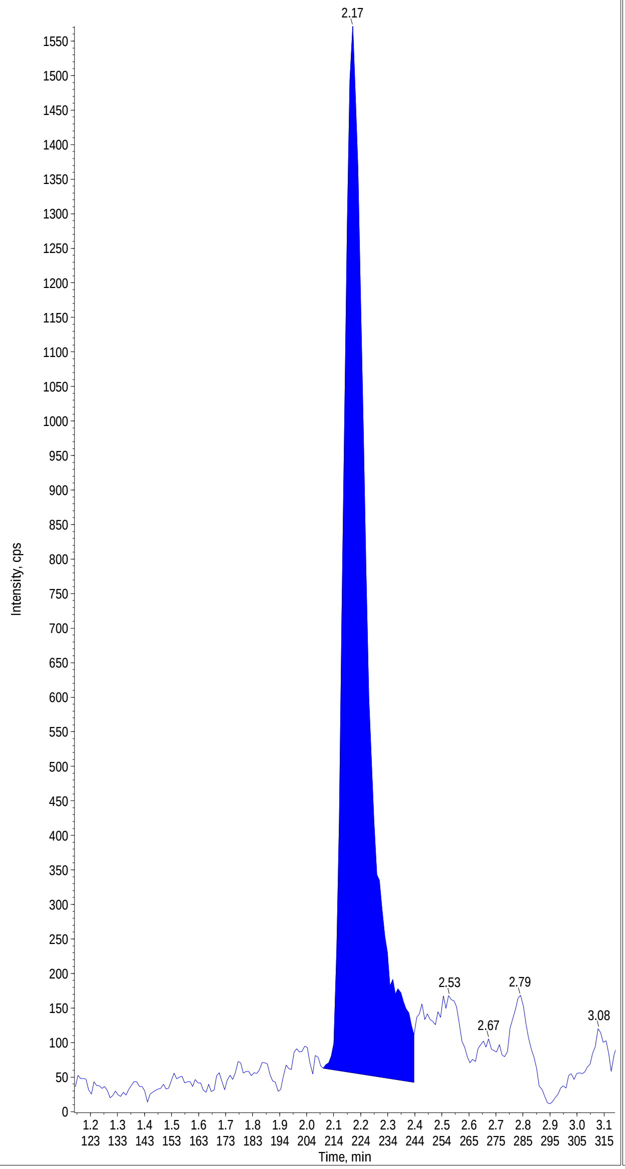

Sample Name: "SPIKE SAMPLE3"   Sample ID: ""   File: "20220801-1.wiff"
Peak Name: "DXP(IS)"   Mass(es): "285.500/154.100 Da"
Comment: ""   Annotation: ""

Sample Index: 45
Sample Type: Unknown
Concentration: 1.00 ng/mL
Calculated Conc: N/A
Acq. Date: 8/1/2022
Acq. Time: 7:05:39 PM

Modified: No

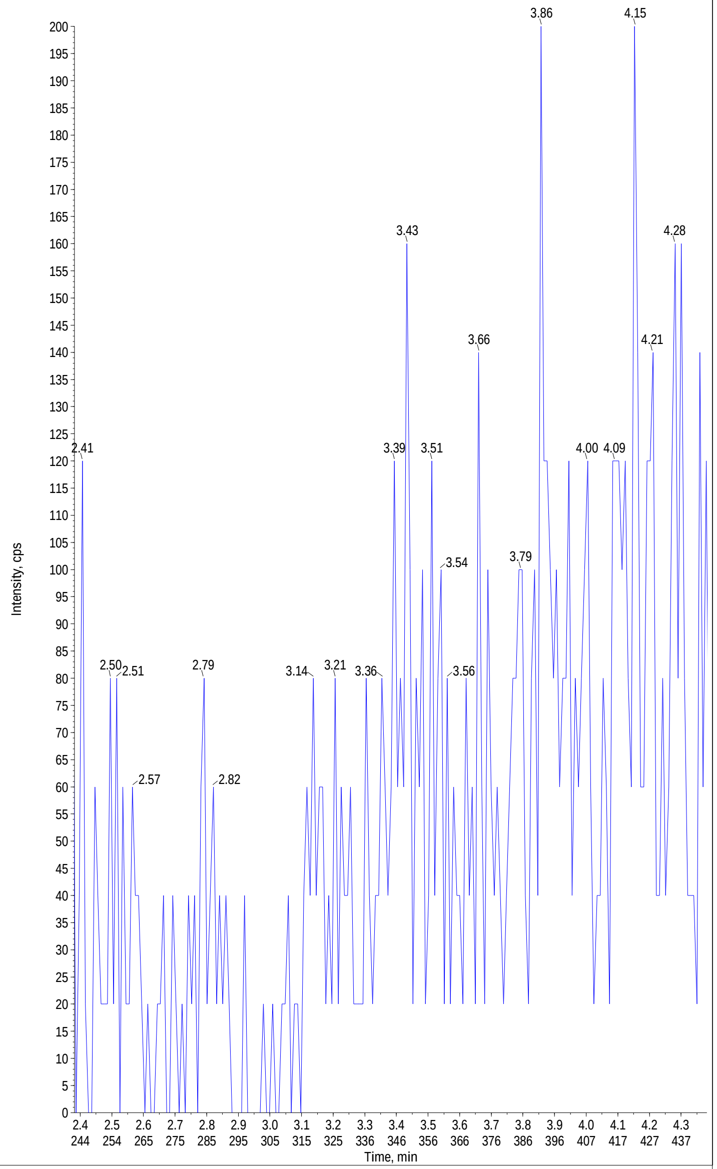

Sample Name: "SPIKE SAMPLE4" Sample ID: "" File: "20220801-1.wiff"
Peak Name: "OHTOL" Mass(es): "287.300/170.800 Da"
Comment: "" Annotation: ""

```
Sample Index:      46
Sample Type:      Unknown
Concentration:      N/A
Calculated Conc: No Intercept
Acq. Date:        8/1/2022
Acq. Time:        7:10:44 PM

Modified:            No
Proc. Algorithm: Specify Parameters - MQ III
Noise Percentage:     50
Base. Sub. Window:    1.00   min
Peak-Split. Factor:   2
Report Largest Peak:   Yes
Min. Peak Height:    300.00   cps
Min. Peak Width:       0.00   sec
Smoothing Width:       9       points
RT Window:          30.0    sec
Expected RT:         2.14   min
Use Relative RT:     No

Int. Type:         Valley
Retention Time:      2.17   min
Area:          9.29e+003   counts
Height:          1.54e+003  cps
Start Time:          2.07   min
End Time:            2.37   min
```

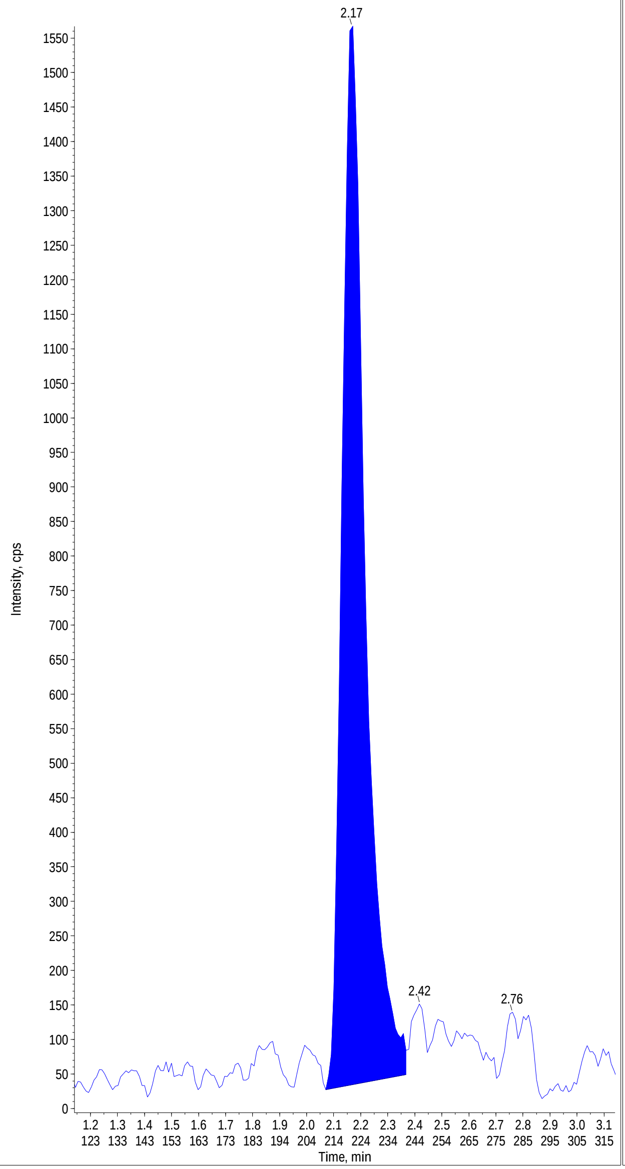

Sample Name: "SPIKE SAMPLE4" Sample ID: "" File: "20220801-1.wiff"
Peak Name: "DXP(IS)" Mass(es): "285.500/154.100 Da"
Comment: "" Annotation: ""

```
Sample Index:      46
Sample Type:      Unknown
Concentration:      1.00    ng/mL
Calculated Conc:    N/A
Acq. Date:        8/1/2022
Acq. Time:        7:10:44 PM

Modified:            No
```

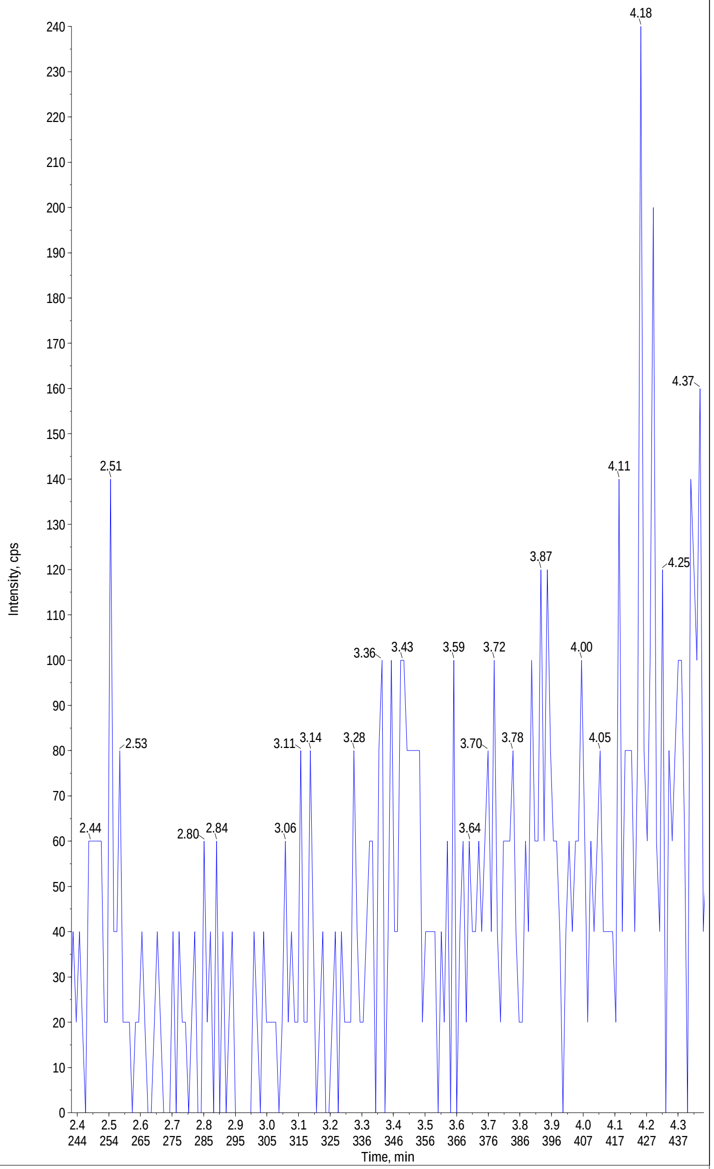

Sample Name: "SPIKE SAMPLE5" Sample ID: "" File: "20220801-1.wiff"
Peak Name: "OHTOL" Mass(es): "287.300/170.800 Da"
Comment: "" Annotation: ""

```
Sample Index:       47
Sample Type:        Unknown
Concentration:      N/A
Calculated Conc:    No Intercept
Acq. Date:          8/1/2022
Acq. Time:          7:15:45 PM

Modified:           No
Proc. Algorithm:    Specify Parameters - MQ III
Noise Percentage:   50
Base. Sub. Window:  1.00   min
Peak-Split. Factor: 2
Report Largest Peak: Yes
Min. Peak Height:   300.00  cps
Min. Peak Width:    0.00    sec
Smoothing Width:    9       points
RT Window:          30.0    sec
Expected RT:        2.14    min
Use Relative RT:    No

Int. Type:          Base To Base
Retention Time:     2.17    min
Area:               9.26e+003  counts
Height:             1.49e+003  cps
Start Time:         2.05    min
End Time:           2.44    min
```

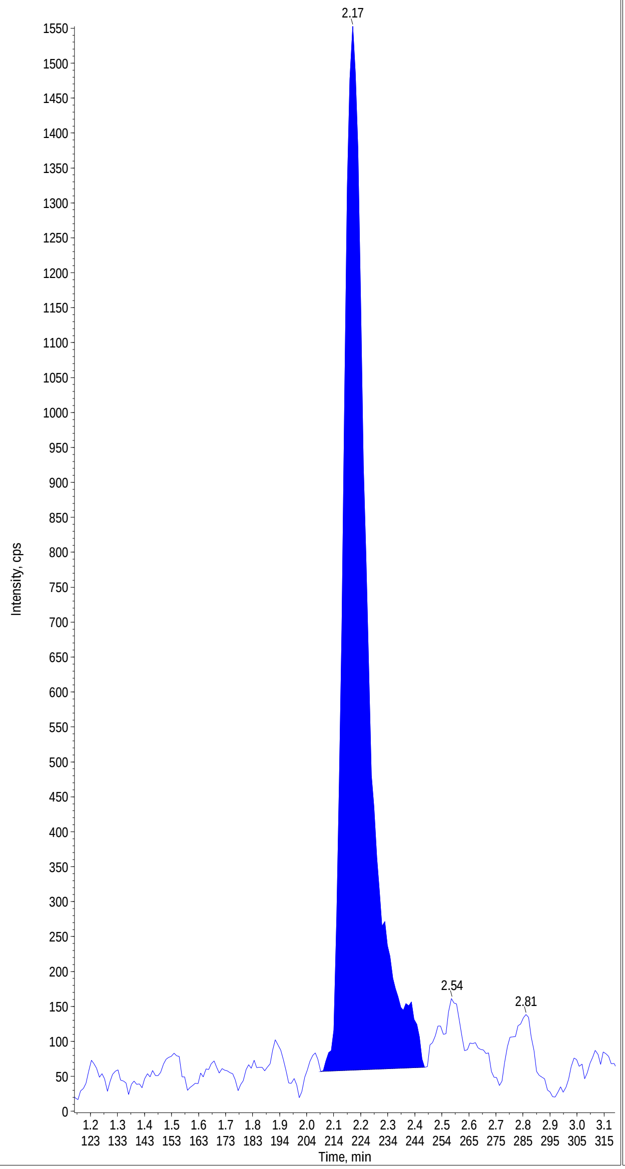

Sample Name: "SPIKE SAMPLE5" Sample ID: "" File: "20220801-1.wiff"
Peak Name: "DXP(IS)" Mass(es): "285.500/154.100 Da"
Comment: "" Annotation: ""

```
Sample Index:       47
Sample Type:        Unknown
Concentration:      1.00    ng/mL
Calculated Conc:    N/A
Acq. Date:          8/1/2022
Acq. Time:          7:15:45 PM

Modified:           No
```

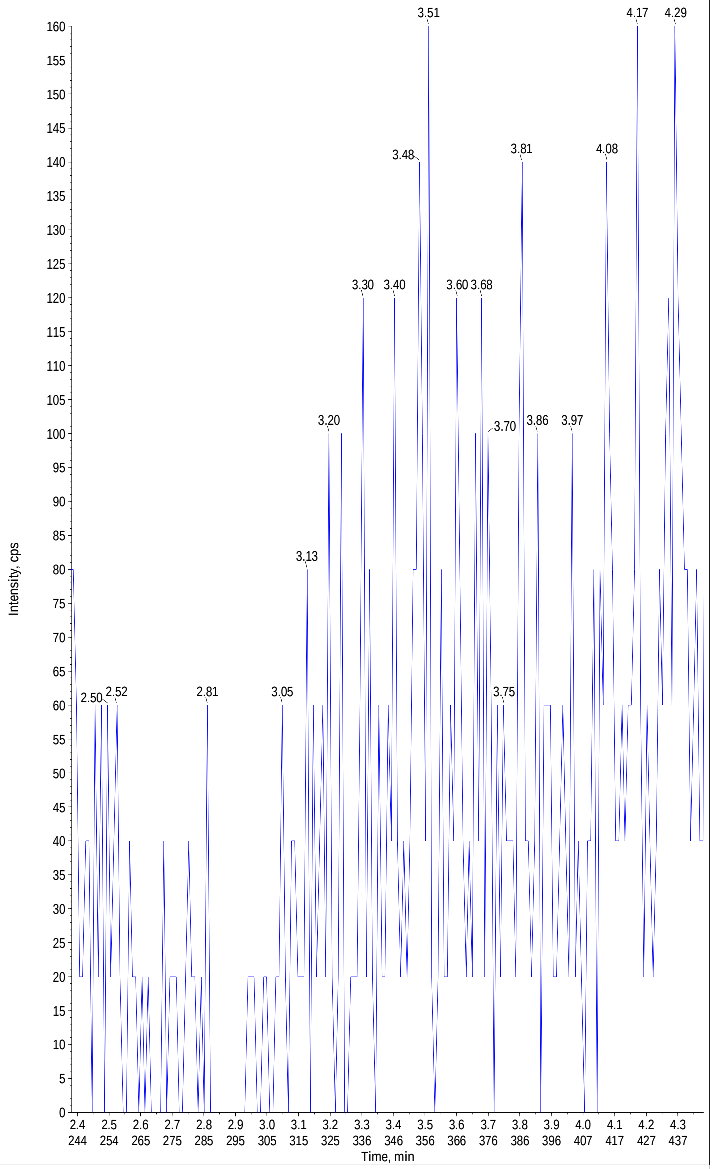

Sample Name: "SPIKE SAMPLE6" Sample ID: "" File: "20220801-1.wiff"
Peak Name: "OHTOL" Mass(es): "287.300/170.800 Da"
Comment: "" Annotation: ""

Sample Index: 48
Sample Type: Unknown
Concentration: N/A
Calculated Conc: No Intercept
Acq. Date: 8/1/2022
Acq. Time: 7:20:49 PM

Modified: No
Proc. Algorithm: Specify Parameters - MQ III
Noise Percentage: 50
Base. Sub. Window: 1.00 min
Peak-Split. Factor: 2
Report Largest Peak: Yes
Min. Peak Height: 300.00 cps
Min. Peak Width: 0.00 sec
Smoothing Width: 9 points
RT Window: 30.0 sec
Expected RT: 2.14 min
Use Relative RT: No

Int. Type: Valley
Retention Time: 2.17 min
Area: 8.85e+003 counts
Height: 1.49e+003 cps
Start Time: 2.04 min
End Time: 2.42 min

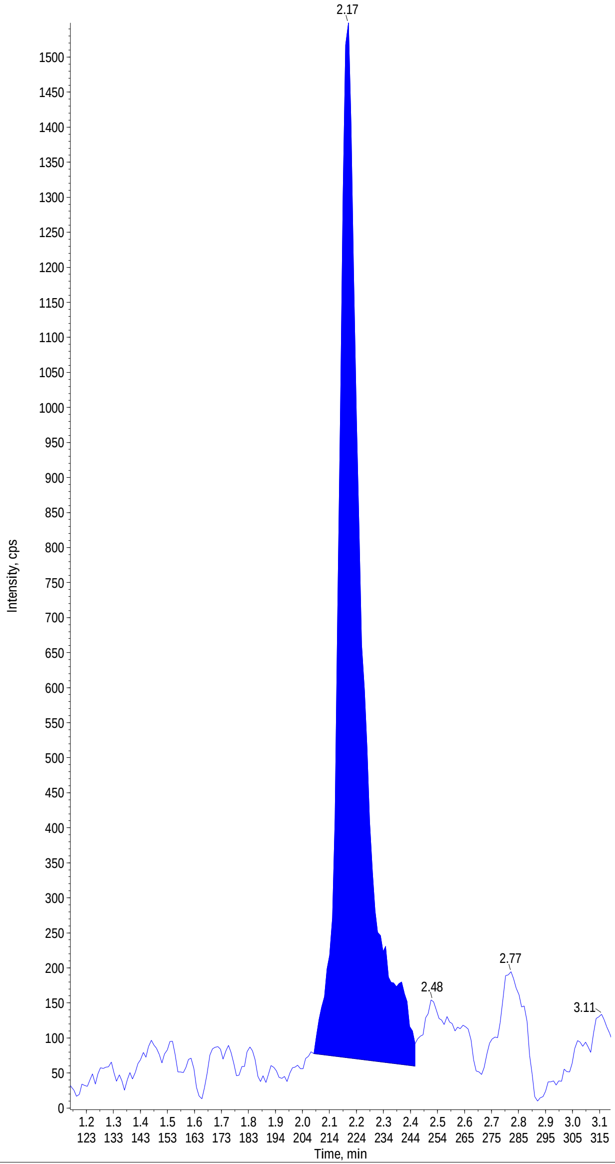

Sample Name: "SPIKE SAMPLE6" Sample ID: "" File: "20220801-1.wiff"
Peak Name: "DXP(IS)" Mass(es): "285.500/154.100 Da"
Comment: "" Annotation: ""

Sample Index: 48
Sample Type: Unknown
Concentration: 1.00 ng/mL
Calculated Conc: N/A
Acq. Date: 8/1/2022
Acq. Time: 7:20:49 PM

Modified: No

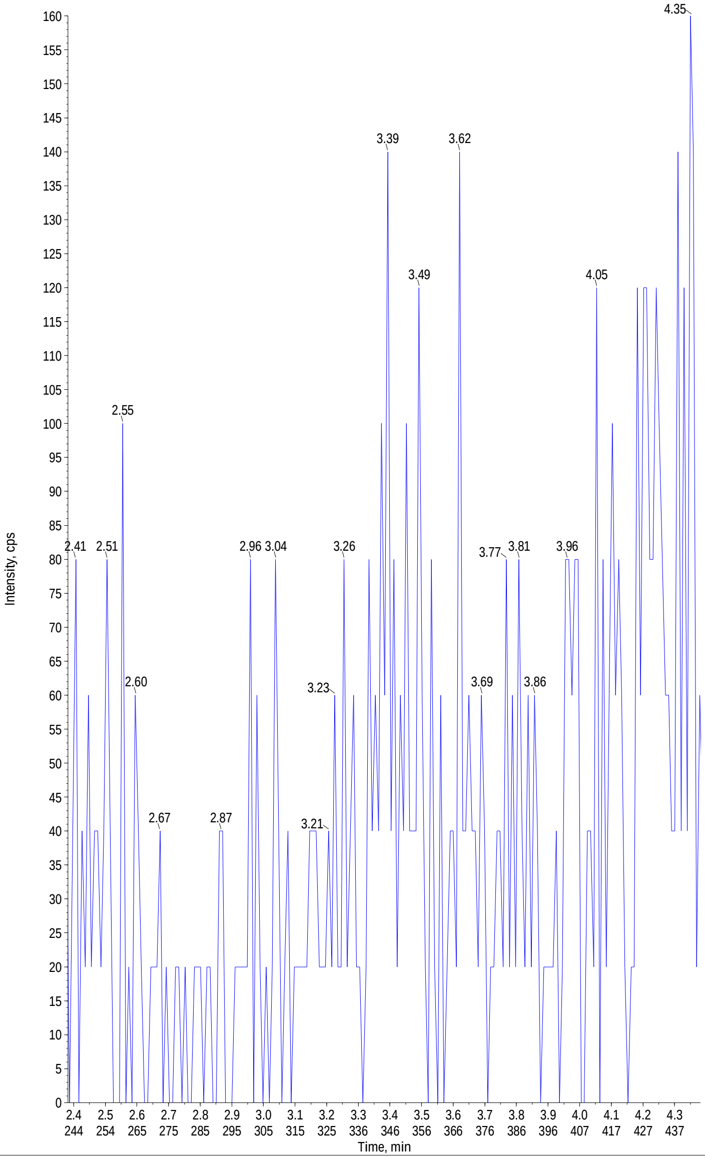

Sample Name: "LLOQ1" Sample ID: "" File: "20220801-1.wiff"
Peak Name: "OHTOL" Mass(es): "287.300/170.800 Da"
Comment: "" Annotation: ""

```
Sample Index:       36
Sample Type:        Unknown
Concentration:      N/A
Calculated Conc:    0.00    ng/mL
Acq. Date:          8/1/2022
Acq. Time:          3:38:38 PM

Modified:           No
Proc. Algorithm: Specify Parameters - MQ III
Noise Percentage:     50
Base. Sub. Window:    1.00   min
Peak-Split. Factor:   2
Report Largest Peak:   Yes
Min. Peak Height:    300.00   cps
Min. Peak Width:       0.00   sec
Smoothing Width:       9       points
RT Window:          30.0     sec
Expected RT:         2.14   min
Use Relative RT:     No

Int. Type:         Base To Base
Retention Time:      2.15   min
Area:            9.36e+004   counts
Height:           1.52e+004  cps
Start Time:          2.04   min
End Time:            2.57   min
```

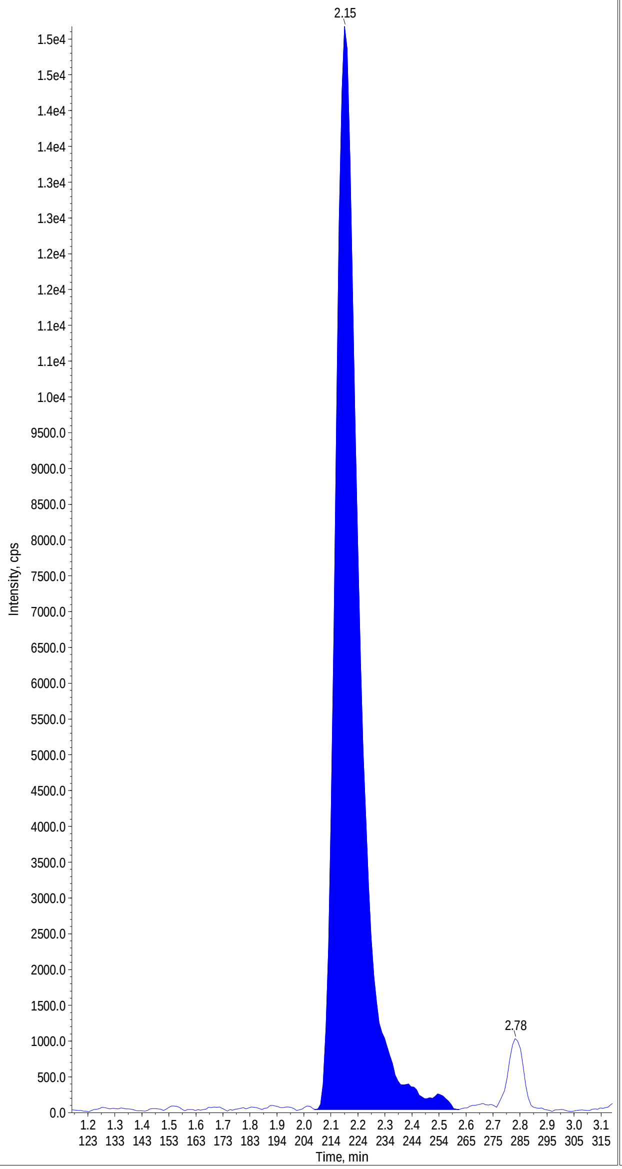

Sample Name: "LLOQ1" Sample ID: "" File: "20220801-1.wiff"
Peak Name: "DXP(IS)" Mass(es): "285.500/154.100 Da"
Comment: "" Annotation: ""

```
Sample Index:       36
Sample Type:        Unknown
Concentration:      1.00    ng/mL
Calculated Conc:    N/A
Acq. Date:          8/1/2022
Acq. Time:          3:38:38 PM

Modified:           No
Proc. Algorithm: Specify Parameters - MQ III
Noise Percentage:     50
Base. Sub. Window:    1.00   min
Peak-Split. Factor:   2
Report Largest Peak:   Yes
Min. Peak Height:    500.00   cps
Min. Peak Width:       0.00   sec
Smoothing Width:       0       points
RT Window:          30.0     sec
Expected RT:         3.38   min
Use Relative RT:     No

Int. Type:         Base To Base
Retention Time:      3.38   min
Area:            1.20e+007   counts
Height:           1.90e+006  cps
Start Time:          3.25   min
End Time:            4.32   min
```

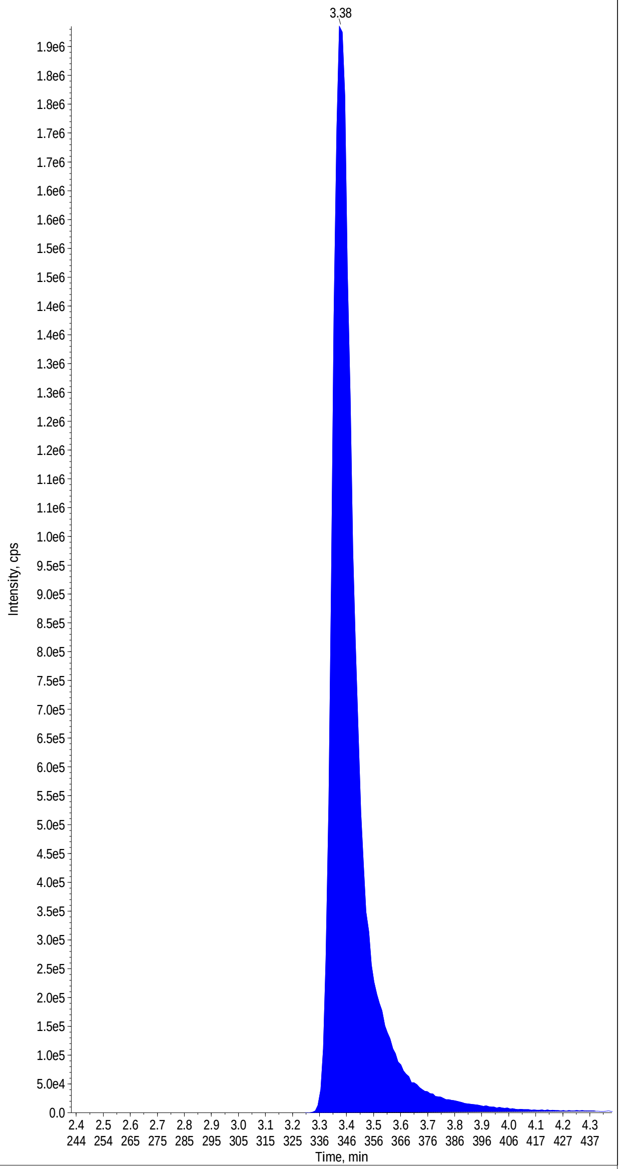

Sample Name: "LLOQ2" Sample ID: "" File: "20220801-1.wiff"
Peak Name: "OHTOL" Mass(es): "287.300/170.800 Da"
Comment: "" Annotation: ""

Sample Index:	37	
Sample Type:	Unknown	
Concentration:	N/A	
Calculated Conc:	0.00	ng/mL
Acq. Date:	8/1/2022	
Acq. Time:	3:43:40 PM	
Modified:	No	
Proc. Algorithm:	Specify Parameters - MQ III	
Noise Percentage:	50	
Base. Sub. Window:	1.00	min
Peak-Split. Factor:	2	
Report Largest Peak:	Yes	
Min. Peak Height:	300.00	cps
Min. Peak Width:	0.00	sec
Smoothing Width:	9	points
RT Window:	30.0	sec
Expected RT:	2.14	min
Use Relative RT:	No	
Int. Type:	Base To Base	
Retention Time:	2.15	min
Area:	9.19e+004	counts
Height:	1.46e+004	cps
Start Time:	2.05	min
End Time:	2.57	min

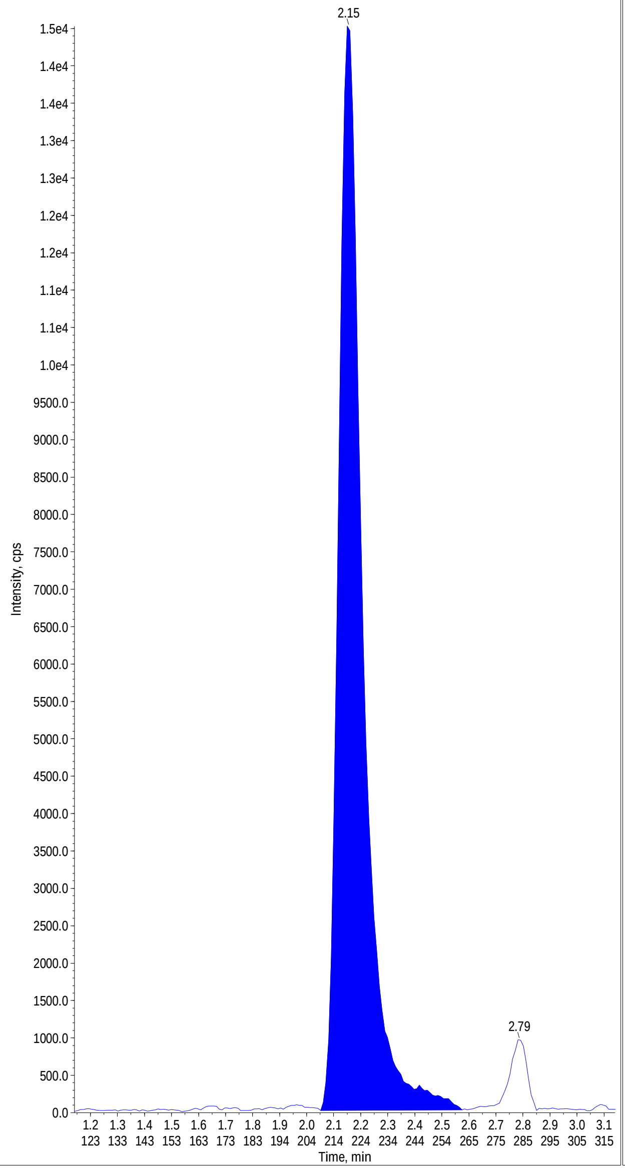

Sample Name: "LLOQ2" Sample ID: "" File: "20220801-1.wiff"
Peak Name: "DXP(IS)" Mass(es): "285.500/154.100 Da"
Comment: "" Annotation: ""

Sample Index:	37	
Sample Type:	Unknown	
Concentration:	1.00	ng/mL
Calculated Conc:	N/A	
Acq. Date:	8/1/2022	
Acq. Time:	3:43:40 PM	
Modified:	No	
Proc. Algorithm:	Specify Parameters - MQ III	
Noise Percentage:	50	
Base. Sub. Window:	1.00	min
Peak-Split. Factor:	2	
Report Largest Peak:	Yes	
Min. Peak Height:	500.00	cps
Min. Peak Width:	0.00	sec
Smoothing Width:	0	points
RT Window:	30.0	sec
Expected RT:	3.38	min
Use Relative RT:	No	
Int. Type:	Base To Base	
Retention Time:	3.38	min
Area:	1.03e+007	counts
Height:	1.74e+006	cps
Start Time:	3.26	min
End Time:	4.34	min

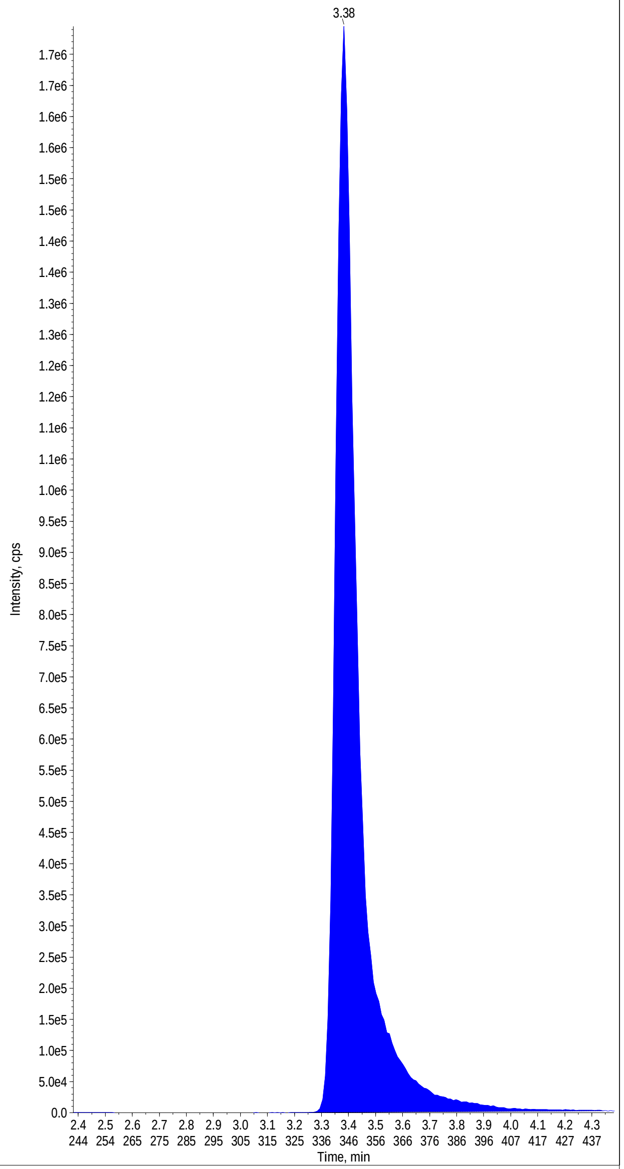

Sample Name: "LLOQ3" Sample ID: "" File: "20220801-1.wiff"
Peak Name: "OHTOL" Mass(es): "287.300/170.800 Da"
Comment: "" Annotation: ""

Sample Index: 38
Sample Type: Unknown
Concentration: N/A
Calculated Conc: 0.00 ng/mL
Acq. Date: 8/1/2022
Acq. Time: 3:48:44 PM

Modified: No
Proc. Algorithm: Specify Parameters - MQ III
Noise Percentage: 50
Base. Sub. Window: 1.00 min
Peak-Split. Factor: 2
Report Largest Peak: Yes
Min. Peak Height: 300.00 cps
Min. Peak Width: 0.00 sec
Smoothing Width: 9 points
RT Window: 30.0 sec
Expected RT: 2.14 min
Use Relative RT: No

Int. Type: Base To Base
Retention Time: 2.15 min
Area: 9.43e+004 counts
Height: 1.56e+004 cps
Start Time: 2.00 min
End Time: 2.57 min

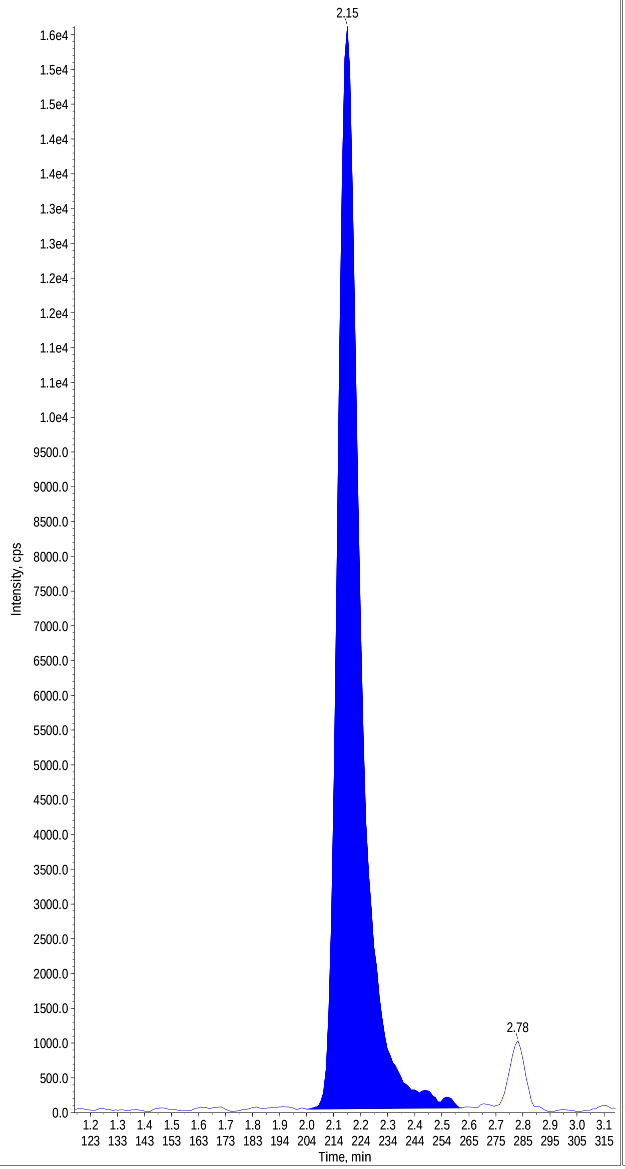

Sample Name: "LLOQ3" Sample ID: "" File: "20220801-1.wiff"
Peak Name: "DXP(IS)" Mass(es): "285.500/154.100 Da"
Comment: "" Annotation: ""

Sample Index: 38
Sample Type: Unknown
Concentration: 1.00 ng/mL
Calculated Conc: N/A
Acq. Date: 8/1/2022
Acq. Time: 3:48:44 PM

Modified: No
Proc. Algorithm: Specify Parameters - MQ III
Noise Percentage: 50
Base. Sub. Window: 1.00 min
Peak-Split. Factor: 2
Report Largest Peak: Yes
Min. Peak Height: 500.00 cps
Min. Peak Width: 0.00 sec
Smoothing Width: 0 points
RT Window: 30.0 sec
Expected RT: 3.38 min
Use Relative RT: No

Int. Type: Base To Base
Retention Time: 3.38 min
Area: 1.05e+007 counts
Height: 1.73e+006 cps
Start Time: 3.26 min
End Time: 4.31 min

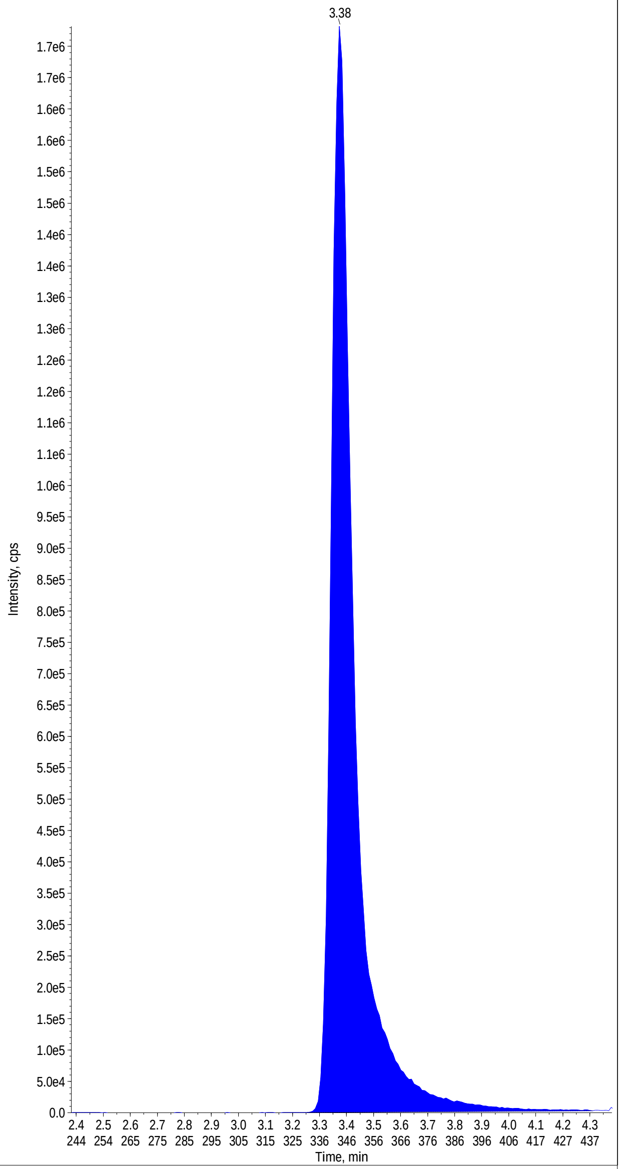

Sample Name: "LLOQ4" Sample ID: "" File: "20220801-1.wiff"
Peak Name: "OHTOL" Mass(es): "287.300/170.800 Da"
Comment: "" Annotation: ""

```
Sample Index:        39
Sample Type:         Unknown
Concentration:       N/A
Calculated Conc:     0.00     ng/mL
Acq. Date:           8/1/2022
Acq. Time:           3:53:47 PM

Modified:            No
Proc. Algorithm: Specify Parameters - MQ III
Noise Percentage:    50
Base. Sub. Window:   1.00   min
Peak-Split. Factor:  2
Report Largest Peak:  Yes
Min. Peak Height:    300.00   cps
Min. Peak Width:     0.00   sec
Smoothing Width:     9       points
RT Window:           30.0    sec
Expected RT:         2.14   min
Use Relative RT:     No

Int. Type:           Valley
Retention Time:      2.15   min
Area:                9.03e+004   counts
Height:              1.42e+004   cps
Start Time:          2.05   min
End Time:            2.43   min
```

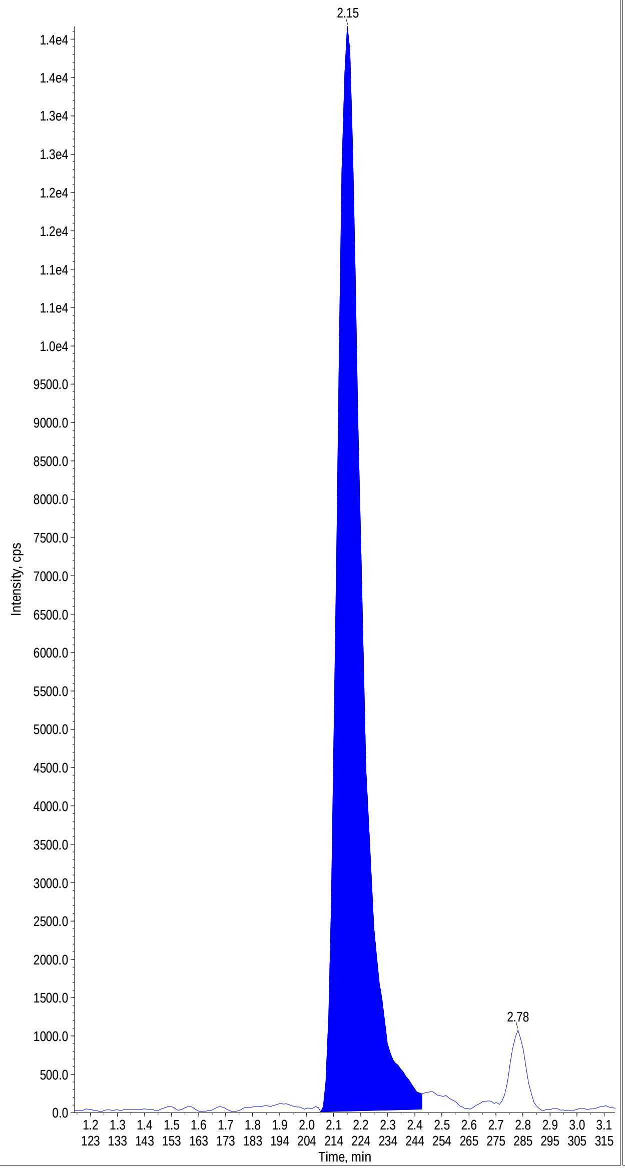

Sample Name: "LLOQ4" Sample ID: "" File: "20220801-1.wiff"
Peak Name: "DXP(IS)" Mass(es): "285.500/154.100 Da"
Comment: "" Annotation: ""

```
Sample Index:        39
Sample Type:         Unknown
Concentration:       1.00     ng/mL
Calculated Conc:     N/A
Acq. Date:           8/1/2022
Acq. Time:           3:53:47 PM

Modified:            No
Proc. Algorithm: Specify Parameters - MQ III
Noise Percentage:    50
Base. Sub. Window:   1.00   min
Peak-Split. Factor:  2
Report Largest Peak:  Yes
Min. Peak Height:    500.00   cps
Min. Peak Width:     0.00   sec
Smoothing Width:     0       points
RT Window:           30.0    sec
Expected RT:         3.38   min
Use Relative RT:     No

Int. Type:           Base To Base
Retention Time:      3.38   min
Area:                1.04e+007   counts
Height:              1.69e+006   cps
Start Time:          3.26   min
End Time:            4.31   min
```

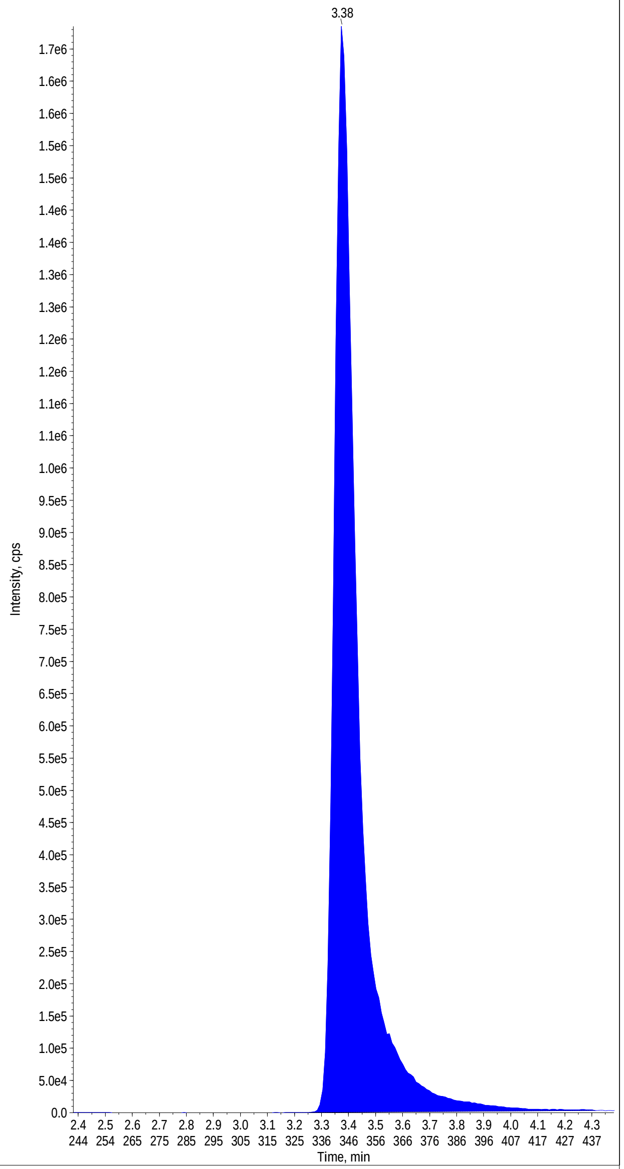

Sample Name: "LLOQ5" Sample ID: "" File: "20220801-1.wiff"
Peak Name: "OHTOL" Mass(es): "287.300/170.800 Da"
Comment: "" Annotation: ""

Sample Index:	40
Sample Type:	Unknown
Concentration:	N/A
Calculated Conc:	0.00 ng/mL
Acq. Date:	8/1/2022
Acq. Time:	3:58:48 PM
Modified:	No
Proc. Algorithm:	Specify Parameters - MQ III
Noise Percentage:	50
Base. Sub. Window:	1.00 min
Peak-Split. Factor:	2
Report Largest Peak:	Yes
Min. Peak Height:	300.00 cps
Min. Peak Width:	0.00 sec
Smoothing Width:	9 points
RT Window:	30.0 sec
Expected RT:	2.14 min
Use Relative RT:	No
Int. Type:	Valley
Retention Time:	2.15 min
Area:	8.98e+004 counts
Height:	1.50e+004 cps
Start Time:	2.05 min
End Time:	2.40 min

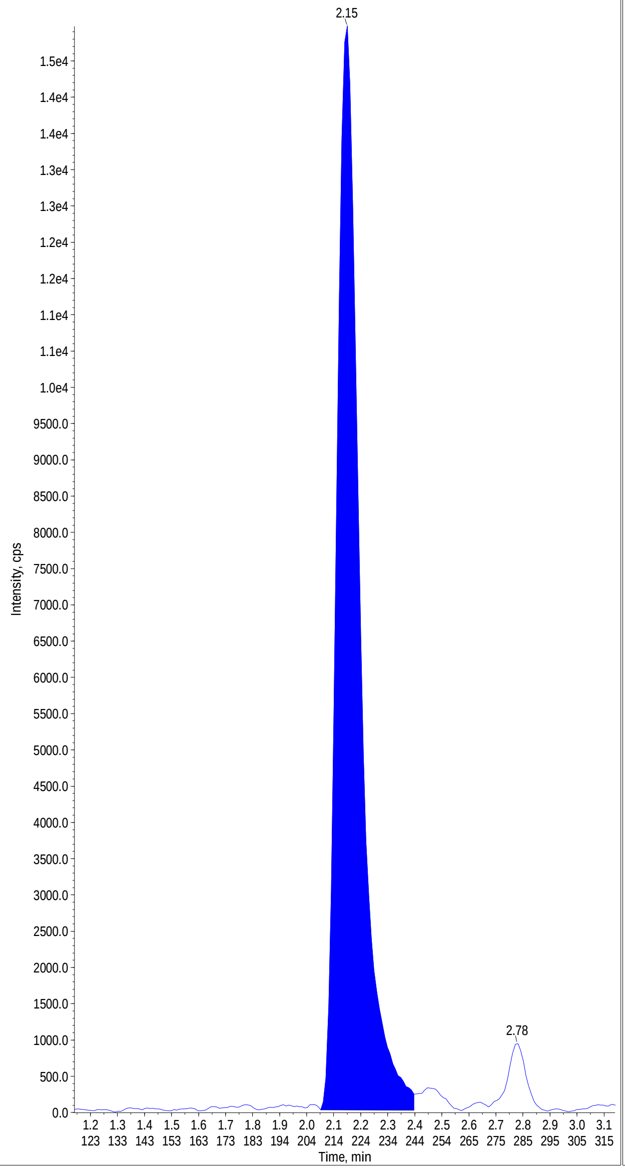

Sample Name: "LLOQ5" Sample ID: "" File: "20220801-1.wiff"
Peak Name: "DXP(IS)" Mass(es): "285.500/154.100 Da"
Comment: "" Annotation: ""

Sample Index:	40
Sample Type:	Unknown
Concentration:	1.00 ng/mL
Calculated Conc:	N/A
Acq. Date:	8/1/2022
Acq. Time:	3:58:48 PM
Modified:	No
Proc. Algorithm:	Specify Parameters - MQ III
Noise Percentage:	50
Base. Sub. Window:	1.00 min
Peak-Split. Factor:	2
Report Largest Peak:	Yes
Min. Peak Height:	500.00 cps
Min. Peak Width:	0.00 sec
Smoothing Width:	0 points
RT Window:	30.0 sec
Expected RT:	3.38 min
Use Relative RT:	No
Int. Type:	Base To Base
Retention Time:	3.37 min
Area:	1.05e+007 counts
Height:	1.77e+006 cps
Start Time:	3.24 min
End Time:	4.32 min

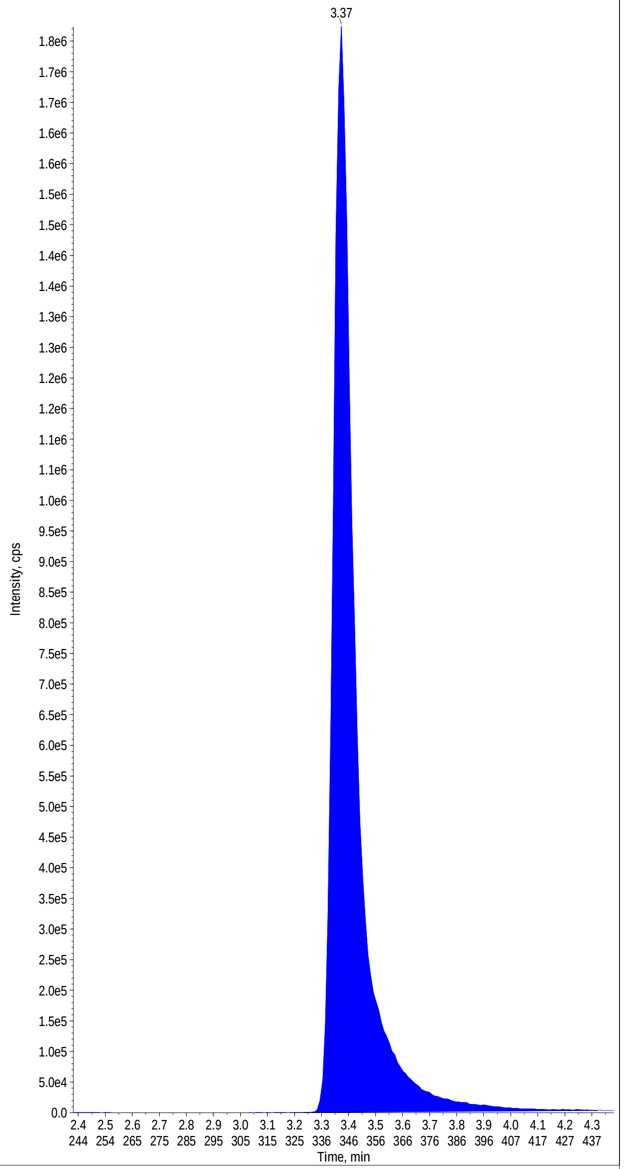

Sample Name: "LLOQ6" Sample ID: "" File: "20220801-1.wiff"
Peak Name: "OHTOL" Mass(es): "287.300/170.800 Da"
Comment: "" Annotation: ""

```
Sample Index:       41
Sample Type:       Unknown
Concentration:      N/A
Calculated Conc:    0.00    ng/mL
Acq. Date:         8/1/2022
Acq. Time:         4:03:50 PM

Modified:           No
Proc. Algorithm: Specify Parameters - MQ III
Noise Percentage:     50
Base. Sub. Window:    1.00   min
Peak-Split. Factor:   2
Report Largest Peak:   Yes
Min. Peak Height:    300.00   cps
Min. Peak Width:       0.00   sec
Smoothing Width:       9       points
RT Window:          30.0     sec
Expected RT:         2.14    min
Use Relative RT:     No

Int. Type:         Base To Base
Retention Time:      2.15    min
Area:           9.22e+004    counts
Height:          1.49e+004   cps
Start Time:          2.04    min
End Time:            2.56    min
```

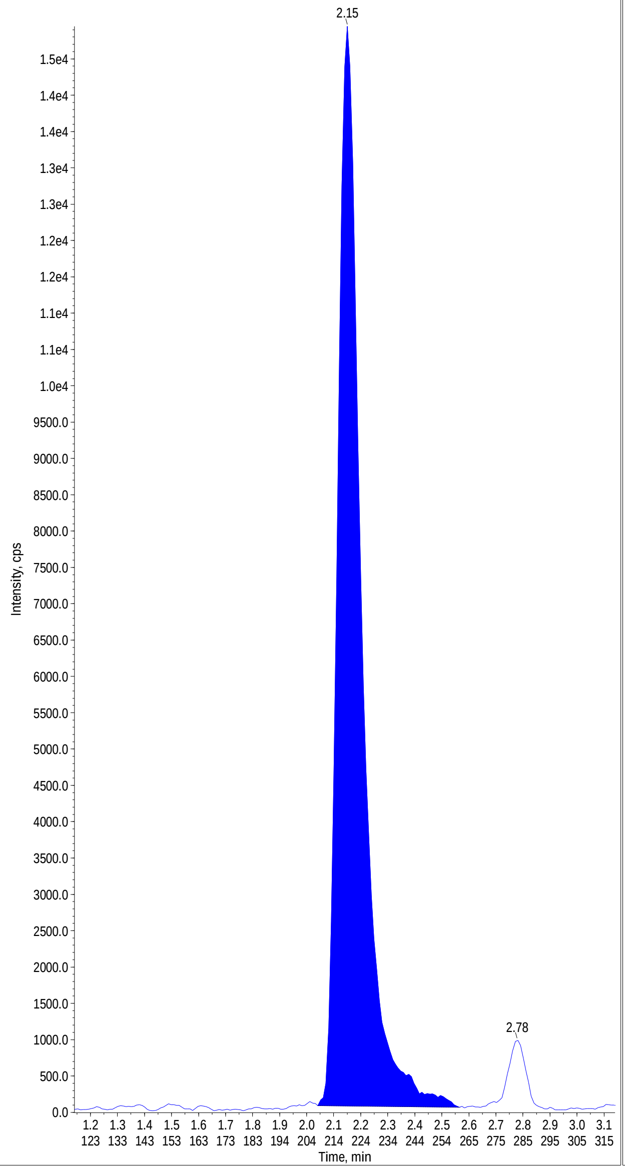

Sample Name: "LLOQ6" Sample ID: "" File: "20220801-1.wiff"
Peak Name: "DXP(IS)" Mass(es): "285.500/154.100 Da"
Comment: "" Annotation: ""

```
Sample Index:       41
Sample Type:       Unknown
Concentration:      1.00    ng/mL
Calculated Conc:    N/A
Acq. Date:         8/1/2022
Acq. Time:         4:03:50 PM

Modified:           No
Proc. Algorithm: Specify Parameters - MQ III
Noise Percentage:     50
Base. Sub. Window:    1.00   min
Peak-Split. Factor:   2
Report Largest Peak:   Yes
Min. Peak Height:    500.00   cps
Min. Peak Width:       0.00   sec
Smoothing Width:       0       points
RT Window:          30.0     sec
Expected RT:         3.38    min
Use Relative RT:     No

Int. Type:         Base To Base
Retention Time:      3.38    min
Area:           1.04e+007    counts
Height:          1.77e+006   cps
Start Time:          3.25    min
End Time:            4.32    min
```

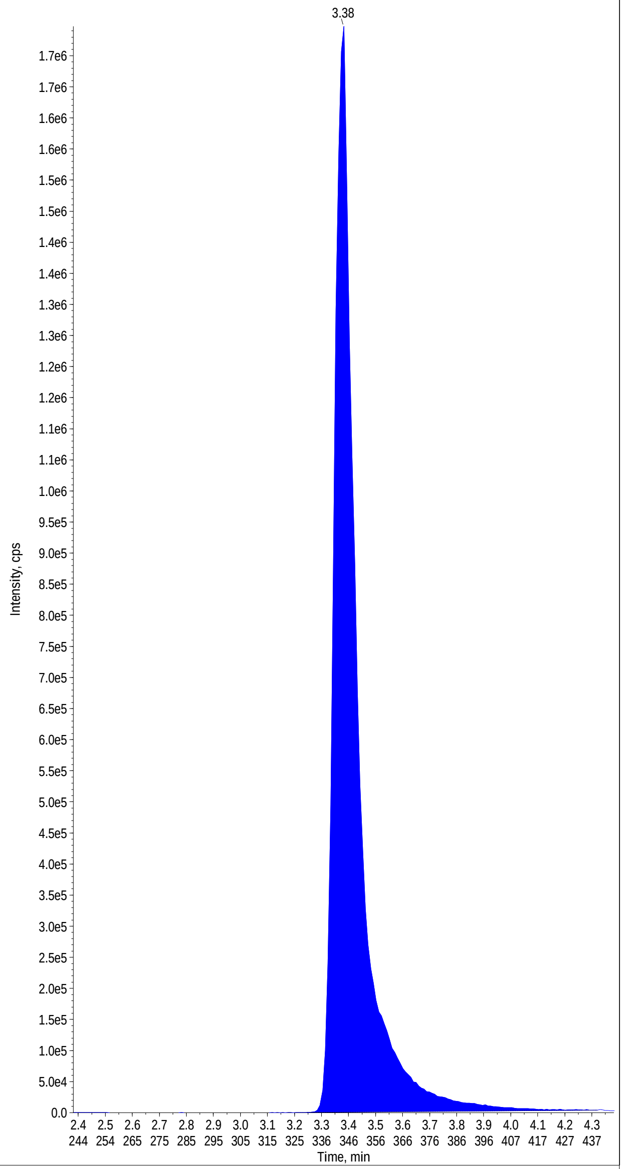

Sample Name: "MEOH"   Sample ID: ""   File: "20220801-1.wiff"
Peak Name: "OHTOL"   Mass(es): "287.300/170.800 Da"
Comment: ""   Annotation: ""

```
Sample Index:       42
Sample Type:      Unknown
Concentration:       N/A
Calculated Conc:     0.00     ng/mL
Acq. Date:        8/1/2022
Acq. Time:        6:50:32 PM

Modified:            No
```

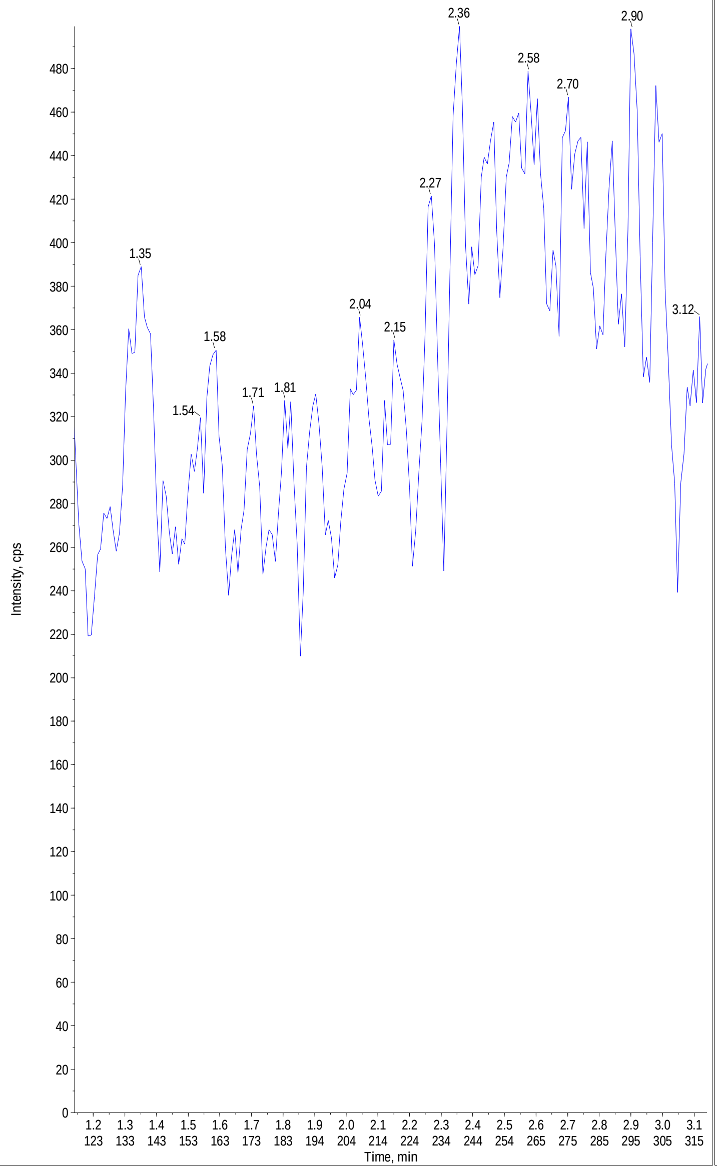

Sample Name: "MEOH"   Sample ID: ""   File: "20220801-1.wiff"
Peak Name: "DXP(IS)"   Mass(es): "285.500/154.100 Da"
Comment: ""   Annotation: ""

```
Sample Index:       42
Sample Type:      Unknown
Concentration:       1.00     ng/mL
Calculated Conc:     N/A
Acq. Date:        8/1/2022
Acq. Time:        6:50:32 PM

Modified:            No
Proc. Algorithm: Specify Parameters - MQ III
Noise Percentage:      50
Base. Sub. Window:     1.00    min
Peak-Split. Factor:    2
Report Largest Peak:    Yes
Min. Peak Height:     500.00    cps
Min. Peak Width:        0.00    sec
Smoothing Width:        0        points
RT Window:          30.0     sec
Expected RT:         3.38    min
Use Relative RT:     No

Int. Type:        Base To Base
Retention Time:      3.32    min
Area:           2.15e+004    counts
Height:          1.45e+003   cps
Start Time:          3.07    min
End Time:            3.73    min
```

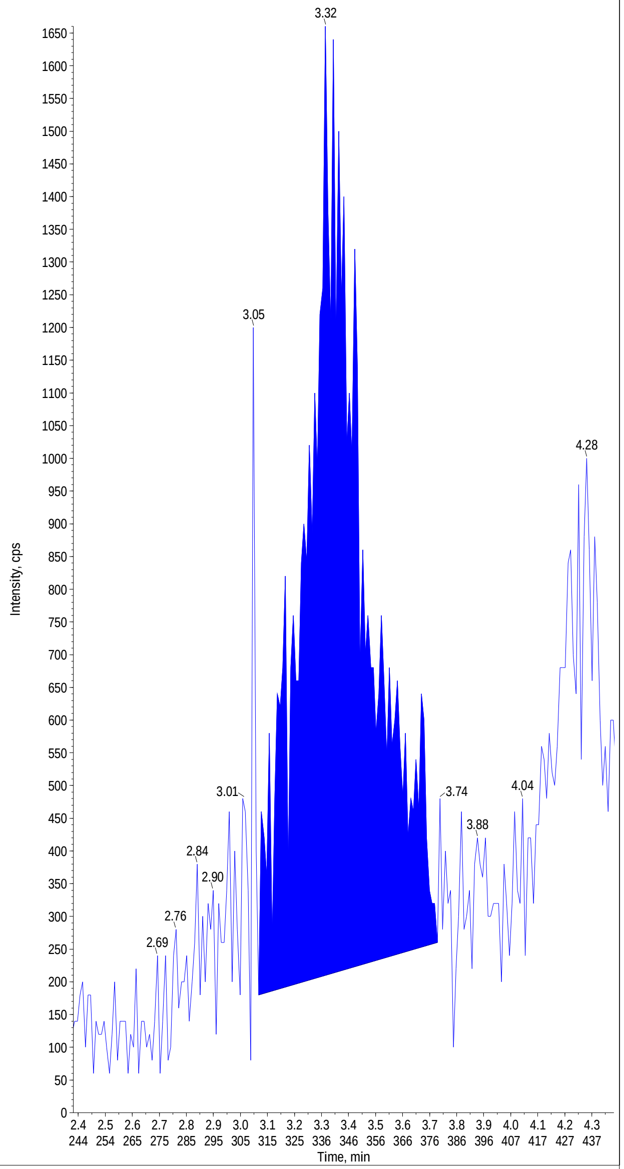

Supplement: Supplemental Information 1 — The raw data for sucrose preference, average moving speed, total moving distance, freezing time, times entering the central area, cortex 5-HT level, plasma clopidogrel level, pharmacokinetic parameters of clopidogrel in rats’ plasma , CYP450 activity and expression in rat livers (Fig. 2–Fig. 5, and Tables 2 and 3) [file peerj-10-14111-s001.zip › OHTOL.pdf]
